# Nucleophilic Functionalization of 2-R-3-Nitropyridines as a Versatile Approach to Novel Fluorescent Molecules

**DOI:** 10.3390/molecules27175692

**Published:** 2022-09-03

**Authors:** Vladislav V. Nikol’skiy, Mikhail E. Minyaev, Maxim A. Bastrakov, Alexey M. Starosotnikov

**Affiliations:** N.D. Zelinsky Institute of Organic Chemistry RAS, Leninsky Prosp. 47, 11991 Moscow, Russia

**Keywords:** nitro group, nitropyridines, bis-(het)aryl ethenes, nucleophilic substitution, thiols, UV–Vis spectroscopy

## Abstract

A number of new 2-methyl- and 2-arylvinyl-3-nitropyridines were synthesized and their reactions with thiols were studied. It was found that 3-NO_2_ tends to be selectively substituted under the action of sulfur nucleophiles in the presence of another nucleofuge in position 5. Correlations between the substitution pattern and regioselectivity as well as photophysical properties were established. Some synthesized compounds possessed a large Stokes shift.

## 1. Introduction

Pyridine is an important heterocyclic motif and a part of various natural products. The pyridine ring system is incorporated into alkaloids, medicines (for example, omeprazole, lorlatinib, ivosidenib and many others), fungicides, herbicides and insecticides. Application of the pyridine derivatives as biologically active precursors and coordination complexes was reviewed recently [1]. Nitropyridines are of particular interest due to their biological significance [2,3,4,5]. In addition, some nitropyridines are considered to be promising energetic compounds [6,7,8,9,10] and efficient organic optical materials [11]. The introduction of the nitro group into the pyridine ring facilitates its functionalization in different ways. Recently, we investigated reactions of 3-R-5-nitropyridines with various types of nucleophiles [12]. It was found that in the case of anionic S-, N- and O-nucleophiles, the substitution of the non-activated nitro group occurred while carbon nucleophiles underwent dearomatization of the pyridine ring with the formation of 1,2- or 1,4-addition products. As a result, a number of novel or hardly accessible pyridines and their dihydro derivatives were synthesized [12].

**Figure 1:**
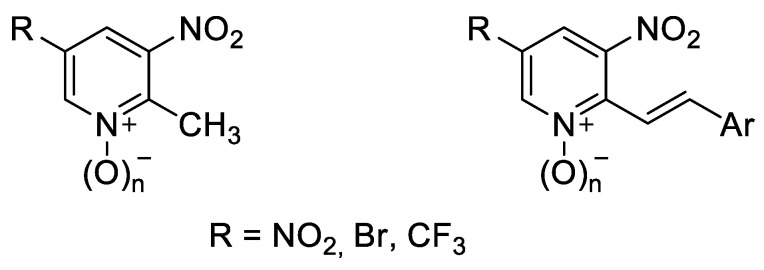
2-Methyl- and 2-(2-arylvinyl)-3-nitropyridines synthesized and studied in this work.

In this work we report on the synthesis, reactivity and photophysical properties of 2-methyl- and 2-(2-arylvinyl)-3-nitropyridines, as shown in Figure 1. 2-Alkenylpyridines are widely employed as precursors to pharmaceuticals (vorapaxar, axitinib, nifurpirinol) and other biologically active compounds [13]. In addition, 2-(2-arylvinyl)pyridines were proven to be the fluorescent molecules, with the fluorescence quantum yield showing a large dependence on the acidity of media [14].

## 2. Results and Discussion

**Scheme 1:**
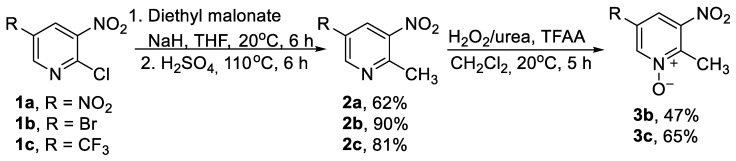
Synthesis of 2-methyl-3-nitropyridines.

2-Methyl-3-nitropyridines **2a**–**c** were synthesized from the corresponding commercially available 2-chloro-3-nitropyridines **1a**–**c** by the reaction with diethyl malonate, followed by acidic hydrolysis/decarboxylation, as shown in Scheme 1. The oxidation of compounds **2b**,**c** with the hydrogen peroxide–urea complex gave *N*-oxides **3b**,**c** in moderate yields.

**Scheme 2:**
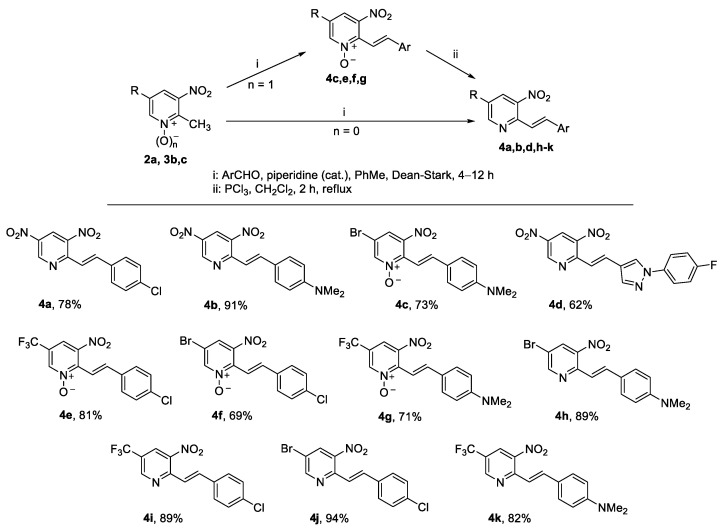
Synthesis of 2-(2-arylvinyl)-3-nitropyridines.

We examined 2-methylpyridines **2** and **3** in reactions with aldehydes under piperidine catalysis. Our attempts to isolate condensation products of compounds **2b**,**c** failed, whereas 2-methyl-3,5-dinitropyridine **2a** and *N*-oxides **3b**,**c** gave diarylethenes **4a**–**g** in high yields, as shown in Scheme 2. It should be noted that compound **2a** reacts several times faster than pyridine *N*-oxides **3b**,**c**, indicating that *para*-NO_2_ is a more potent activating group for this reaction than the *N*-oxide moiety neighboring the 2-methyl group. The functional group tolerance, along with the relatively mild conditions and availability of aromatic aldehydes, makes this method a valid alternative for Pd-catalyzed coupling reactions [15,16,17,18]. Deoxygenation of pyridine *N*-oxides **4c**,**e**–**g** with PCl_3_ allowed us to obtain four additional 2-ethenylpyridines **4h**–**k**, which were inaccessible via direct condensation of 2-methylpyridines **2b**,**c** with aldehydes, as shown in Scheme 2.

**Figure 2:**
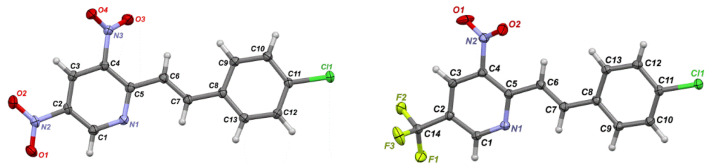
X-ray crystal structures of compounds **4a** (**left**) and **4i** (**right**) with thermal ellipsoids at 50% probability level.

In all cases, only *trans*-diarylethenes are formed, which was confirmed by NMR spectroscopy: coupling constants of 15–16 Hz were observed for proton signals of the double bonds. In addition, X-ray analysis for compounds **4a**,**i** was performed, undoubtedly proving our assumption, as shown in Figure 2.

**Scheme 3:**
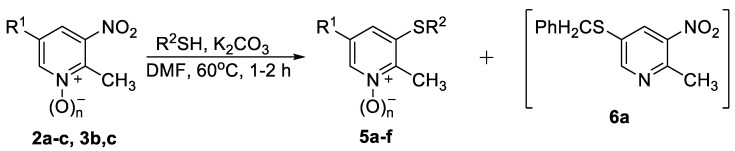
Reactions of 2-methyl-3-nitropyridines with thiols.

The possibility of the substitution of the non-activated nitro group in pyridines was studied recently by our group [12]. In 3-nitro-5-Cl(Br)-pyridines, 3-NO_2_ was found to be more nucleofugal than halogen in position 5. The reactions of 2-methyl-3-nitropyridines **2** and **3** with thiols are summarized in Scheme 3 and Table 1. Upon heating the reactants in DMF in the presence of K_2_CO_3_, the selective formation of 3-R^2^S-products **5** was observed in all cases; however, the reaction of **2a** with BnSH gave **5a** with a trace amount of the isomer **6a**.

**Scheme 4:**
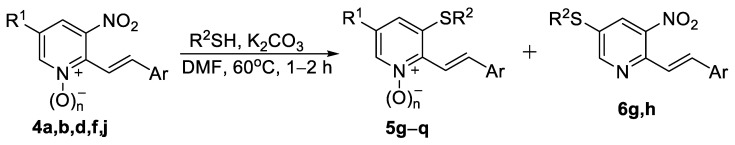
Reactions of 2-(arylvinyl)-3-nitropyridines with thiols.

Interestingly, diarylethenes **4** react with thiols under the same mild conditions, but with lower selectivity. ^1^H NMR spectra of the crude products generally contain an additional set of signals corresponding to the 5-R^2^S-isomer, whereas the ratio of **5**/**6** varied from 2:1 to 20:1 depending on the substrate and thiol. Moreover, compounds **6g**,**h** were isolated and fully characterized, but in all other cases we were unable to isolate isomers **6**, as shown in Scheme 4 and Table 2.

**Figure 3:**
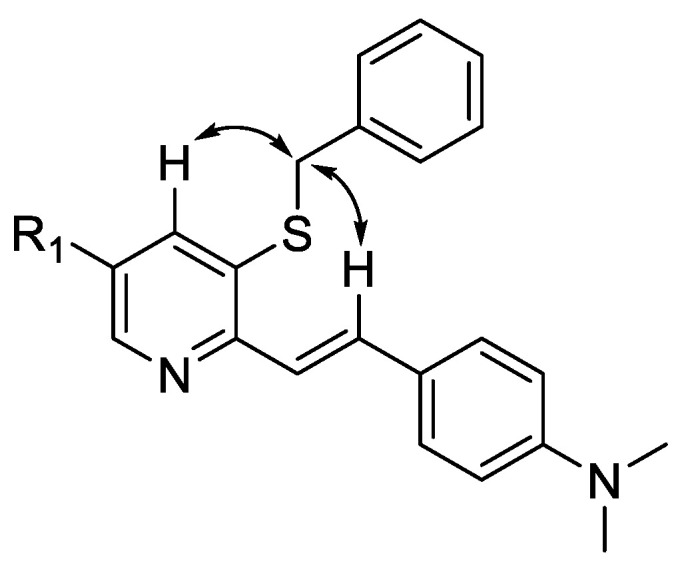
Cross-peaks observed in ^1^H-^1^H NOESY spectrum of compounds **5m** (R^1^ = NO_2_) and **5q** (R^1^ = Br).

**Figure 4:**
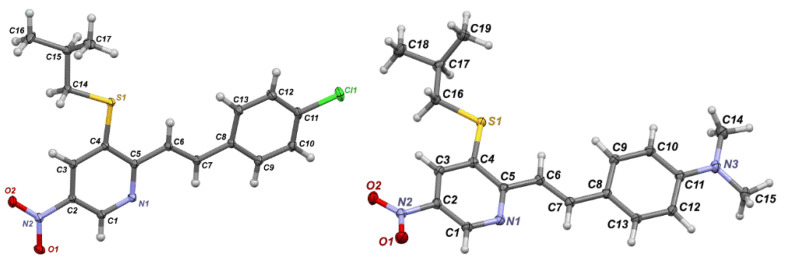
X-ray crystal structures of compounds **5h** (left) and **5l** (right) with thermal ellipsoids at 50% probability level.

Structures of compounds **5** and **6** were confirmed by NMR, HRMS, X-ray and elemental analysis. ^1^H-^1^H NOESY spectra of compounds **5m** and **5q** revealed interactions of the spatially close protons of the double bond and benzyl substituent, as shown in Figure 3. The structures of **5h,l** were determined by the X-ray diffraction single-crystal method, as shown in Figure 4.

The above-mentioned results allow us to conclude that electron-releasing substituents in the aryl group and the bulky thiolate anion favor substitution at position 3: the best selectivity was observed for reactions of **4b** with α-toluene thiol, whereas **4a** with isobutyl mercaptan gave the lowest selectivity (2:1). Reactions with 4-chlorothiophenol afforded exclusively a 3-substituted product.

**Figure 5:**
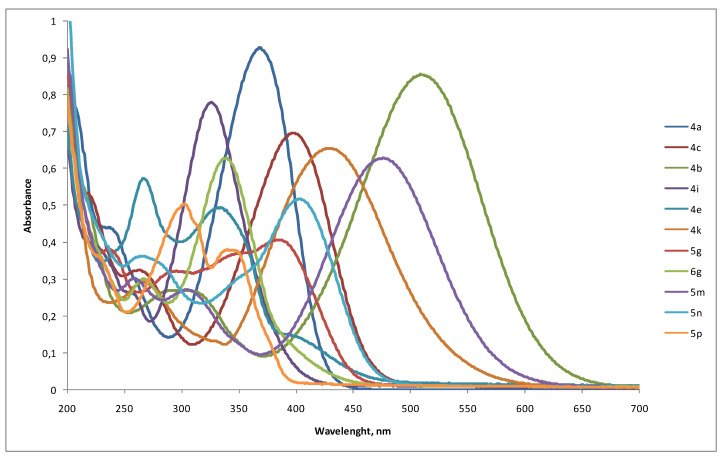
UV–Vis spectra of selected synthesized compounds in MeCN (2 × 10^−5^ M).

Compounds with multiple conjugated double bonds, such as diarylethenes **4**–**6**, can be expected to have strong absorbance in the UV and visible region; therefore, the photophysical properties of some representative compounds with various substitution patterns were studied. Indeed, it was found that all recorded UV–Vis spectra in the MeCN solution have a strong and distinctive absorption band in the 326-509 nm region accompanied by one or more weaker and non-informative bands around 260-300 nm (Figure 5, Table 3). Notable exceptions are compounds **4a**,**i** with only one dominant absorption maximum and compound **4e** with a stronger shortwave band, which can be attributed to the *N*-oxide moiety.

**Figure 6:**
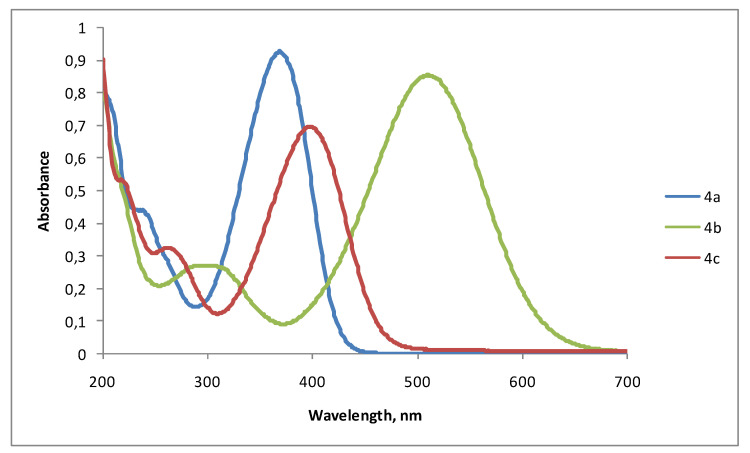
UV–Vis spectra of compounds **4a**–**c** in MeCN (2 × 10^−5^ M).

In the case of dinitro compounds **4a**–**c**, the electron-releasing Me_2_N group in the phenyl ring leads to a considerable red shift of the absorption maximum (by 141 nm) with respect to the electron-withdrawing chlorine atom (compounds **4a** and **4b**, Figure 6). Compound **4b**, with a strong electron-releasing 4-dimethylaminophenyl group, absorbs light in the visible region, whereas compounds **4a** and **4c** have their absorption maxima at the border between the visible and UV regions. This can be explained by the difference in the degree of charge transfer along the conjugation chain between the strongly withdrawing nitropyridine ring and the second electron-donating ring through the double bond.

**Figure 7:**
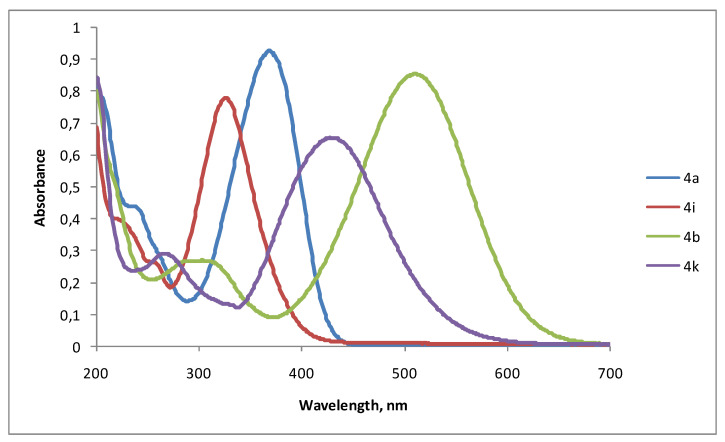
UV–Vis spectra of compounds **4a**,**b**,**i**,**k** in MeCN (2 × 10^−5^ M).

On the other hand, the replacement of 5-NO_2_ with the CF_3_ group in a pyridine cycle caused a 42 nm blue shift for compounds **4a**/**4i** and an 80 nm shift for 4-dimethylaminophenyl derivatives **4b**/**4k**, as well as a small decrease in molar absorptivity (Figure 7). It can be concluded that substituents at the double bond as well as in position 5 can be independently altered to predictably fine-tune absorption spectra of these compounds.

**Figure 8:**
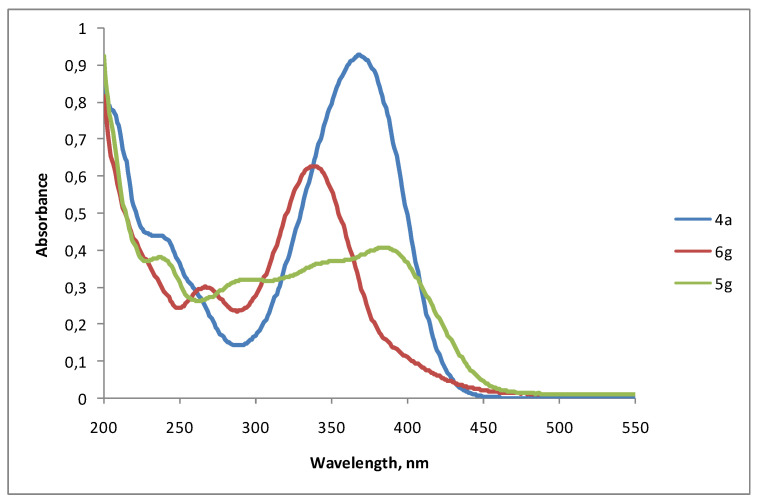
UV–Vis spectra of compounds **4a**, **5g**, **6g** in MeCN (2 × 10^−5^ M).

The study of isomeric substitution products of 3- and 5-NO_2_ in compound **4a** revealed an important dependence, as shown in Figure 8. Substitution of the nitro group at position 5 gave compound **6g**, whose absorption spectrum generally resembles that of the parent compound and follows the same pattern described above for the 5-NO_2_/5-CF_3_ pair. On the other hand, substitution of the nitro group at position 3 gave compound **5g**, which is qualitatively different from both compounds. In this case, the absorption maximum shifts slightly towards the visible region, and the absorption spectrum itself acquires a more complex structure. From this we can conclude that the combination of 2-alkenyl and 3-alkylthio substituents leads to the appearance of a characteristic electronic structure. A similar pattern was observed for the substitution of 3-NO_2_ in compound 4c, but not for compound **4b**, which can be explained by the predominance of a strong charge transfer over the finer electronic structure. It should be noted that the alkylthio substituent does not significantly affect the photophysical properties of the obtained compounds.

**Figure 9:**
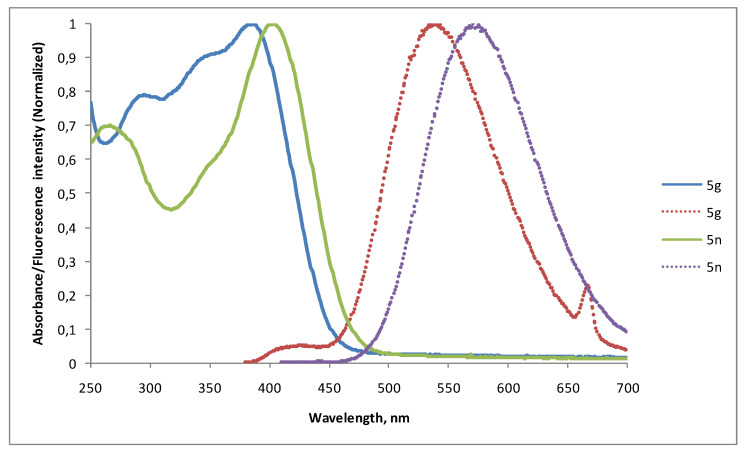
Normalized absorption and emission spectra of compounds **5g** and **5n** in MeCN.

**Figure 10:**
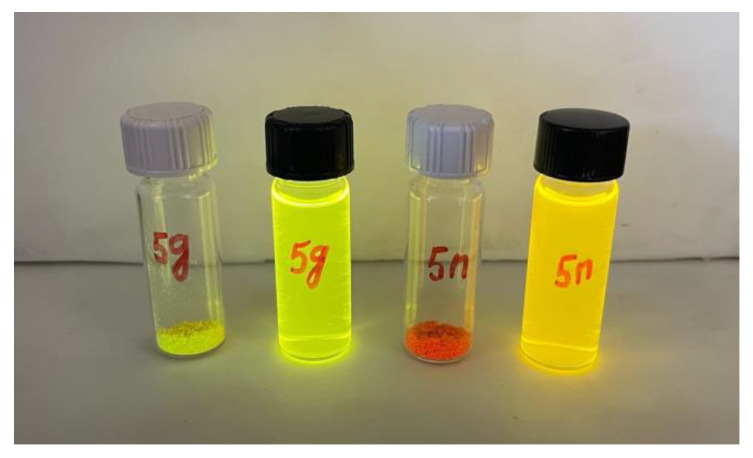
Fluorescence of compounds **5g** and **5n** in solid state and in MeCN solution under 365 nm UV lamp.

Compounds **5g**,**n** showed fluorescence upon excitation by light with the wavelength equal to the λ_max_ in the visible region, as shown in Figure 9 and Figure 10. Compound **5g** has an emission maximum at 538 nm and a Stokes shift of 154 nm, whereas for compound **5n**, these values are 571 nm and 168 nm, respectively. The large values of Stokes shifts (150–170 nm) almost completely eliminate the overlap between the absorption and emission regions. In addition, the properties of these fluorescent molecules can be tuned by changing the substituent at the double bond.

## 3. Conclusions

In conclusion, a number of the previously unknown 2-methyl- and 2-arylvinyl-3-nitropyridines were synthesized. Their reactions with *S*-nucleophiles proceeded under mild conditions and were found to be regioselective with a predominance of 3-NO_2_ substitution; the influence of the substituents in positions 2 and 5 on the observed selectivity was revealed. The synthesized compounds showed promising tunable photophysical properties, such as large Stokes shifts. The reported synthetic approach can be considered as a convenient tool for rapid access to pyridine-based building blocks of various potential applications.

## 4. Materials and Methods

### 4.1. General Information

All chemicals were of commercial grade and used directly without purification. Melting points were measured on a Stuart SMP20 apparatus (Stuart (Bibby Scientific), UK). ^1^H and ^13^C NMR spectra were recorded on a Bruker AM-300 (at 300.13 and 75.13 MHz, respectively, Bruker Biospin, Germany) or Bruker Avance DRX 500 (at 500 and 125 MHz, respectively, Bruker Biospin, Germany) in DMSO-d_6_ or CDCl_3_. *J* values are given in Hz. HRMS spectra were recorded on a Bruker micrOTOF II mass spectrometer using ESI. UV–Vis absorption spectra were recorded in MeCN (2 × 10^−5^ M) in standard 10 × 10 × 45 mm quartz cuvettes on a Cary 60 UV–Vis spectrophotometer (Agilent Technologies, Santa Clara, CA, USA). Fluorescence spectra were recorded in MeCN (2 × 10^−6^ M) in standard 10 × 10 × 45 mm quartz cuvettes on a Cary Eclipse fluorescence spectrophotometer (Agilent Technologies). All reactions were monitored by TLC analysis using ALUGRAM SIL G/UV254 plates, which were visualized with UV light. Compounds **1a**–**c** were purchased from commercial suppliers. In some cases we were unable to record the ^13^C NMR spectra of products due to insufficient solubility in common organic solvents (compounds **4d**, **5**,**i**,**j**,**o** and **6j**).

### 4.2. General Procedure for the Synthesis of 2-Methyl-3-nitropyridines ***2a**–**c***

To a stirred suspension of NaH (60% in mineral oil, 0.80 g, 20 mmol) in anhydrous THF (30 mL), diethyl malonate (1.52 mL, 10 mmol) was added dropwise. The suspension was stirred for 15 min until hydrogen evolution ceased and a solution of the corresponding 2-chloropyridine **1** (10 mmol) in THF (20 mL) was added. The reaction mixture was stirred at r.t. (room temperature) for 6 h, poured in water (200 mL) and acidified with conc. HCl to pH 3. This was then extracted with CHCl_3_, evaporated and 50% H_2_SO_4_ (30 mL) was added to the residue. The mixture was stirred for 6 h at 120 °C, cooled, neutralized with Na_2_CO_3_ to pH 8 and extracted with CHCl_3_. The organic phase was dried over Na_2_SO_4_, evaporated and the residue was purified via column chromatography (SiO_2_/CHCl_3_).

*2-Methyl-3,5-dinitropyridin* (**2a**) [19], brown oil; yield 62%; ^1^H NMR (300 MHz, CDCl_3_): δ 9.55 (d, 1H, *J* = 2.1 Hz), 9.06 (d, 1H, *J* = 2.1 Hz), 3.03 (s, 3H).

*5-Bromo-2-methyl-3-nitropyridin* (**2b**) [20], yellowish oil; yield 90%; 1H NMR (300 MHz, CDCl_3_): δ 8.80 (d, 1H, *J* = 2.1 Hz), 8.44 (d, 1H, *J* = 2.1 Hz), 2.84 (s, 3H).

*2-Methyl-3-nitro-5-trifluoromethylpyridine* (**2c**), yellowish oil; yield 81%; 1H NMR (300 MHz, CDCl_3_): δ 9.00 (s, 1H), 8.54 (s, 1H), 2.97 (s, 3H).

### 4.3. General Procedure for the Oxidation of 2-Methyl-3-nitropyridines ***2b**,**c***

To a solution of the corresponding 2-methyl-3-nitropyridine **2** (10 mmol) in CH_2_Cl_2_ (30 mL), a freshly prepared complex urea/H_2_O_2_ (1.88 g, 20 mmol) was added. The resulting suspension was cooled to 0 °C and trifluoroacetic anhydride (5 mL, 36 mol) was added dropwise. The reaction mixture was stirred for 30 min at 0 °C and 4 h at r.t. A saturated aqueous solution of Na_2_S_2_O_3_ (50 mL) was added and the organic phase was separated. An aqueous layer was additionally extracted with CH_2_Cl_2_ and combined organic solutions were washed with the saturated solution of NaHCO_3_, dried over Na_2_SO_4_ and evaporated. The residue was recrystallized from aqueous EtOH.

*5-Bromo-2-methyl-3-nitropyridine N-oxide* (**3b**)**,** pale-yellow solid; yield 47%; mp 104–105 °C; ^1^H NMR (300 MHz, CDCl_3_): δ 8.60 (s, 1H), 7.86 (s, 1H), 2.67 (s, 3H, CH_3_). ^13^C NMR (125 MHz, CDCl_3_): δ 148.5, 145.5, 143.5, 122.9, 116.4, 13.8. HRMS (ESI, *m/z*): calcd for C_6_H_5_BrN_2_O_3_ [M + H]^+^: 232.9556; found: 232.9563.

*2-Methyl-3-nitro-5-trifluoromethylpyridine N-oxide* (**3c**), pale-yellow solid; yield 65%; mp 82–83 °C; ^1^H NMR (300 MHz, CDCl_3_): δ 8.69 (s, 1H), 7.89 (s, 1H), 2.75 (s, 3H). ^13^C NMR (75 MHz, CDCl_3_): δ 149.9, 148.8, 139.5, 127.1 (q, ^2^J_CF_ = 36 Hz), 121.1 (q, ^1^J_CF_ = 274 Hz), 116.1, 14.3. HRMS (ESI, *m/z*): calcd for C_7_H_5_F_3_N_2_O_3_ [M + H]^+^: 223.0325; found: 223.0331.

### 4.4. General Procedure for the Synthesis of Compounds ***4a**–**g***

To a solution of the corresponding 2-methylpyridine **2** or **3** (5 mmol) in toluene (30 mL), aromatic aldehyde (5 mmol) and 50 μL of piperidine was added. The reaction mixture was stirred under reflux with a Dean–Stark adapter until water separation was completed. The solvent was evaporated and the residue was triturated with 20 mL of cold EtOH. The precipitate was filtered off and air-dried.

*(E)-2-[(4-Chlorophenyl)vinyl]-3,5-dinitropyridine* (**4a**), yellow solid; yield 78%; mp 204–205 °C; ^1^H NMR (300 MHz, CDCl_3_): δ 9.58 (s, 1H), 9.05 (s, 1H), 8.30 (d, 1H, *J* = 15.3 Hz), 7.83 (d, 1H, *J* = 15.3 Hz), 7.64 (d, 2H, *J* = 8.4 Hz), 7.45 (d, 2H, *J* = 8.4 Hz). ^13^C NMR (150 MHz, DMSO-d_6_): δ 152.0, 147.5, 143.2, 141.9, 140.3, 135.0, 134.0, 130.2, 129.2, 129.1, 121.6. HRMS (ESI, *m/z*): calcd for C_13_H_8_ClN_3_O_4_ [M + H]^+^: 306.0276; found: 306.0283.

*(E)-4-(2-(3,5-Dinitropyridin-2-yl)vinyl)-N,N-dimethylaniline* (**4b**), dark-violet solid; yield 91%; mp 260 °C (dec.); ^1^H NMR (300 MHz, CDCl_3_): δ 9.41 (d, 1H, *J* = 2.1 Hz), 8.93 (d, 1H, *J* = 2.1 Hz), 8.28 (d, 1H, *J* = 15.0 Hz), 7.64 (d, 1H, *J* = 15.0 Hz), 7.57 (d, 2H, *J* = 8.8 Hz), 6.77 (d, 2H, *J* = 8.8 Hz), 3.06 (s, 6H). ^13^C NMR (125 MHz, CDCl_3_): δ 154.8, 152.4, 147.3, 146.4, 139.5, 131.1, 128.9, 123.2, 114.4, 111.9, 40.1. HRMS (ESI, *m/z*): calcd for C_15_H_14_N_4_O_4_ [M + H]^+^: 315.1088; found: 315.1085.

*(E)-5-Bromo-2-(4-dimethylaminophenyl)vinyl-3-nitropyridine N-oxide* (**4c**), red-brown solid; yield 73%; mp 217 °C (dec.); ^1^H NMR (300 MHz, CDCl_3_): δ 8.55 (d, 1H, *J* = 15.9 Hz), 8.49 (s, 1H), 7.64 (s, 1H), 7.49 (d, 2H, *J* = 8.7 Hz), 7.00 (d, 1H, *J* = 15.6 Hz), 6.69 (d, 2H, *J* = 8.7 Hz), 3.05 (s, 6H). ^13^C NMR (75 MHz, CDCl_3_): δ 151.8, 147.2, 143.9, 142.8, 141.2, 129.9, 123.8, 123.0, 113.0, 111.9, 107.1, 40.1. HRMS (ESI, *m/z*): calcd for C_15_H_14_BrNO_3_ [M + H]: 364.0291; found: 364.0292.

*(E)-2-(2-(1-(4-Fluorophenyl)-1H-pyrazol-4-yl)vinyl)-3,5-dinitropyridine* (**4d**), brown solid; yield 62%; mp 255 °C; ^1^H NMR (300 MHz, CDCl_3_): δ 9.53 (d, 1H, *J* = 2.1 Hz), 9.03 (d, 1H, *J* = 2.1 Hz), 8.30 (d,1H, *J* = 15.3 Hz), 8.19 (s, 1H), 8.06 (s, 1H), 7.70 (m, 3H), 7.20 (m, 2H). HRMS (ESI, *m/z*): calcd for C_16_H_10_FN_5_O_4_ [M + H]^+^: 356.0790; found: 356.0789.

(*E)-2-(4-Chlorophenyl)vinyl-3-nitro-5-trifluoromethylpyridine N-oxide* (**4e**), yellow solid; yield 81%; mp 161–162 °C; ^1^H NMR (300 MHz, CDCl_3_): δ 8.65 (d,1H, *J* = 15.9 Hz), 8.64 (s, 1H), 7.73 (s, 1H), 7.52 (d, 2H, *J* = 8.4 Hz), 7.39 (d, 2H, *J* = 8.4 Hz), 7.17 (d, 1H, *J* = 15.9 Hz). ^13^C NMR (75 MHz, CDCl_3_): δ 147.9, 143.6, 142.3, 140.4, 136.7, 134.1, 129.4, 129.3, 125.5 (q, ^2^J_CF_ = 36 Hz), 121.1 (q, ^1^J_CF_ = 273 Hz), 116.0 (d, ^3^J_CF_ = 3.2 Hz), 112.8. HRMS (ESI, *m/z*): calcd for C_14_H_8_ClF_3_N_2_O_3_ [M + H]^+^: 345.0248; found: 345.0239.

*(E)-5-Bromo-2-(4-chlorophenyl)vinyl-3-nitropyridine N-oxide* (**4f**), yellow solid; yield 69%; mp 173–174 °C; ^1^H NMR (300 MHz, CDCl_3_): δ 8.45 (s, 1H), 8.30 (d, 1H, *J* = 16.1 Hz), 7.60 (s, 1H), 7.41 (d, 2H, *J* = 8.4 Hz), 7.29 (d, 2H, *J* = 8.4 Hz), 7.02 (d, 1H, *J* = 16.1 Hz). ^13^C NMR (125 MHz, CDCl_3_): δ 147.8, 144.1, 140.3, 140.2, 136.1, 134.4, 129.2, 129.1, 122.7, 115.7, 113.1. HRMS (ESI, *m/z*): calcd for C_13_H_8_BrClN_2_O_3_ [M + H]^+^: 354.9480; found: 354.9472.

*(E)-2-(4-Dimethylaminophenyl)vinyl-3-nitro-5-trifluoromethylpyridine N-oxide* (**4g**), red-brown solid; yield 71%; mp 198-199 °C; ^1^H NMR (300 MHz, CDCl_3_): δ 8.85 (d, 1H, *J* = 15.6 Hz), 8.58 (s, 1H), 7.68 (s, 1H), 7.52 (d, 2H, *J* = 8.4 Hz), 7.10 (d, 1H, *J* = 15.6 Hz), 6.69 (d, 2H, *J* = 8.4 Hz), 3.06 (s, 6H). ^13^C NMR (125 MHz, CDCl_3_): δ 152.2, 146.8, 145.1, 144.4, 140.1, 130.5, 123.4, 122.7 (q, ^2^J_CF_ = 36 Hz), 121.4 (q, ^1^J_CF_ = 273 Hz), 116.3 (q, ^3^J_CF_ = 3.6 Hz), 111.9, 106.6, 40.0. HRMS (ESI, *m/z*): calcd for C_16_H_14_F_3_N_3_O_3_ [M + H]^+^: 354.1060; found: 354.1060.

### 4.5. General Procedure for the Synthesis of Compounds ***4h**–**k***

To a solution of the corresponding pyridine N-oxide (0.5 mmol), CH_2_Cl_2_ (10 mL) was added PCl_3_ (0.13 mL) and the mixture was stirred under reflux for 2 h. After cooling, the reaction was washed with a saturated NaHCO_3_ solution, dried over Na_2_SO_4_ and evaporated.

*(E)-4-(2-(5-Bromo-3-nitropyridin-2-yl)vinyl)-N,N-dimethylaniline* (**4h**), dark-brown solid; yield 89%; mp 202–203 °C; ^1^H NMR (300 MHz, CDCl_3_): δ 8.75 (s, 1H), 8.34 (s, 1H), 8.05 (d, 1H, *J* = 15.3 Hz), 7.55 (d, 1H, *J* = 15.6), 7.53 (d, 2H, *J* = 8.4), 6.71 (d, 2H, *J* = 8.4 Hz), 3.04 (s, 6H). ^13^C NMR (75 MHz, CDCl_3_): δ 153.8, 151.3, 148.8, 145.5, 142.8, 140.5, 135.0, 129.8, 115.5, 115.5, 112.3, 40.4, 29.7. HRMS (ESI, *m/z*): calcd for C_15_H_14_BrN_3_O_2_ [M + H]^+^: 348.0342; found: 348.0342.

*(E)-2-(4-Chlorophenyl)vinyl-3-nitro-5-trifluoromethylpyridine* (**4i**), yellow solid; yield 89%; mp 138 °C; ^1^H NMR (300 MHz, CDCl_3_): δ 9.02 (s, 1H), 8.50 (s, 1H), 8.16 (d, 1H, *J* = 15.6 Hz), 8.77 (d, 1H, *J* = 15.6 Hz), 7.59 (d, 2H, *J* = 8.4 Hz), 7.41 (d, 2H, *J* = 8.4 Hz). ^13^C NMR (75 MHz, CDCl_3_): δ 152.0, 149.2 (q, ^3^J_CF_ = 3.6 Hz), 143.0, 140.8, 136.1, 133.9, 130.7 (q, ^3^J_CF_ = 3.6 Hz), 129.4, 129.3, 124.9 (q, ^2^J_CF_ = 35 Hz), 120.6. HRMS (ESI, *m/z*): calcd for C_14_H_8_ClF_3_N_2_O_2_ [M + H]^+^: 329.0299; found: 329.0311.

*(E)-5-Bromo-2-(4-chlorostyryl)-3-nitropyridine* (**4j**), yellow solid; yield 94%; mp 184–185 °C; ^1^H NMR (300 MHz, CDCl_3_): δ 8.83 (d, 1H, *J* = 1.8 Hz), 8.40 (d, 1H, *J* = 1.8 Hz), 8.02 (d, 1H, *J* = 15.6 Hz), 7.68 (d, 1H, *J* = 15.6 Hz), 7.56 (d, 2H, *J* = 8.4 Hz), 7.38 (d, 2H, *J* = 8.4 Hz). ^13^C NMR (75 MHz, CDCl_3_): δ 154.0, 147.4, 143.7, 138.4, 135.5, 135.1, 134.2, 129.2, 129.1, 124.9, 117.6. HRMS (ESI, *m/z*): calcd for C_13_H_8_BrClN_2_O_2_ [M + H]^+^: 338.9530; found: 338.9533.

*(E)-N,N-Dimethyl-4-(2-(3-nitro-5-trifluoromethylpyridin-2-yl)vinyl)aniline* (**4k**), brown solid; yield 82%; mp 150–151 °C; ^1^H NMR (300 MHz, CDCl_3_): δ 8.40 (s, 1H), 8.36 (s, 1H), 8.12 (d, 1H, *J* = 15.3 Hz), 7.56 (d, 1H, *J* = 15.3 Hz), 7.49 (d, 2H, *J* = 8.7 Hz), 6.66 (d, 2H, *J* = 8.7 Hz), 2.99 (s, 6H). ^13^C NMR (75 MHz, CDCl_3_): δ 153.4, 151.9, 149.1 (q, ^3^*J_CF_* = 4 Hz), 143.3, 141.8, 130.8 (q, ^3^*J_CF_* = 4 Hz), 130.3, 122.8 (q, ^1^*J_CF_* = 271 Hz), 123.6, 122.9 (q, ^2^J_CF_ = 34 Hz), 115.0, 112.1, 40.2. HRMS (ESI, *m/z*): calcd for C_16_H_14_F_3_N_3_O_2_ [M + H]^+^: 338.1111; found: 338.1120.

### 4.6. General Procedure for the Synthesis of Compounds ***5*** and ***6***

To a solution of compound **2, 3** or **4** (1 mmol) in anhydrous DMF (5 mL), thiol (1 mmol) and K_2_CO_3_ (0.138 g, 1 mmol) were added. The reaction mixture was stirred for 1-2 h at 60 °C, poured into H_2_O (50 mL), acidified with conc. HCl to pH 3 and extracted with CHCl_3_ (3 × 20 mL). The organic phase was dried over Na_2_SO_4_, evaporated and the residue was purified via column chromatography (SiO_2_/CHCl_3_) or recrystallized from EtOH.

*3-(Benzylsulfanyl)-2-methyl-5-nitropyridine* (**5a**), beige solid; yield 70%; mp 79–80 °C; ^1^H NMR (300 MHz, CDCl_3_): δ 9.06 (d, 1H, *J* = 2.1 Hz), 7.35 (m, 5H), 4.24 (s, 2H, CH_2_), 2.65 (s, 3H, CH_3_). ^13^C NMR (125 MHz, CDCl_3_): δ 162.7, 142.8, 140.1, 134.9, 134.8, 128.9, 128.8, 128.0, 127.8, 37.1, 23.4. HRMS (ESI, *m/z*): calcd for C_13_H_12_N_2_O_2_S [M + H]^+^: 261.0692; found: 261.0699.

*3-(Benzylsulfanyl)-5-bromo-2-methylpyridine N-oxide* (**5b**), grey needles; yield 96%; mp 106–107 °C; ^1^H NMR (300 MHz, CDCl_3_): δ 8.25 (s, 1H), 7.26-7.32 (m, 5H), 7.24 (s, 1H), 4.14 (s, 2H, SCH_2_), 2.49 (s, 3H, CH_3_). ^13^C NMR (75 MHz, CDCl_3_): δ 147.3, 137.8, 136.8, 134.9, 128.9, 128.1, 127.7, 116.3, 38.5, 14.5. HRMS (ESI, *m/z*): calcd for C_13_H_12_BrNOS [M + H]^+^: 309.9896; found: 309.9896.

*5-Bromo-3-(4-chlorophenylsulfanyl)-2-methylpyridine N-oxide* (**5c**), brown solid; yield 95%; mp 131–132 °C; ^1^H NMR (300 MHz, CDCl_3_): δ 8.27 (s, 1H), 7.44 (d, 2H, *J* = 8.4 Hz), 7.38 (d, 2H, *J* = 8.4 Hz), 6.89 (s, 1H), 2.56 (s, 3H). ^13^C NMR (75 MHz, CDCl_3_): δ 146.7, 138.1, 137.3, 136.0, 134.8, 130.4, 129.0, 127.7, 116.6, 14.6. HRMS (ESI, *m/z*): calcd for C_12_H_9_BrClNOS [M + H]^+^: 329.9350; found: 329.9349.

*3-(Benzylsulfanyl)-5-bromo-2-methylpyridine* (**5d**), pale-yellow crystals; yield 65%; mp 55 °C; ^1^H NMR (300 MHz, CDCl_3_): δ 8.33 (s, 1H), 7.58 (s, 1H), 7.31 (m, 5H), 4.09 (s, 2H, SCH_2_), 2.48 (s, 3H, CH_3_). ^13^C NMR (75 MHz, CDCl_3_): δ 155.5, 146.5, 137.3, 135.6, 134.4, 128.9, 128.8, 127.8, 117.9, 37.8, 22.5. HRMS (ESI, *m/z*): calcd for C_13_H_12_BrNS [M + H]^+^: 293.9947; found: 293.9956.

*3-(Benzylsulfanyl)-2-methyl-5-(trifluoromethyl)pyridine* (**5e**), yellow oil; yield 60%; ^1^H NMR (300 MHz, CDCl_3_): δ 8.53 (s, 1H), 7.64 (s, 1H), 7.31 (s, 5H), 4.14 (s, 2H, SCH_2_), 2.61 (s, 3H, CH_3_). ^13^C NMR (75 MHz, CDCl_3_): δ 161.0, 142.3 (q, ^3^J_CF_ = 4 Hz), 135.5, 133.4, 131.7 (q, ^3^J_CF_ = 3.5 Hz), 128.9, 128.8, 127.8, 124.7 (q, ^2^J_CF_ = 34 Hz), 123.5 (q, ^1^J_CF_ = 273 Hz), 37.5, 23.2. HRMS (ESI, *m/z*): calcd for C_14_H_12_F_3_NS [M + H]^+^: 284.0715; found: 284.0719.

*3-(Benzylsulfanyl)-2-methyl-5-(trifluoromethyl)pyridine N-oxide* (**5f**), pale-yellow crystals; yield 52%; mp 85–86 °C; ^1^H NMR (300 MHz, CDCl_3_): δ 8.33 (s, 1H), 7.29 (m, 5H), 7.22 (s, 1H), 4.15 (s, 2H, SCH_2_), 2.57 (s, 3H, CH_3_). ^13^C NMR (75 MHz, CDCl_3_): δ 151.8, 137.3, 134.7, 134.1, 128.9, 128.9, 128.1, 126.6 (q, ^2^J_CF_ = 35 Hz), 121.9 (q, ^1^J_CF_ = 273 Hz), 120.1, 38.4, 15.0. HRMS (ESI, *m/z*): calcd for C_14_H_12_F_3_NOS [M + H]^+^: 300.0664; found: 300.0662.

*(E)-3-(Benzylsulfanyl)-2-((4-chlorophenyl)vinyl)-5-nitropyridine* (**5g**), yellow crystals; yield 56%; mp 146–147 °C; ^1^H NMR (300 MHz, CDCl_3_): δ 9.17 (d, 1H, *J* = 2.1 Hz), 8.28 (d, 1H, *J* = 2.1 Hz), 7.92 (d, 1H, *J* = 15.6 Hz), 7.57 (d, 1H, *J* = 15.6 Hz), 7.53 (d, 2H, *J* = 8.7 Hz), 7.39 (d, 2H, *J* = 8.7 Hz), 7.30 (m, 5H), 4.18 (s, 2H, SCH_2_). ^13^C NMR (75 MHz, CDCl_3_): δ 159.0, 142.2, 142.1, 138.1, 135.5, 135.2, 134.5, 132.4, 132.3, 129.2, 129.2, 129.0, 128.9, 128.1, 122.8, 38.7. HRMS (ESI, *m/z*): calcd for C_20_H_15_ClN_2_O_2_S [M + H]^+^: 383.0616; found: 383.0603.

*(E)-5-(Benzylsulfanyl)-2-((4-chlorophenyl)vinyl)-3-nitropyridine* (**6g**), yellow crystals; yield 18%; mp 183–184 °C; ^1^H NMR (300 MHz, CDCl_3_): δ 8.63 (d, 1H, *J* = 1.8 Hz), 8.06 (d, 1H, *J* = 2.1 Hz), 7.97 (d, 1H, *J* = 15.6 Hz), 7.67 (d, 1H, *J* = 15.6 Hz), 7.56 (d, 2H, *J* = 8.7 Hz), 7.39 (d, 2H, *J* = 8.7 Hz), 7.33 (m, 5H), 4.22 (s, 2H, SCH_2_). HRMS (ESI, *m/z*): calcd for C_20_H_15_ClN_2_O_2_S [M + H]^+^: 383.0616; found: 383.0621.

*2-[(E)-(4-Chlorophenyl)vinyl]-3-(isobutylsulfanyl)-5-nitropyridine* (**5h**), yellow crystals; yield 62%; mp 139–140 °C; ^1^H NMR (300 MHz, CDCl_3_): δ 9.16 (d, 1H, *J* = 2.4 Hz), 8.31 (d, 1H, *J* = 2.1 Hz), 7.96 (d, 1H, *J* = 15.3 Hz), 7.63 (d, 1H, *J* = 15.3 Hz), 7.60 (d, 2H, *J* = 8.4 Hz), 7.41 (d, 2H, *J* = 8.4 Hz), 2.92 (d, 2H, *J* = 6.9 Hz), 1.99 (m, 1H), 1.14 (d, 6H, *J* = 6.6 Hz). ^13^C NMR (75 MHz, CDCl_3_): δ 158.0, 147.0, 142.4, 141.0, 137.9, 135.4, 134.5, 134.3, 129.9, 122.8, 42.3, 28.1, 22.2. HRMS (ESI, *m/z*): calcd for C_17_H_17_ClN_2_O_2_S [M + H]^+^: 349.0772; found: 349.0783.

*2-[(E)-(4-Chlorophenyl)vinyl]-5-(isobutylsulfanyl)-3-nitropyridine* (**6h**), yellow crystals; yield 31%; mp 106 °C; ^1^H NMR (300 MHz, CDCl_3_): δ 8.68 (d, 1H, *J* = 1.8 Hz), 8.09 (d, 1H, *J* = 1.8 Hz), 7.97 (d, 1H, *J* = 15.6 Hz), 7.68 (d, 1H, *J* = 15.6 Hz), 7.56 (d, 2H, *J* = 8.4 Hz), 7.38 (d, 2H, *J* = 8.4 Hz), 2.92 (d, 2H, *J* = 6.9 Hz), 1.96 (m, 1H), 1.10 (d, 6H, *J* = 6.6 Hz). ^13^C NMR (75 MHz, CDCl_3_): δ 152.0, 145.4, 143.9, 136.8, 135.1, 134.9, 134.6, 130.9, 129.1, 129.0, 121.5, 42.1, 28.3, 22.0. HRMS (ESI, *m/z*): calcd for C_17_H_17_ClN_2_O_2_S [M + H]^+^: 349.0772; found: 349.0764.

*2-[(E)-2-(4-Chlorophenyl)vinyl]-3-[(2-furylmethyl)sulfanyl]-5-nitropyridine* (**5i**), yellow crystals; yield 56%; mp 156 °C; ^1^H NMR (300 MHz, CDCl_3_): δ 9.24 (d, 1H, *J* = 1.8 Hz), 8.42 (d, 1H, *J* = 2.1 Hz), 7.95 (d, 1H, *J* = 15.6 Hz), 7.65 (d, 1H, *J* = 15.6 Hz), 7.58 (d, 2H, *J* = 8.4 Hz), 7.40 (d, 2H, *J* = 8.4 Hz), 7.33 (s, 1H), 6.26 (s, 1H), 6.14 (d, 1H, *J* = 3.0 Hz), 4.18 (s, 2H). HRMS (ESI, *m/z*): calcd for C_18_H_13_ClN_2_O_2_S [M + H]^+^: 373.0408; found: 373.0419.

*3-[(4-Chlorophenyl)sulfanyl]-2-[(E)-2-(4-chlorophenyl)vinyl]-5-nitropyridine* (**5j**)**,** yellow solid; yield 67%; mp 172 °C; ^1^H NMR (300 MHz, DMSO-d_6_): δ 9.26 (s, 1H), 8.22 (s, 1H), 7.98 (d, 1H, *J* = 15.6 Hz), 7.74 (d, 2H, *J* = 8.4 Hz), 7.66 (d, 1H, *J* = 15.6 Hz), 7.52 (br.s, 4H), 7.49 (d, 2H, *J* = 8.4 Hz). HRMS (ESI, *m/z*): calcd for C_19_H_12_Cl_2_N_2_O_2_S [M + H]^+^: 403.0069; found: 403.0057.

*(E)-4-(2-(3-((4-Chlorophenyl)sulfanyl)-5-nitropyridin-2-yl)vinyl)-N,N-dimethylaniline* (**5k**)**,** dark-red solid; yield 83%; mp 175 °C; ^1^H NMR (300 MHz, DMSO-d_6_): δ 9.21 (s, 1H), 8.20 (s, 1H), 7.96 (d, 1H, *J* = 15.6 Hz), 7.50 (m, 6H), 7.36 (d, 1H, *J* = 15.6 Hz), 6.74 (d, 2H, *J* = 8.4 Hz), 2.99 (s, 6H). ^13^C NMR (125 MHz, CDCl_3_): δ 160.1, 151.6, 143.1, 141.3, 141.2, 134.9, 133.4, 133.2, 130.9, 130.1, 129.9, 123.7, 117.0, 111.9, 40.1. HRMS (ESI, *m/z*): calcd for C_21_H_18_ClN_3_O_2_S [M + H]^+^: 412.0881; found: 412.0870.

*(E)-4-(2-(3-(Isobutylsulfanyl)-5-nitropyridin-2-yl)vinyl)-N,N-dimethylaniline* (**5l**), dark-red solid; yield 84%; mp 165–166 °C; ^1^H NMR (300 MHz, CDCl_3_): δ 9.14 (d, 1H, *J* = 2.4 Hz), 8.27 (d, 1H, *J* = 2.1 Hz), 8.01 (d, 1H, *J* = 15.3 Hz), 7.58 (d, 2H, *J* = 8.7 Hz), 7.46 (d, 1H, *J* = 15.3 Hz), 6.74 (d, 2H, *J* = 8.7 Hz), 3.06 (s, 6H), 2.89 (d, 2H, *J* = 6.6 Hz), 1.96 (m, 1H), 1.13 (d, 6H, *J* = 6.6 Hz). ^13^C NMR (75 MHz, CDCl_3_): δ 159.5, 151.5, 141.4, 141.3, 140.2, 132.5, 130.0, 129.8, 124.1, 117.4, 112.0, 43.0, 40.2, 28.1, 22.2. HRMS (ESI, *m/z*): calcd for C_19_H_23_N_3_O_2_S [M + H]^+^: 358.1584; found: 358.1583.

*(E)-4-(2-(3-(Benzylsulfanyl)-5-nitropyridin-2-yl)vinyl)-N,N-dimethylaniline* (**5m**), dark-red solid; yield 88%; mp 188 °C; ^1^H NMR (300 MHz, CDCl_3_): δ 9.13 (d, 1H, J = 2.1 Hz), 8.20 (d, 1H, *J* = 1.8 Hz), 7.98 (d, 1H, *J* = 15.3 Hz), 7.54 (d, 2H, *J* = 8.7 Hz), 7.43 (d, 1H, *J* = 15.3 Hz), 7.30 (m, 5H), 6.70 (d, 2H, *J* = 8.7 Hz), 4.16 (s, 2H), 3.05 (s, 6H). ^13^C NMR (75 MHz, CDCl_3_): δ 160.4, 151.5, 142.3, 141.0, 140.5, 135.5, 132.2, 130.6, 129.9, 129.0, 128.8, 127.9, 124.0, 117.4, 112.0, 40.2, 38.5. HRMS (ESI, *m/z*): calcd for C_22_H_21_N_3_O_2_S [M + H]^+^: 392.1427; found: 392.1417.

*(E)-3-(Benzylsulfanyl)-2-(2-(1-(4-fluorophenyl)-1H-pyrazol-4-yl)vinyl)-5-nitropyridine* (**5n**)**,** orange solid; yield 89%; mp 145–146 °C; ^1^H NMR (300 MHz, CDCl_3_): δ 9.15 (d, 1H, *J* = 2.1 Hz), 8.26 (d, 1H, *J* = 2.1 Hz), 8.05 (s, 1H), 7.98 (s, 1H), 7.92 (d, 1H, *J* = 15.3 Hz), 7.67 (m, 2H), 7.40 (d, 1H, *J* = 15.3 Hz), 7.30 (m, 5H), 7.18 (m, 2H), 4.18 (s, 2H). ^13^C NMR (75 MHz, CDCl_3_): δ 161.4 (d, ^1^J_CF_ = 247 Hz), 159.3, 142.1, 141.8, 140.3, 136.0 (d, ^4^J_CF_ = 3 Hz), 135.3, 132.2, 131.5, 129.3, 129.0, 128.9, 128.0, 126.4, 121.9, 121.4, 121.1 (d, ^3^J_CF_ = 8.5 Hz), 116.5 (d, ^2^J_CF_ = 23 Hz), 38.6. HRMS (ESI, *m/z*): calcd for C_23_H_17_FN_4_O_2_S [M + H]^+^: 433.1129; found: 433.1123.

*(E)-3-((4-Chlorophenyl)sulfanyl)-2-(2-(1-(4-fluorophenyl)-1H-pyrazol-4-yl)vinyl)-5-nitropyridine* (**5o**), orange solid; yield 93%; mp 219 °C; ^1^H NMR (300 MHz, DMSO-d_6_): δ 9.24 (s, 1H), 8.99 (s, 1H), 8.22 (s, 1H), 8.18 (s, 1H), 7.80 (m, 4H), 7.55 (s, 4H), 7.40 (m, 3H). HRMS (ESI, *m/z*): calcd for C_22_H_14_ClFN_4_O_2_S [M + H]^+^: 453.0583; found: 453.0573.

*(E)-3-(Benzylsulfanyl)-5-bromo-2-[(4-chlorophenyl)vinyl]pyridine* (**5p**), beige solid, yield 60%; mp 142–143 °C; ^1^H NMR (300 MHz, CDCl_3_): δ 8.48 (s, 1H), 7.69–7.73 (m, 2H), 7.48–7.56 (m, 3H), 7.25-7.36 (m, 7H), 4.08 (s, 2H). ^13^C NMR (125 MHz, CDCl_3_): δ 153.2, 148.4, 141.1, 135.9, 135.1, 134.3, 133.6, 132.5, 128.9, 128.8, 128.7, 128.6, 127.7, 123.7, 118.3, 39.4. HRMS (ESI, *m/z*): calcd for C_20_H_15_BrClNS [M + H]^+^: 415.9869; found: 415.9870.

*(E)-3-(Benylsulfanyl)-5-bromo-2-[(4-(dimethylamino)phenyl)vinyl]pyridine N-oxide* (**5q**), dark-red solid; yield 67%; mp 177 °C; ^1^H NMR (300 MHz, CDCl_3_): δ 8.49 (d, 1H, *J* = 15.9 Hz), 8.23 (s, 1H), 7.51 (d, 2H, *J* = 8.7 Hz), 7.28 (m, 7H), 6.71 (d, 2H, *J* = 8.7 Hz), 4.14 (s, 2H), 3.03 (s, 6H, NCH_3_). ^13^C NMR (75 MHz, CDCl_3_): δ 151.2, 144.9, 140.5, 139.0, 135.9, 135.0, 129.2, 129.1, 128.8, 128.4, 127.9, 127.8, 124.8, 114.1, 112.1, 40.3, 39.0. HRMS (ESI, *m/z*): calcd for C_22_H_21_BrN_2_OS [M + H]^+^: 441.0631; found: 441.0635.

### 4.7. X-ray Crystallographic Data and Refinement Details

X-ray diffraction data for **5h** were collected at 100 K on a Bruker Quest D8 diffractometer (Karlsruhe, Germany) equipped with a Photon III area detector, using graphite-monochromatized Mo K_α_-radiation and a shutterless φ- and ω-scan technique. The intensity data were integrated by the SAINT program (version 8.04A) [21] and were semi-empirically corrected for absorption and decay using SADABS (2016/2) [22]. X-ray diffraction data for **4a**, **4i** and **5l** were collected at 100 K on a four-circle Rigaku Synergy-S diffractometer (Wroclaw, Poland) equipped with a HyPix6000HE area detector, using monochromatized Cu K_α_-radiation and a shutterless ω-scan technique. The intensity data were integrated and corrected for absorption and decay by the CrysAlisPro program [23]. All structures were solved by direct methods using SHELXT [24] and refined by the full-matrix least-squares method on *F^2^* using SHELXL-2018 [25]. Positions of all atoms were found from the electron density difference map. Atoms were refined with individual anisotropic (non-hydrogen atoms) or isotropic (hydrogen atoms) displacement parameters. The Mercury program [26] was used for molecular graphics. Crystal data, data collection and structure refinement details are summarized in Supplementary Materials Table S1. The structures were deposited at the Cambridge Crystallographic Data Center with the reference CCDC numbers 2191010–2191013; they also contain the supplementary crystallographic data. These data can be obtained free of charge from the CCDC via http://www.ccdc.cam.ac.uk/data_request/cif, accessed on 30 August 2022.

## Figures and Tables

**Table 1:** Reactions of 2-methyl-3-nitropyridines with thiols.

Substrate	R^1^	n	R^2^	Product, Isolated Yield (%)
**2a**	NO_2_	0	PhCH_2_	**5a**, 70
**3b**	Br	1	PhCH_2_	**5b**, 96
**3b**	Br	1	4-Cl-C_6_H_4_	**5c**, 95
**2b**	Br	0	PhCH_2_	**5d**, 65
**2c**	CF_3_	0	PhCH_2_	**5e**, 60
**3c**	CF_3_	1	PhCH_2_	**5f**, 52

**Table 2:** Reactions of 2-(arylvinyl)-3-nitropyridines with thiols.

Substrate	R^1^	Ar	n	R^2^	Ratio 5/6 ^a^	Product, Isolated Yield (%)
**4a**	NO_2_	4-Cl-C_6_H_4_	0	PhCH_2_	3:1	**5g**, 56 **6g**, 18
**4a**	NO_2_	4-Cl-C_6_H_4_	0	i-C_4_H_9_	2:1	**5h**, 62 **6h**, 31
**4a**	NO_2_	4-Cl-C_6_H_4_	0	2-furylmethyl	>20:1	**5i**, 56
**4a**	NO_2_	4-Cl-C_6_H_4_	0	4-Cl-C_6_H_4_	>20:1	**5j**, 67
**4b**	NO_2_	4-Me_2_N-C_6_H_4_	0	4-Cl-C_6_H_4_	>20:1	**5k**, 83
**4b**	NO_2_	4-Me_2_N-C_6_H_4_	0	i-C_4_H_9_	8:1	**5l**, 84
**4b**	NO_2_	4-Me_2_N-C_6_H_4_	0	PhCH_2_	10:1	**5m**, 88
**4d**	NO_2_	1-(4-fluorophenyl)pyrazol-4-yl	0	PhCH_2_	10:1	**5n**, 89
**4d**	NO_2_	1-(4-fluorophenyl)pyrazol-4-yl	0	4-Cl-C_6_H_4_	>20:1	**5o**, 93
**4j**	Br	4-Cl-C_6_H_4_	0	PhCH_2_	>20:1	**5p**, 60
**4f**	Br	4-Me_2_N-C_6_H_4_	1	PhCH_2_	>20:1	**5q**, 67

^a^ Determined from ^1^H NMR spectrum of crude product.

**Table 3:** Photophysical properties of some synthesized compounds.

Compound	λ_max_ nm	ε_1_ dm^3^ mol^−1^ cm^−1^	λ_max_ nm	ε_2_ dm^3^ mol^−1^ cm^−1^
**4a**	-	-	368	46,400
**4b**	302	13,500	509	42,800
**4c**	263	16,200	397	34,900
**4e**	267	28,700	332	24,700
**4i**	-	-	326	39,000
**4k**	266	14,600	429	32,800
**5g**	296	16,000	384	20,300
**5m**	305	13,600	476	31,400
**5n**	266	18,100	403	25,900
**5p**	302	25,200	344	19,000
**6g**	267	15,000	337	31,400

## Data Availability

Not applicable.

## Supplementary Materials

The following are available online at https://www.mdpi.com/article/10.3390/molecules27175692/s1, X-ray data, ^1^H and ^13^C NMR spectra. References [19,20,21,22,23,24,25,26] are cited in the supplementary materials.

## Sample Availability

Samples of the compounds are not available from the authors.
