# Supplementary material for: Nucleophilic Functionalization of 2-R-3-Nitropyridines as a Versatile Approach to Novel Fluorescent Molecules
Source: Molecules. 2022 Sep 3;27(17):5692. doi: 10.3390/molecules27175692 (PMC9457606; doi:10.3390/molecules27175692)

Supplementary materials.

Nucleophilic functionalization of 2-R-3-nitropyridines as a versatile approach to novel fluorescent molecules

Vladislav V. Nikol’skiy,[a] Mikhail E. Minyaev,[a] Maxim A. Bastrakov[a] and Alexey M. Starosotnikov*[a]

N.D. Zelinsky Institute of Organic Chemistry RAS, Leninsky prosp. 47, 11991 Moscow, Russia
E-mail: alexey41@list.ru

X-ray crystallographic data and refinement details.

X-ray diffraction data for **5h** were collected at 100K on a Bruker Quest D8 diffractometer equipped with a Photon-III area-detector, using graphite-monochromatized Mo K_{α}-radiation and shutterless φ- and ω-scan technique. The intensity data were integrated by the SAINT program[S3] and were semi-empirically corrected for absorption and decay using SADABS.[S4] X-ray diffraction data for **4a**, **4i** and **5l** were collected at 100K on a four-circle Rigaku Synergy S diffractometer equipped with a HyPix6000HE area-detector, using monochromatized Cu K_{α}-radiation and shutterless ω-scan technique. The intensity data were integrated and corrected for absorption and decay by the CrysAlisPro program.[S5] All structures were solved by direct methods using SHELXT[S6] and refined by the full-matrix least-squares method on F^2 using SHELXL-2018.[S7] Positions of all atoms were found from the electron density-difference map. Atoms were refined with individual anisotropic (non-hydrogen atoms) or isotropic (hydrogen atoms) displacement parameters. The *Mercury* program[S8] was used for molecular graphics.

Crystal data, data collection and structure refinement details are summarized in Table S1. The structures have been deposited at the Cambridge Crystallographic Data Center with the reference CCDC numbers 2191010-2191013; they also contain the supplementary crystallographic data. These data can be obtained free of charge from the CCDC *via* http://www.ccdc.cam.ac.uk/data_request/cif

Table S1. Crystal data, data collection and structure refinement details for **4a**, **4i**, **5h** and **5l**.

Identification code	**4a**	**4i**	**5h**	**5l**
Empirical formula	$C_{13}H_8ClN_3O_4$	$C_{14}H_8ClF_3N_2O_2$	$C_{17}H_{17}ClN_2O_2S$	$C_{19}H_{23}N_3O_2S$
Formula weight	305.67	328.67	348.83	357.46
Temperature (K)	100.0(1)	100.0(1)	100(2)	100.0(1)
Wavelength (Å)	1.54184	1.54184	0.71073	1.54184
Crystal system	Triclinic	Triclinic	Monoclinic	Monoclinic
Space group	$P\bar{1}$	$P\bar{1}$	$P2_1/c$	$P2_1/c$
Unit cell dimensions				
a (Å)	7.44202(12)	4.86262(11)	9.5251(2)	9.77416(6)
b (Å)	12.07532(18)	10.0223(2)	17.2271(3)	17.15004(10)
c (Å)	14.4433(3)	13.5534(3)	10.0200(2)	10.58888(5)
α (°)	88.6177(14)	80.0002(18)	90	90
β (°)	77.0110(15)	89.1992(18)	92.1044(6)	96.7058(5)
γ (°)	83.0977(13)	89.4931(19)	90	90
Volume ($Å^3$)	1255.56(4)	650.41(2)	1643.07(6)	1762.842(17)
Z	4	2	4	4
Calcd Density ($g{\bullet}cm^{-3}$)	1.617	1.678	1.410	1.347

μ (mm^{-1})	2.915	3.060	0.370	1.774
F(000)	624	332	728	760
Crystal size (mm)	0.19×0.07×0.05	0.20×0.09×0.04	0.32×0.16×0.06	0.31×0.19×0.10
θ range (°)	3.140-79.821	3.311-79.741	2.140-33.150	4.555-79.463
Indexranges	$-8\leq h\leq 9$, $-15\leq k\leq 15$, $-18\leq l\leq 18$	$-6\leq h\leq 4$, $-12\leq k\leq 12$, $-17\leq l\leq 17$	$-14\leq h\leq 14$, $-26\leq k\leq 26$, $-15\leq l\leq 14$	$-12\leq h\leq 12$, $-21\leq k\leq 21$, $-13\leq l\leq 12$
Reflections				
Collected	31671	16837	47789	45369
Independent [R_{int}]	5409 [0.0314]	2786 [0.0284]	6266 [0.0353]	3828 [0.0263]
Observed ($I>2\sigma(I)$)	5313	2756	5407	3818
Completeness to θ_{full}	0.999	1.000	0.998	1.000
T_{max} / T_{min}	1.0000 / 0.7049	1.0000 / 0.4436	0.6957 / 0.6432	1.0000 / 0.7325
Data/restraints/parameters	5409 / 0 / 443	2786 / 0 / 231	6266 / 0 / 276	3828 / 0 / 319
Goodness-of-fit on F^2	1.079	1.075	1.038	1.035
R1/wR2 indices ($I>2\sigma(I)$)	0.0351 / 0.1012	0.0403 / 0.1148	0.0304 / 0.0781	0.0302 / 0.0843
R1/wR2 indices (all data)	0.0355 / 0.1015	0.0405 / 0.1150	0.0388 / 0.0849	0.0302 / 0.0843
$\Delta\rho(\bar{e})$ max / min ($\bar{e}\cdot Å^{-3}$)	0.282 / -0.386	0.541 / -0.406	0.545 / -0.270	0.430 / -0.249
CCDC number	2191010	2191011	2191012	2191013

The structure of 4a

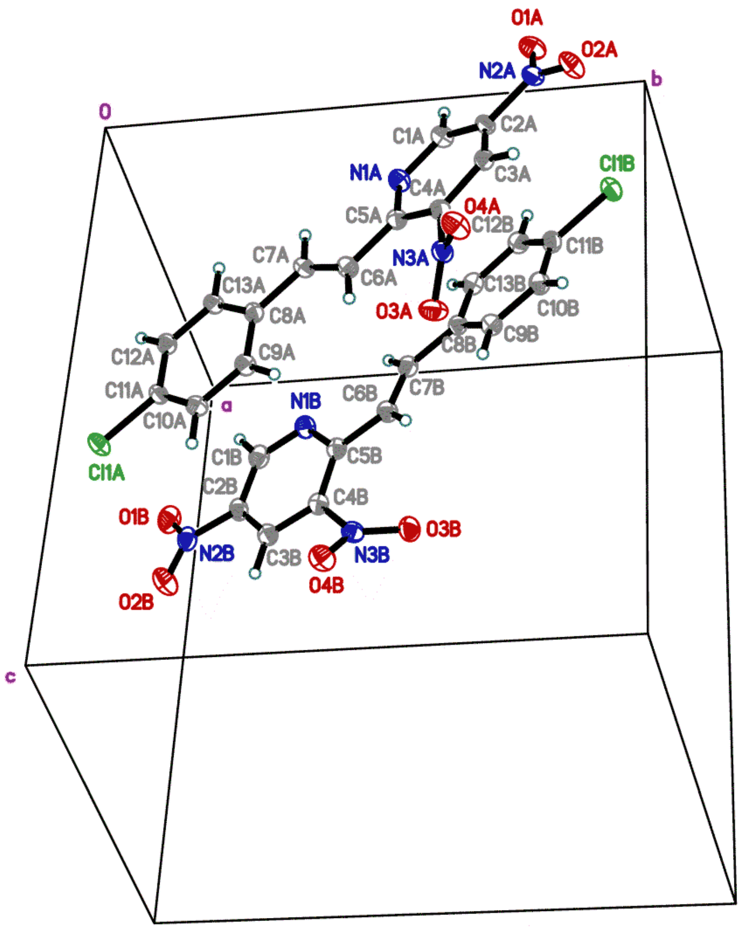

Figure S1. Two crystallographically non-equivalent molecules of **4a** and the unit cell.

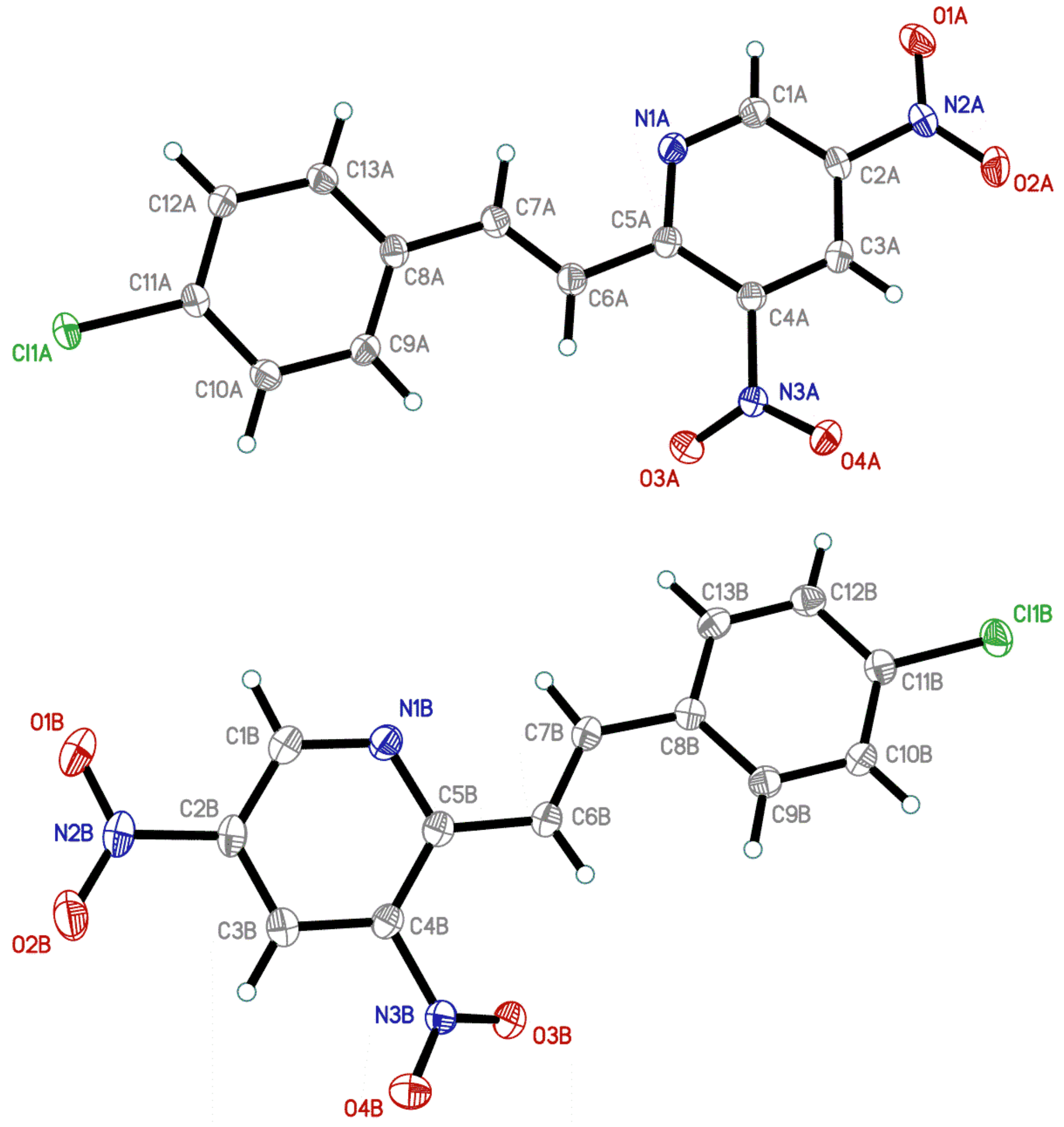

Figure S2. Two non-equivalent molecules of **4a**. Thermal ellipsoids are drawn at the 50% probability level.

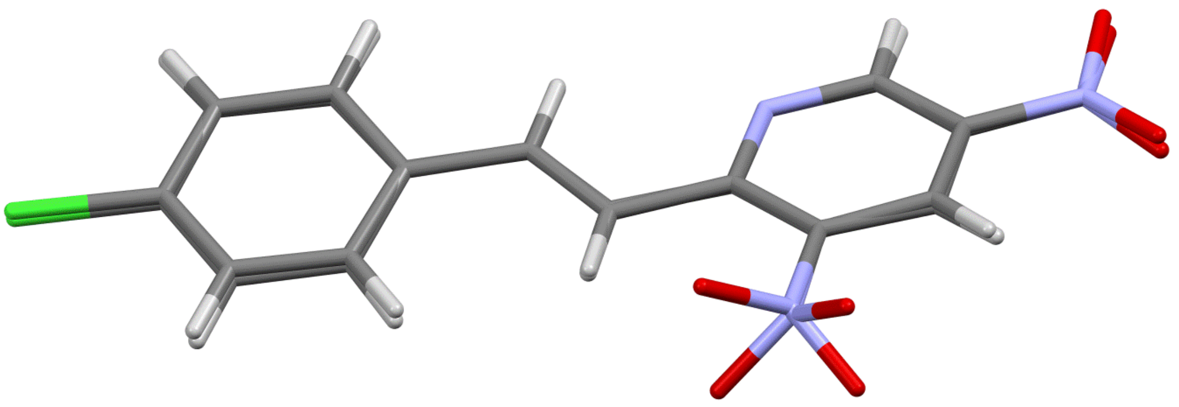

Figure S3. Overlay of two non-equivalent molecules of **4a**.

Table S2. Bond lengths (Å) in **4a**.

Cl1A-C11A	1.7432(12)	C8A-C9A	1.4045(18)	C1B-C2B	1.395(2)
O1A-N2A	1.2278(15)	C8A-C13A	1.4005(17)	C2B-C3B	1.3791(19)
O2A-N2A	1.2279(16)	C9A-H9A	0.975(18)	C3B-H3B	0.968(19)
O3A-N3A	1.2271(15)	C9A-C10A	1.3877(18)	C3B-C4B	1.3837(18)
O4A-N3A	1.2239(15)	C10A-H10A	0.963(19)	C4B-C5B	1.4064(18)
N1A-C1A	1.3229(17)	C10A-C11A	1.3927(18)	C5B-C6B	1.4635(17)
N1A-C5A	1.3574(16)	C11A-C12A	1.3886(17)	C6B-H6B	0.910(19)
N2A-C2A	1.4613(16)	C12A-H12A	0.958(19)	C6B-C7B	1.3438(19)
N3A-C4A	1.4703(15)	C12A-C13A	1.3851(18)	C7B-H7B	0.95(2)
C1A-H1A	0.958(19)	C13A-H13A	0.974(19)	C7B-C8B	1.4625(18)
C1A-C2A	1.3958(18)	Cl1B-C11B	1.7392(13)	C8B-C9B	1.4042(18)
C2A-C3A	1.3718(18)	O1B-N2B	1.2289(16)	C8B-C13B	1.4026(18)
C3A-H3A	0.928(18)	O2B-N2B	1.2252(17)	C9B-H9B	0.979(19)
C3A-C4A	1.3852(17)	O3B-N3B	1.2267(15)	C9B-C10B	1.3872(18)
C4A-C5A	1.4103(17)	O4B-N3B	1.2262(15)	C10B-H10B	0.922(18)
C5A-C6A	1.4611(17)	N1B-C1B	1.3250(18)	C10B-C11B	1.3947(18)
C6A-H6A	0.941(18)	N1B-C5B	1.3555(17)	C11B-C12B	1.3903(19)
C6A-C7A	1.3402(18)	N2B-C2B	1.4601(16)	C12B-H12B	0.933(19)
C7A-H7A	0.965(19)	N3B-C4B	1.4710(16)	C12B-C13B	1.3847(19)
C7A-C8A	1.4660(17)	C1B-H1B	0.981(19)	C13B-H13B	0.942(18)

The structure of 4i

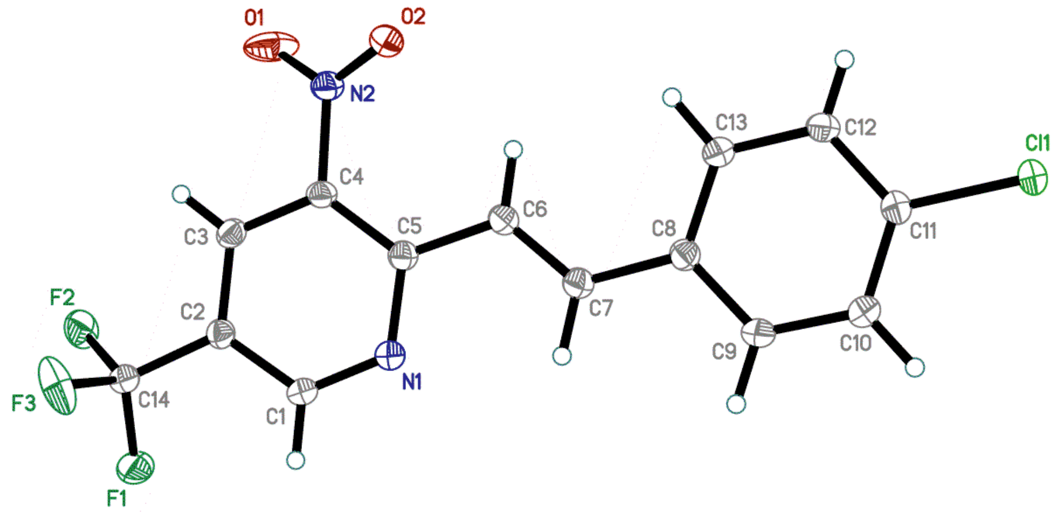

Figure S4. The structure of **4i**. Thermal ellipsoids are drawn at the 50% probability level.

Table S3. Bond lengths (Å) in **4i**.

Cl1-C11	1.7418(16)	C2-C3	1.381(2)	C8-C9	1.398(2)
F1-C14	1.3333(18)	C2-C14	1.493(2)	C8-C13	1.407(2)
F2-C14	1.3358(18)	C3-H3	0.90(3)	C9-H9	0.97(3)
F3-C14	1.3346(19)	C3-C4	1.384(2)	C9-C10	1.384(2)
O1-N2	1.220(2)	C4-C5	1.414(2)	C10-H10	0.96(2)
O2-N2	1.219(2)	C5-C6	1.461(2)	C10-C11	1.391(2)

N1-C1	1.323(2)	C6-H6	0.95(2)	C11-C12	1.393(2)
N1-C5	1.358(2)	C6-C7	1.343(2)	C12-H12	0.91(2)
N2-C4	1.4772(19)	C7-H7	0.98(2)	C12-C13	1.384(2)
C1-H1	0.94(2)	C7-C8	1.465(2)	C13-H13	0.95(2)
C1-C2	1.399(2)				

The structure of 5h

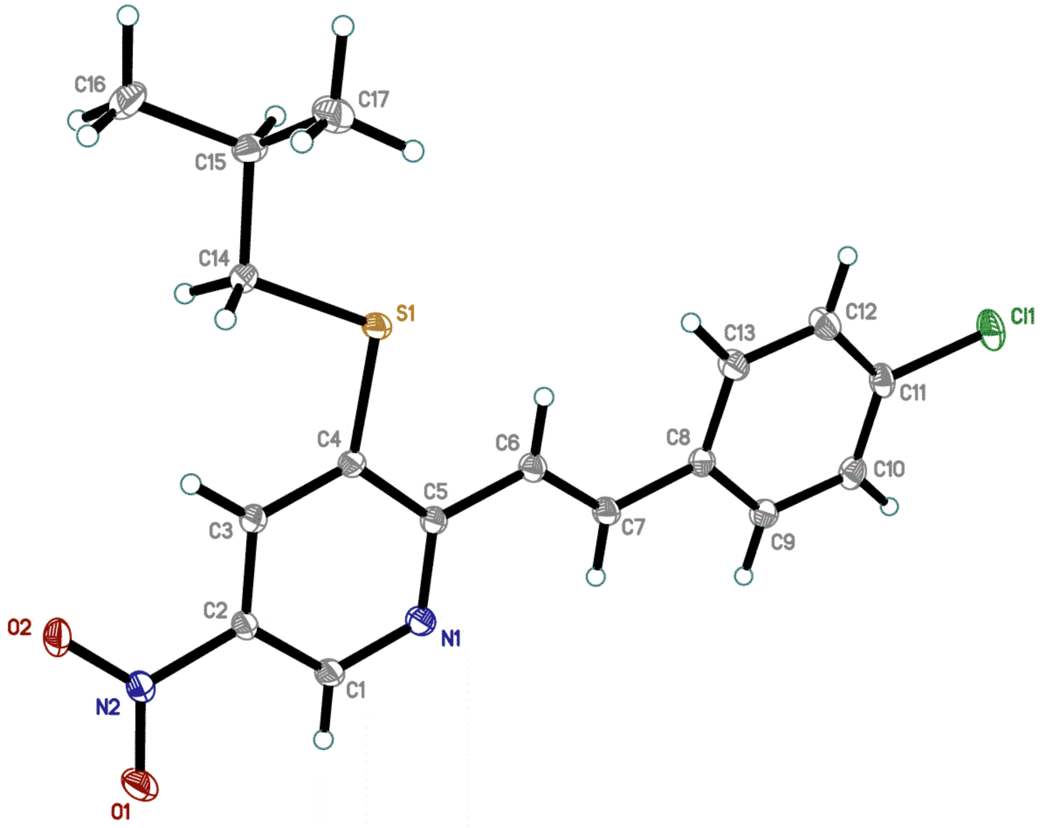

Figure S5. The structure of **5h**. Thermal ellipsoids are drawn at the 50% probability level.

Table S4. Bond lengths (Å) in **5h**.

Cl1-C11	1.7395(9)	C5-C6	1.4609(12)	C13-H13	0.959(16)
S1-C4	1.7530(9)	C6-C7	1.3446(12)	C14-C15	1.5336(13)
S1-C14	1.8093(9)	C6-H6	0.953(14)	C14-H14A	0.990(13)
O1-N2	1.2302(11)	C7-C8	1.4647(12)	C14-H14B	1.008(14)
O2-N2	1.2259(10)	C7-H7	0.929(15)	C15-C17	1.5234(15)
N1-C1	1.3338(12)	C8-C9	1.4020(12)	C15-C16	1.5241(15)
N1-C5	1.3464(11)	C8-C13	1.4025(13)	C15-H15	0.985(15)
N2-C2	1.4606(11)	C9-C10	1.3934(13)	C16-H16A	0.969(18)
C1-C2	1.3884(12)	C9-H9	0.946(14)	C16-H16B	0.945(19)
C1-H1	0.909(15)	C10-C11	1.3833(14)	C16-H16C	1.07(2)
C2-C3	1.3834(12)	C10-H10	0.948(15)	C17-H17A	0.991(16)
C3-C4	1.3937(12)	C11-C12	1.3910(13)	C17-H17B	0.967(19)
C3-H3	0.961(14)	C12-H12	0.956(16)	C17-H17C	1.000(17)

C4-C5	1.4240(12)	C12-C13	1.3858(13)	

The structure of 5l

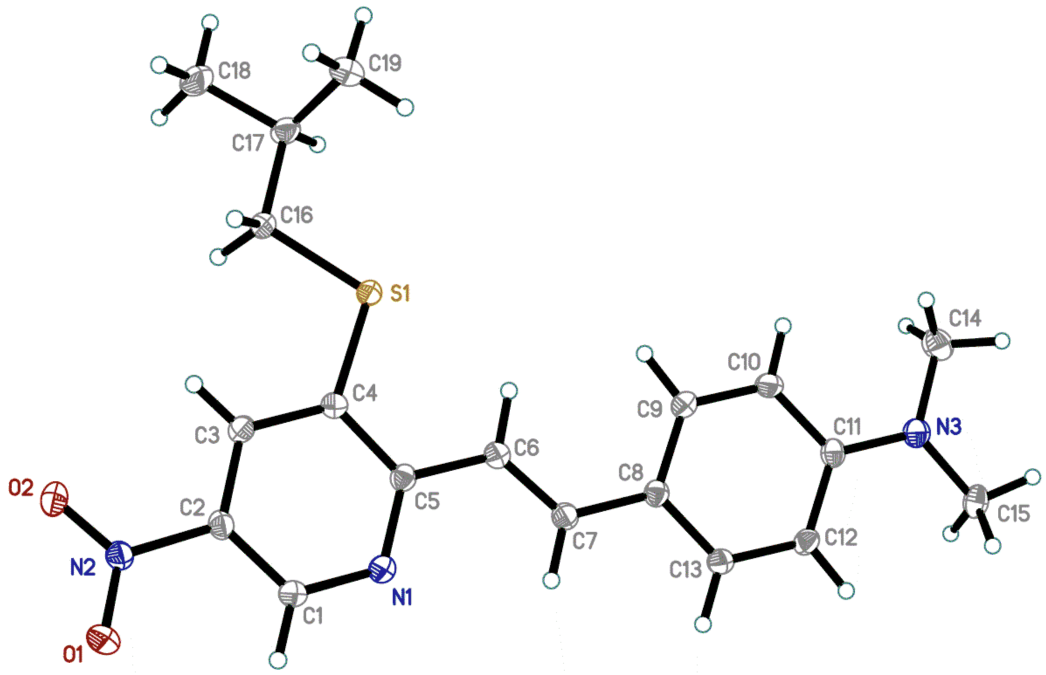

Figure S6. The structure of **5l**. Thermal ellipsoids are drawn at the 50% probability level.

Table S5. Bond lengths (Å) in **5l**.

S1-C4	1.7579(11)	C6-H6	0.963(15)	C15-H15A	0.99(2)
S1-C16	1.8147(10)	C6-C7	1.3493(15)	C15-H15B	1.009(18)
O1-N2	1.2306(12)	C7-H7	0.953(15)	C15-H15C	0.958(18)
O2-N2	1.2276(12)	C7-C8	1.4525(14)	C16-H16A	1.000(15)
N1-C1	1.3347(14)	C8-C9	1.4047(15)	C16-H16B	1.013(14)
N1-C5	1.3520(14)	C8-C13	1.4073(14)	C16-C17	1.5348(14)
N2-C2	1.4577(13)	C9-H9	0.968(15)	C17-H17	0.975(15)
N3-C11	1.3711(13)	C9-C10	1.3777(15)	C17-C18	1.5258(16)
N3-C14	1.4476(15)	C10-H10	0.950(16)	C17-C19	1.5277(17)
N3-C15	1.4527(14)	C10-C11	1.4153(15)	C18-H18A	0.998(18)
C1-H1	0.940(15)	C11-C12	1.4109(15)	C18-H18B	0.974(18)
C1-C2	1.3882(14)	C12-H12	0.965(15)	C18-H18C	0.990(17)
C2-C3	1.3878(15)	C12-C13	1.3862(15)	C19-H19A	0.975(18)
C3-H3	0.917(15)	C13-H13	0.962(15)	C19-H19B	0.977(18)
C3-C4	1.3877(14)	C14-H14A	0.991(19)	C19-H19C	1.008(18)
C4-C5	1.4318(14)	C14-H14B	0.964(19)		
C5-C6	1.4511(14)	C14-H14C	0.994(16)		

References

S1. M.-C. Liu, T.-S. Lin, A. C. Sartorelli, *Synth. Commun.*, 1990, **20**, 2965.

S2. C. D. Jones, R. William, A. Luke, W. McCoull, US Pat. 2008/0194552A1, 2008.

S3. Bruker. APEX-III. *Bruker AXS Inc.*, Madison, Wisconsin, USA, 2019.

S4. L. Krause, R. Herbst-Irmer, G. M. Sheldrick, D. Stalke, *J. Appl. Cryst.,* 2015, **48**, 3.

S5. CrysAlisPro. Version 1.171.41.106a. *Rigaku Oxford Diffraction*, 2021.

S6. G. M. Sheldrick, *Acta Cryst.,* 2015, **A71**, 3.

S7. G. M. Sheldrick, *Acta Cryst.,* 2015, **C71**, 3.

S8. C. F. Macrae, I. Sovago, S. J. Cottrell, P. T. A. Galek, P. McCabe, E. Pidcock, M. Platings, G. P. Shields, J. S. Stevens, M. Towler, P. A. Wood, Mercury 4.0: from visualization to analysis, design and prediction. *J. Appl. Cryst.,* 2020, **53**, 226.

NMR spectra of synthesized compounds.

^{1}H and ^{13}C NMR spectra of compound **3b** in $CDCl_3$

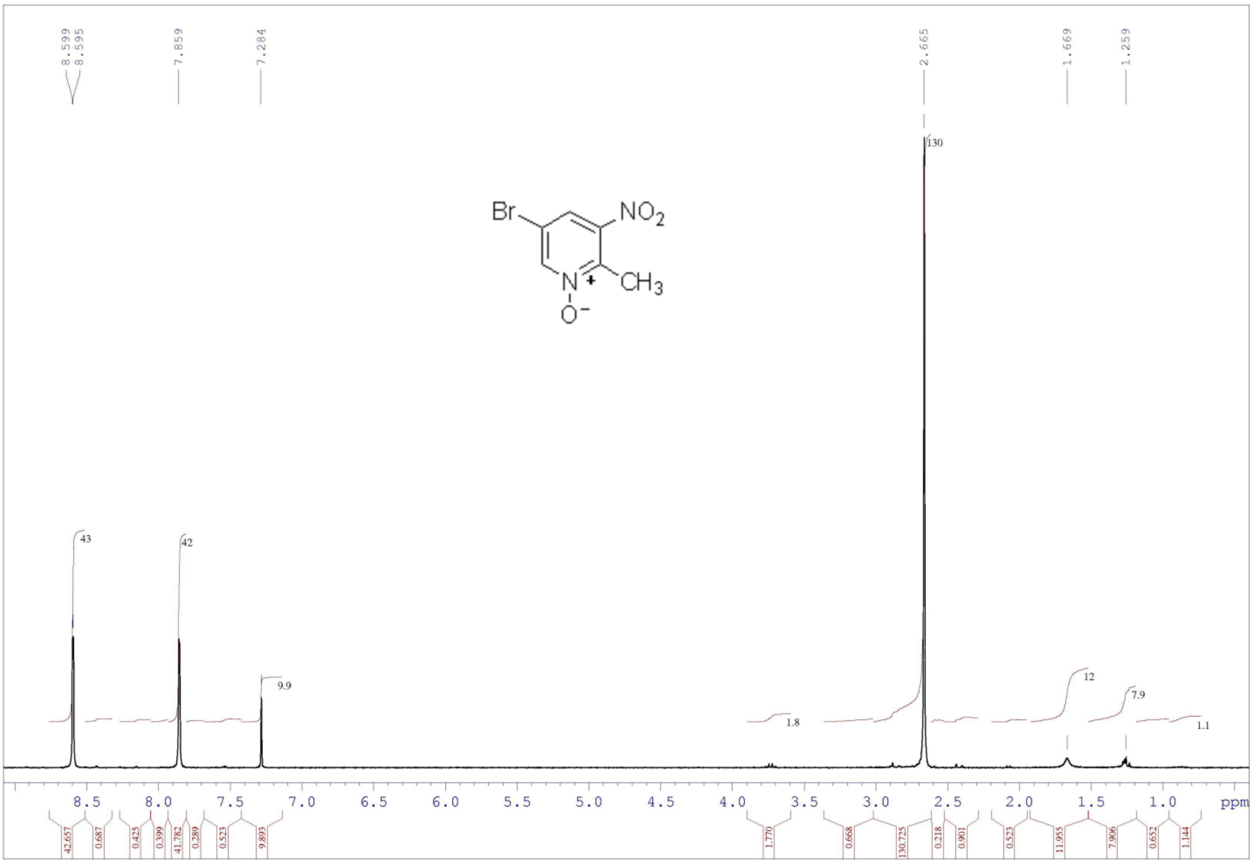

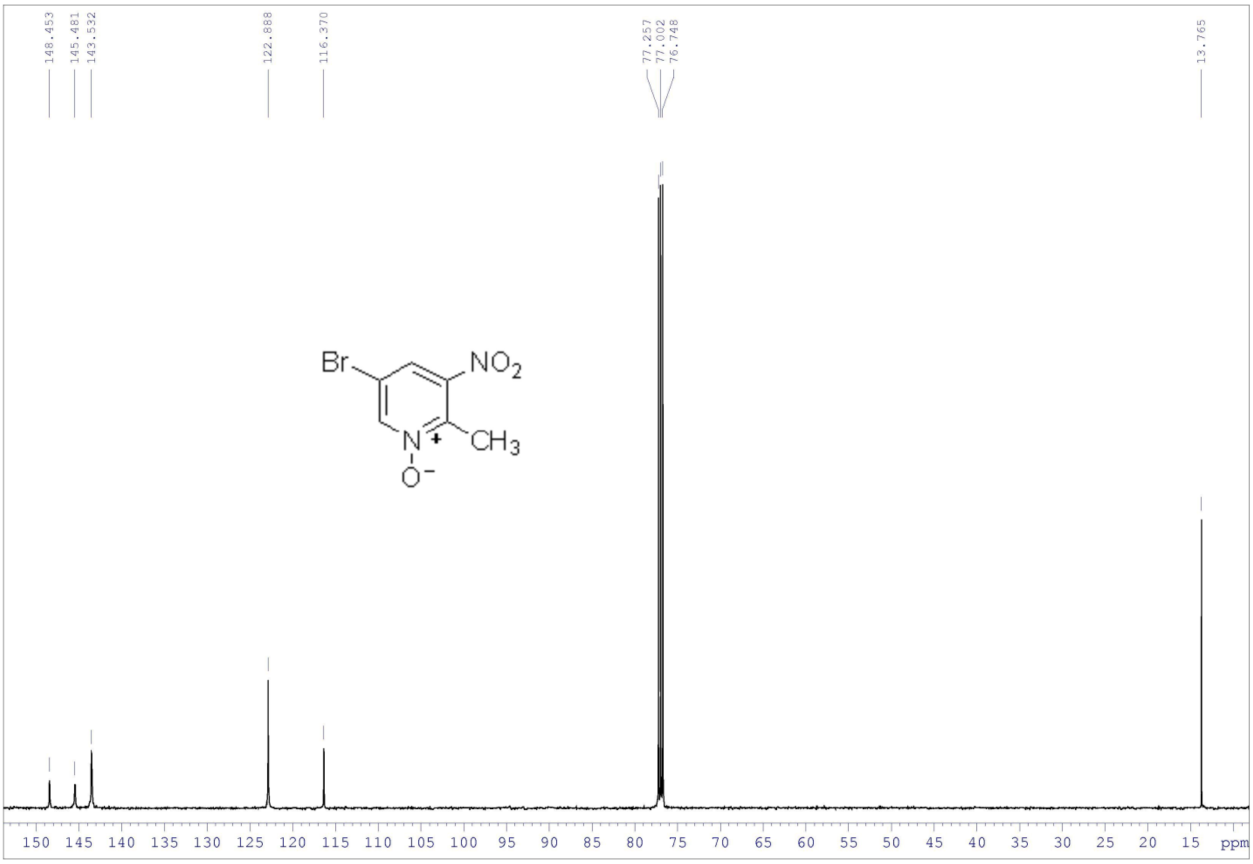

^{1}H and ^{13}C NMR spectra of compound **3c** in $CDCl_3$

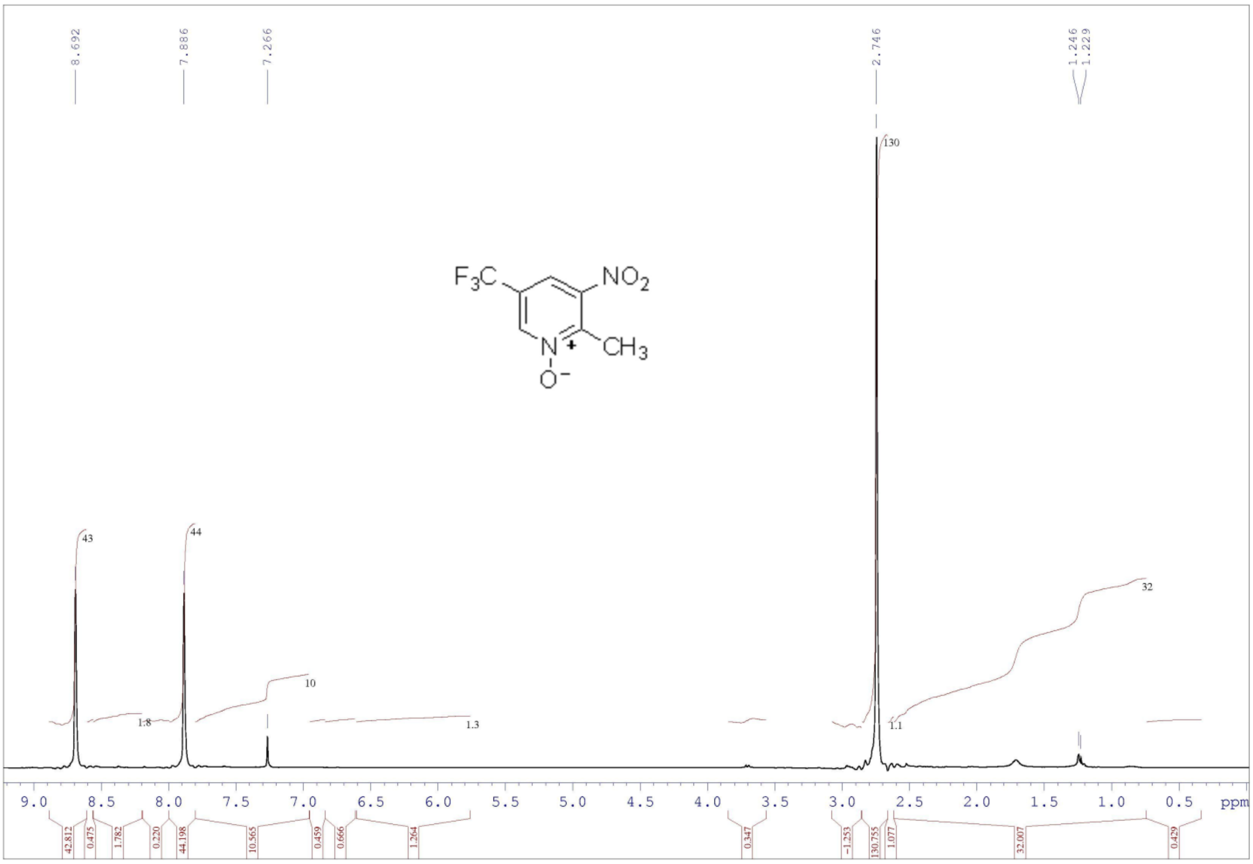

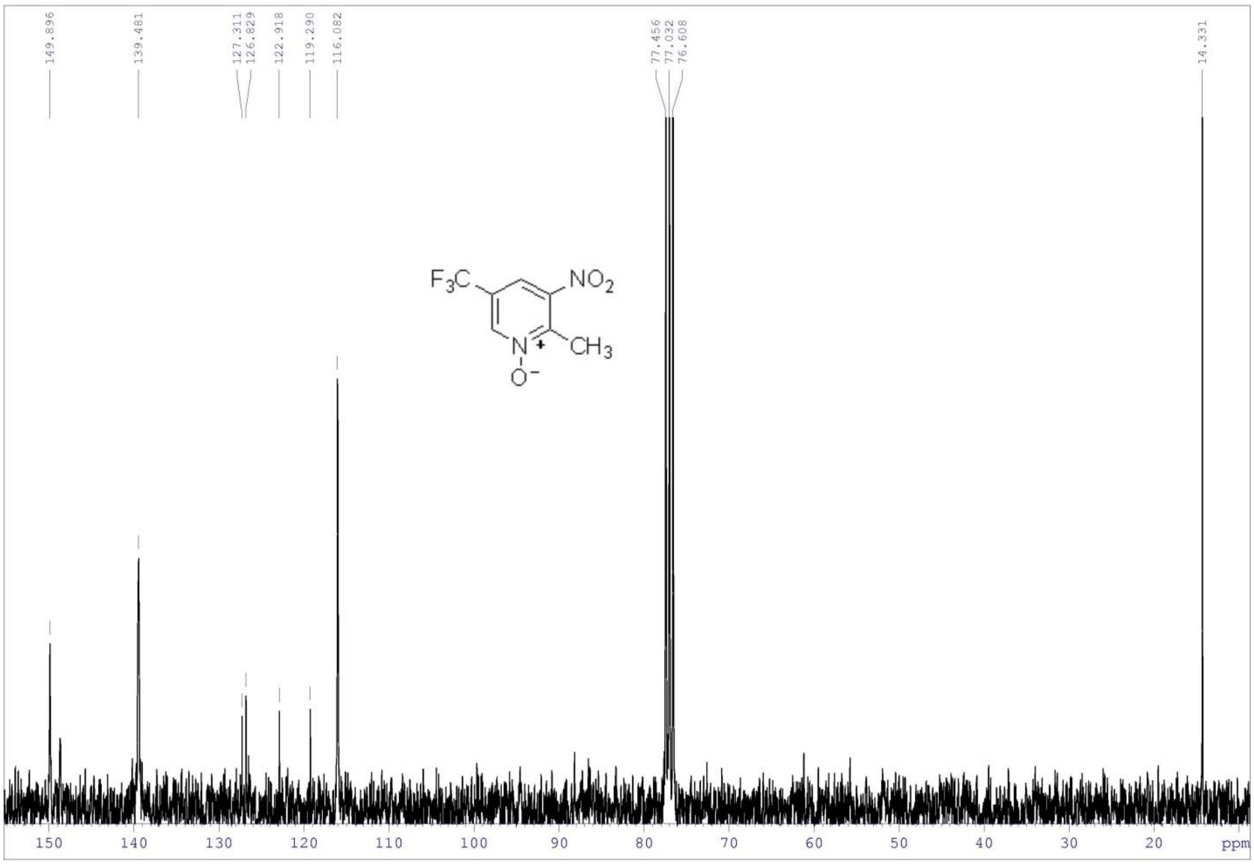

^{1}H NMR spectrum of compound **4a** in $CDCl_3$

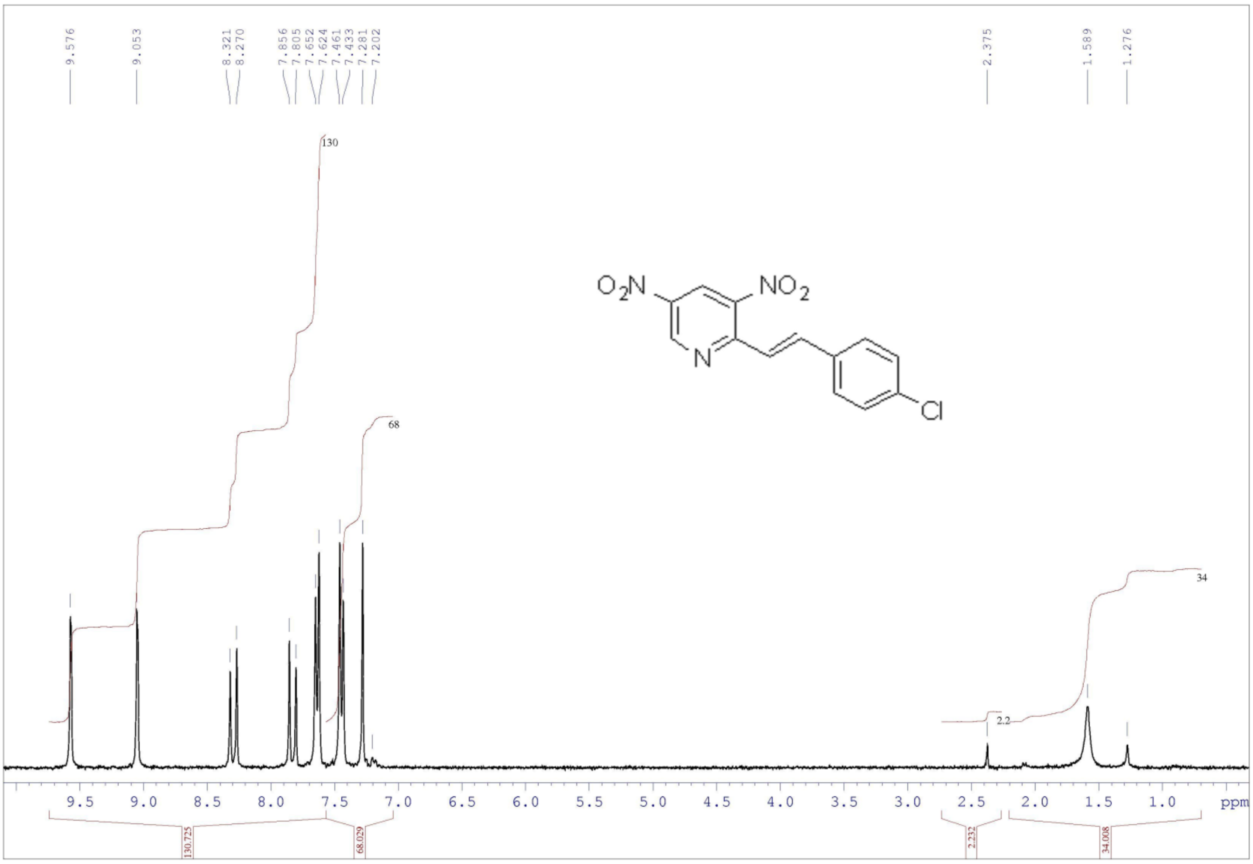

^{13}C NMR spectrum of compound **4a** in DMSO-d_6

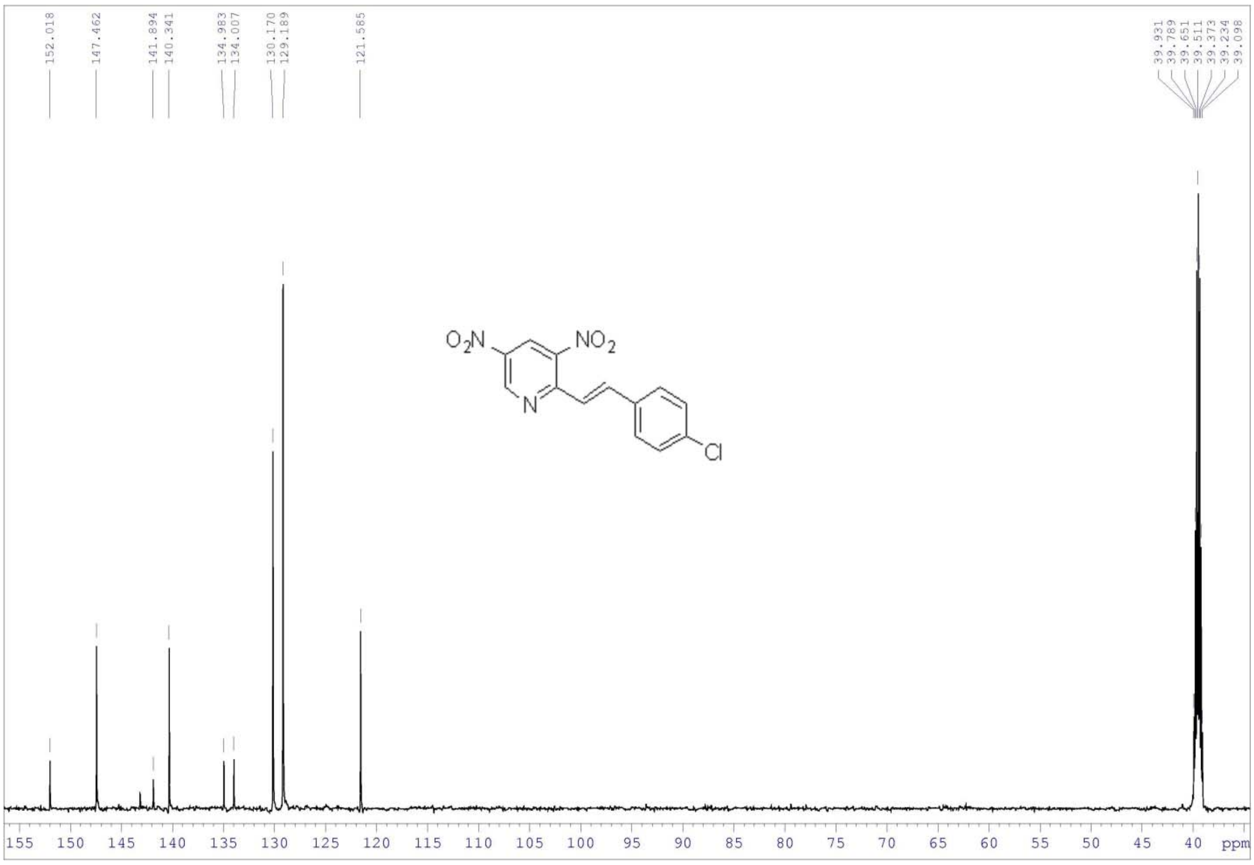

^{1}H and ^{13}C NMR spectra of compound **4b** in $CDCl_3$

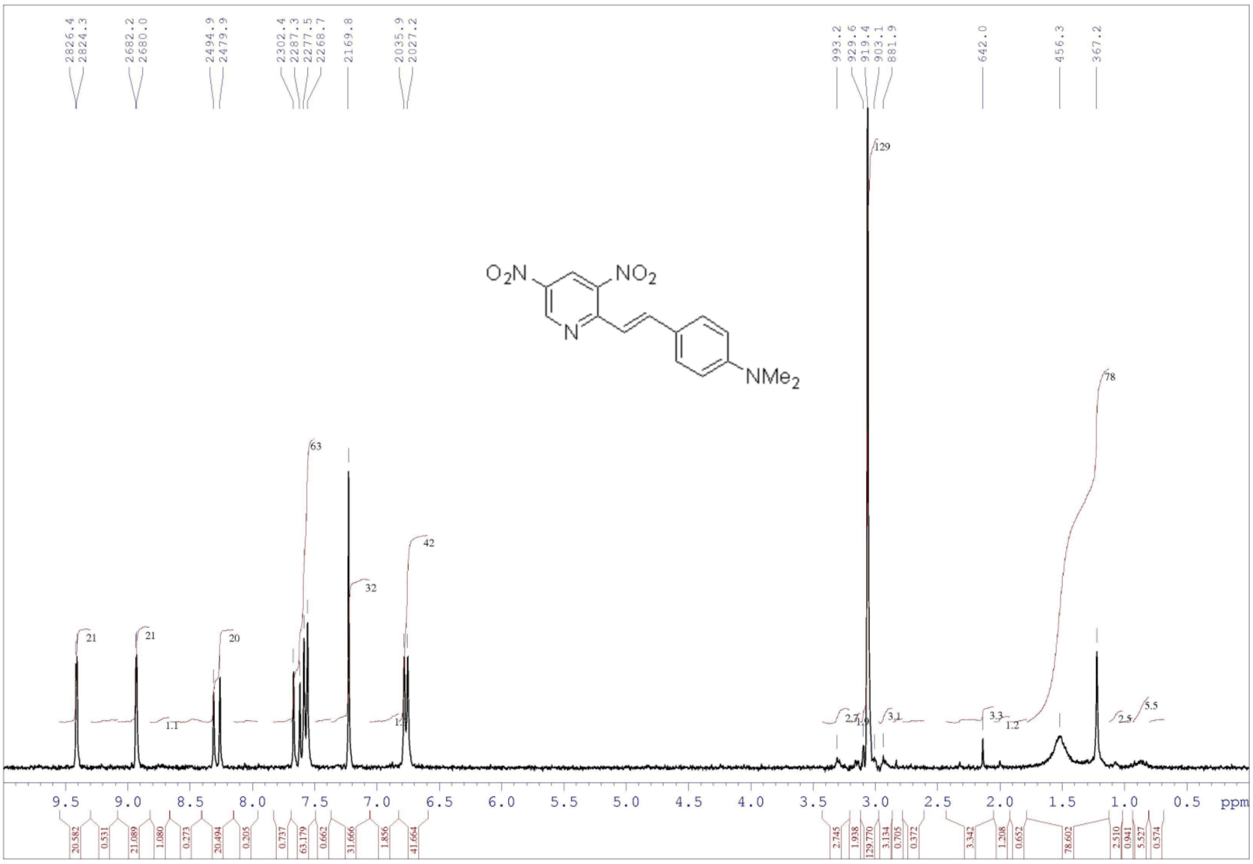

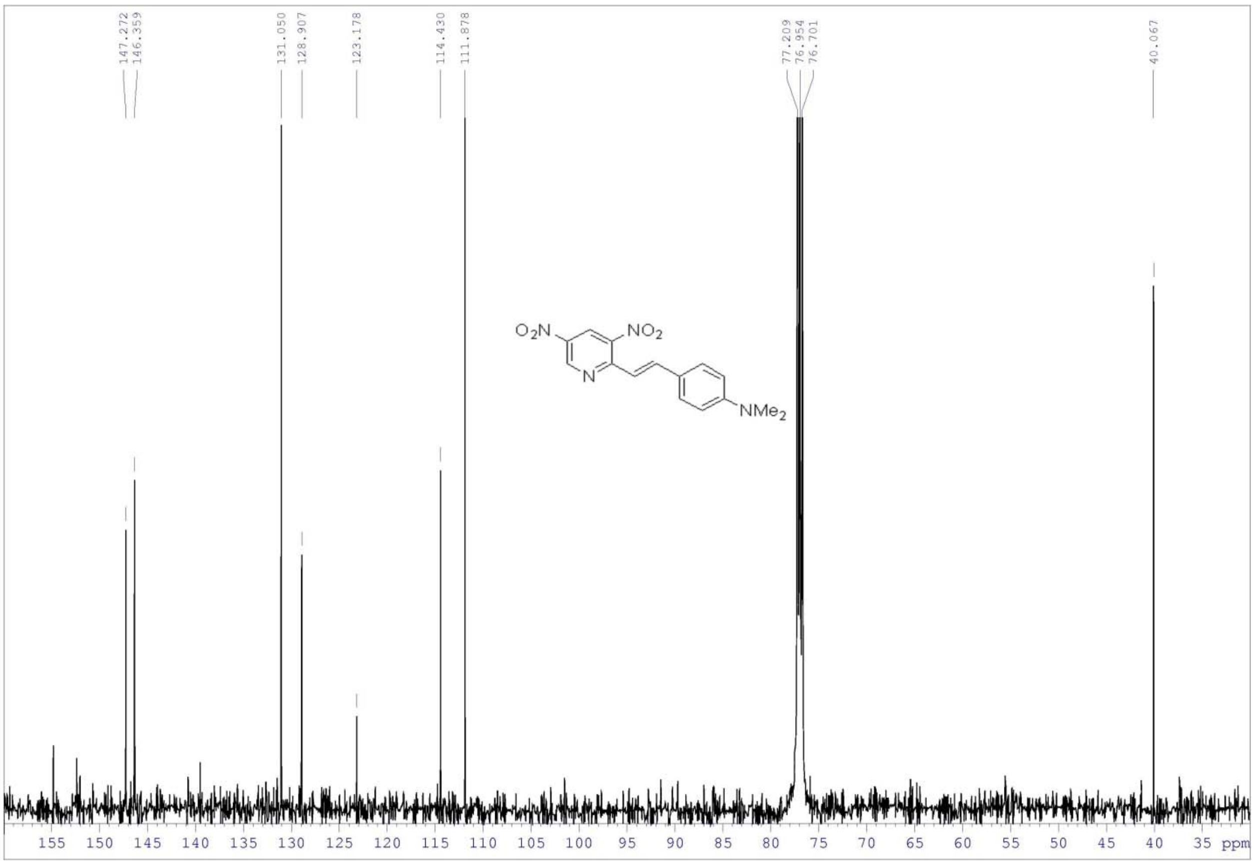

^{1}H and ^{13}C NMR spectra of compound **4c** in $CDCl_3$

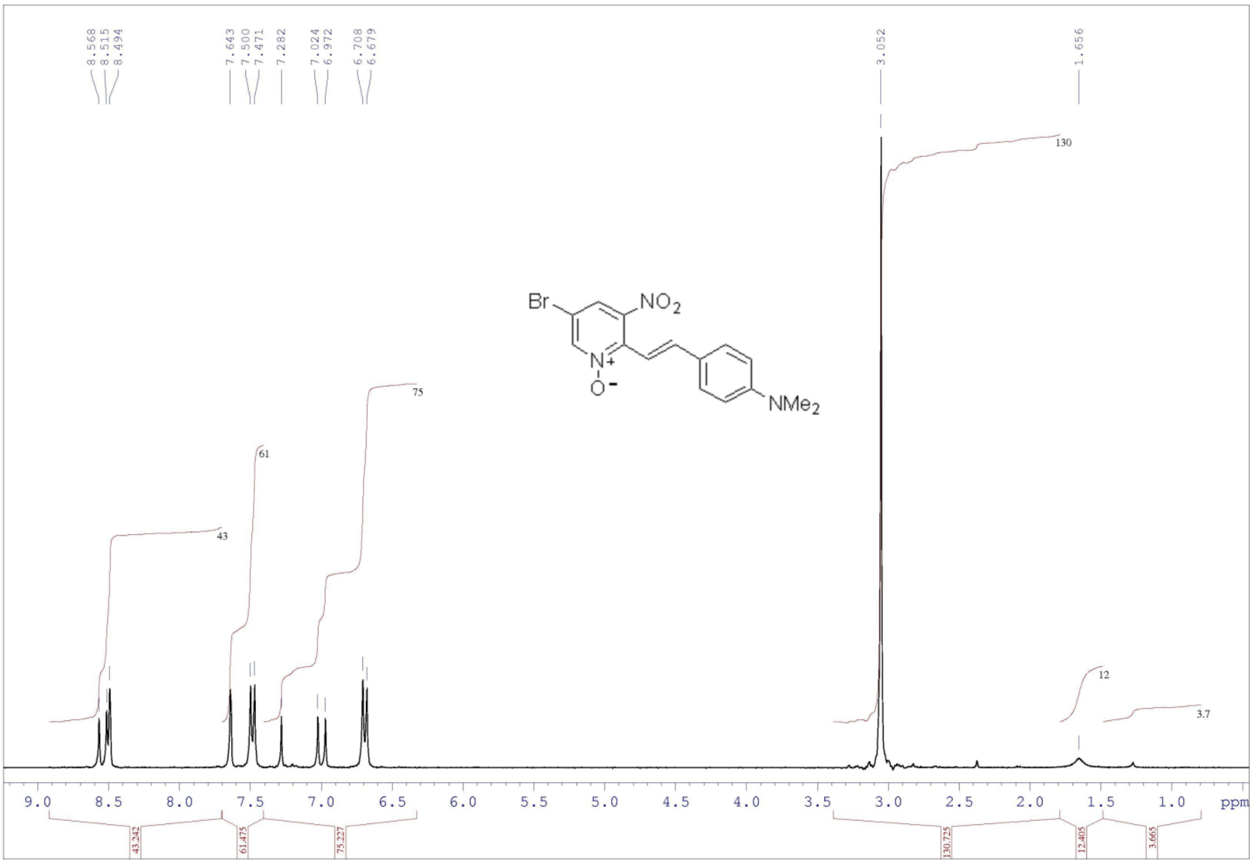

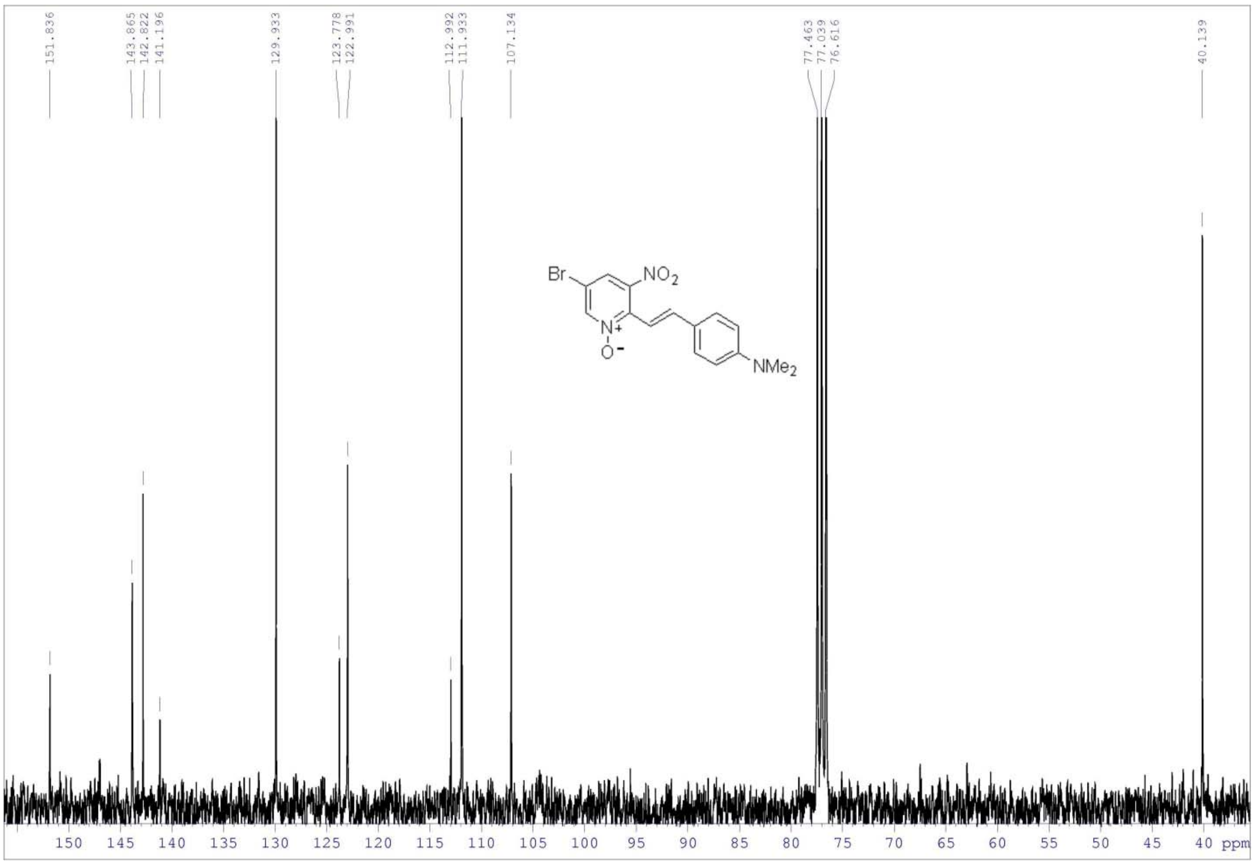

^{1}H NMR spectrum of compound **4d** in $CDCl_3$

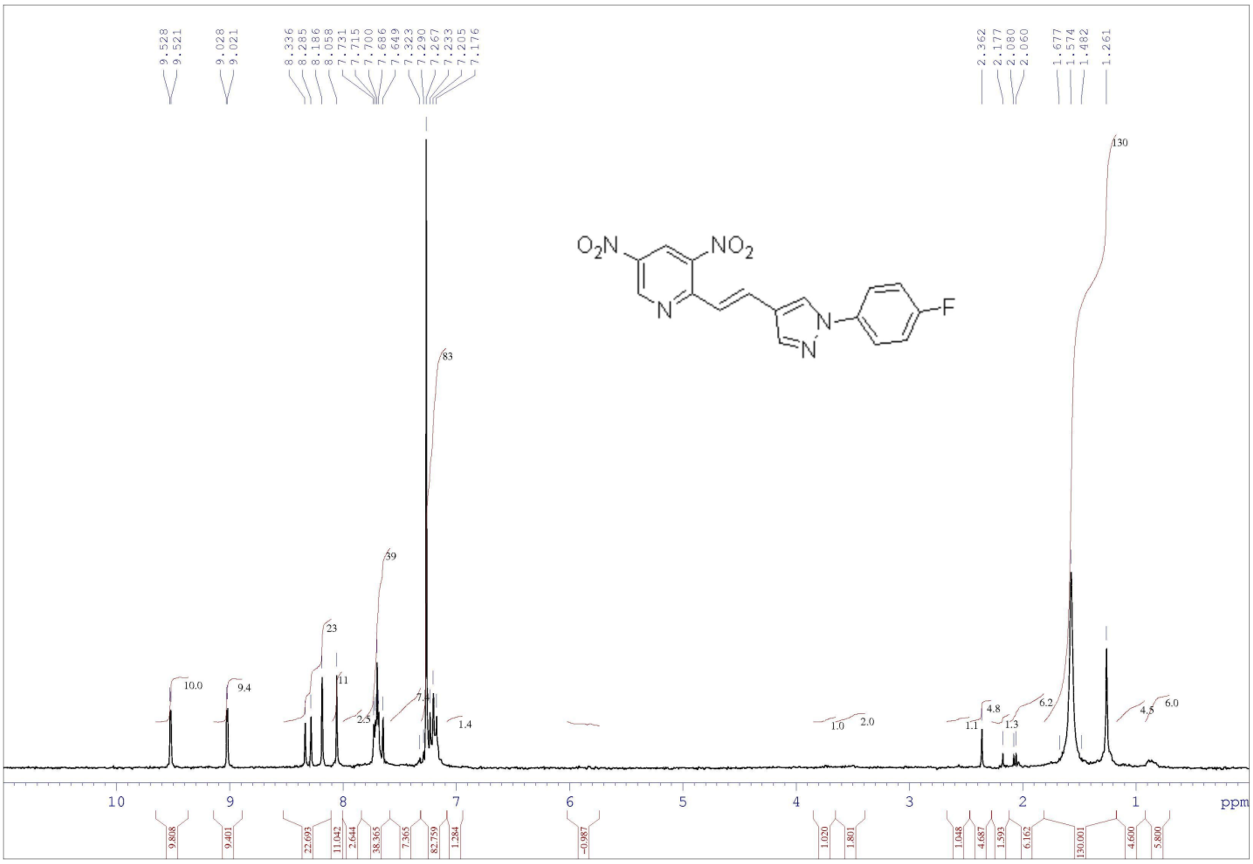

^{1}H and ^{13}C NMR spectra of compound **4e** in $CDCl_3$

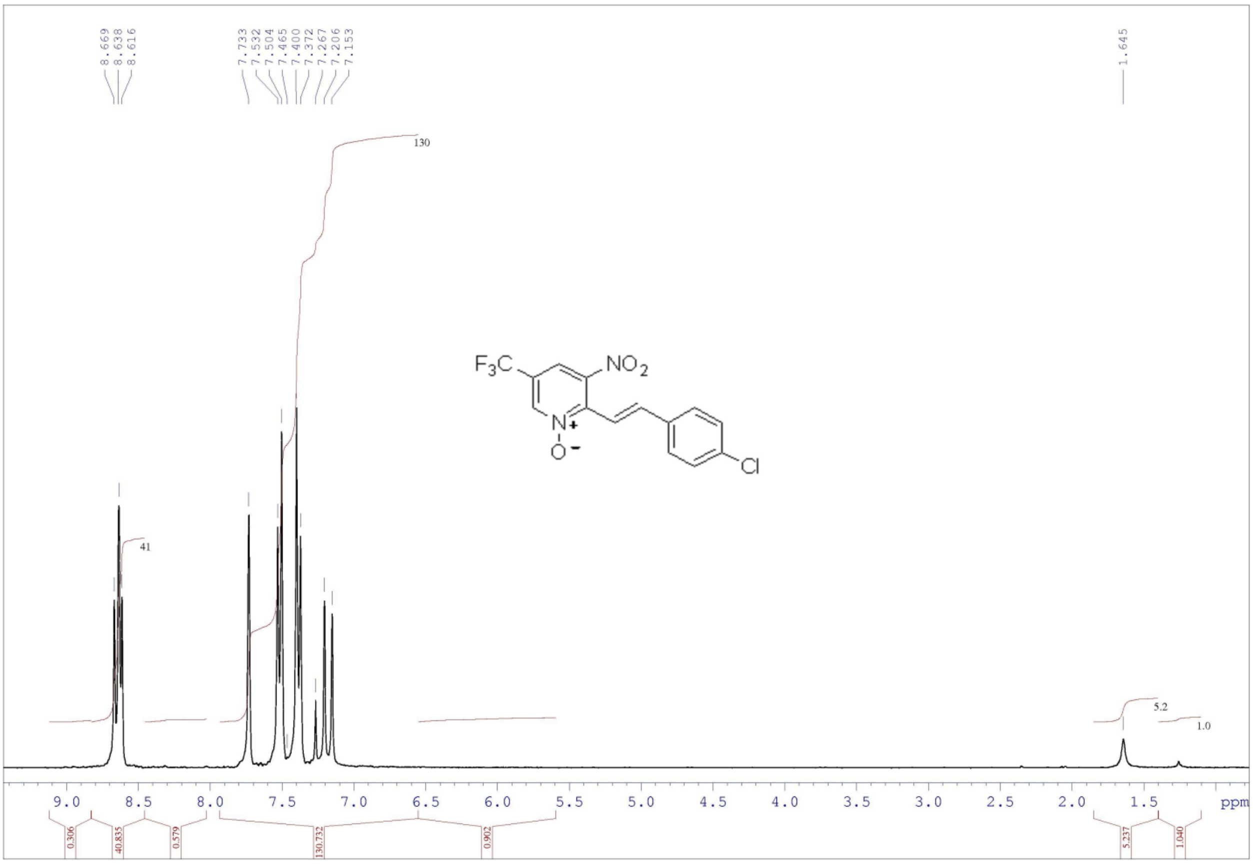

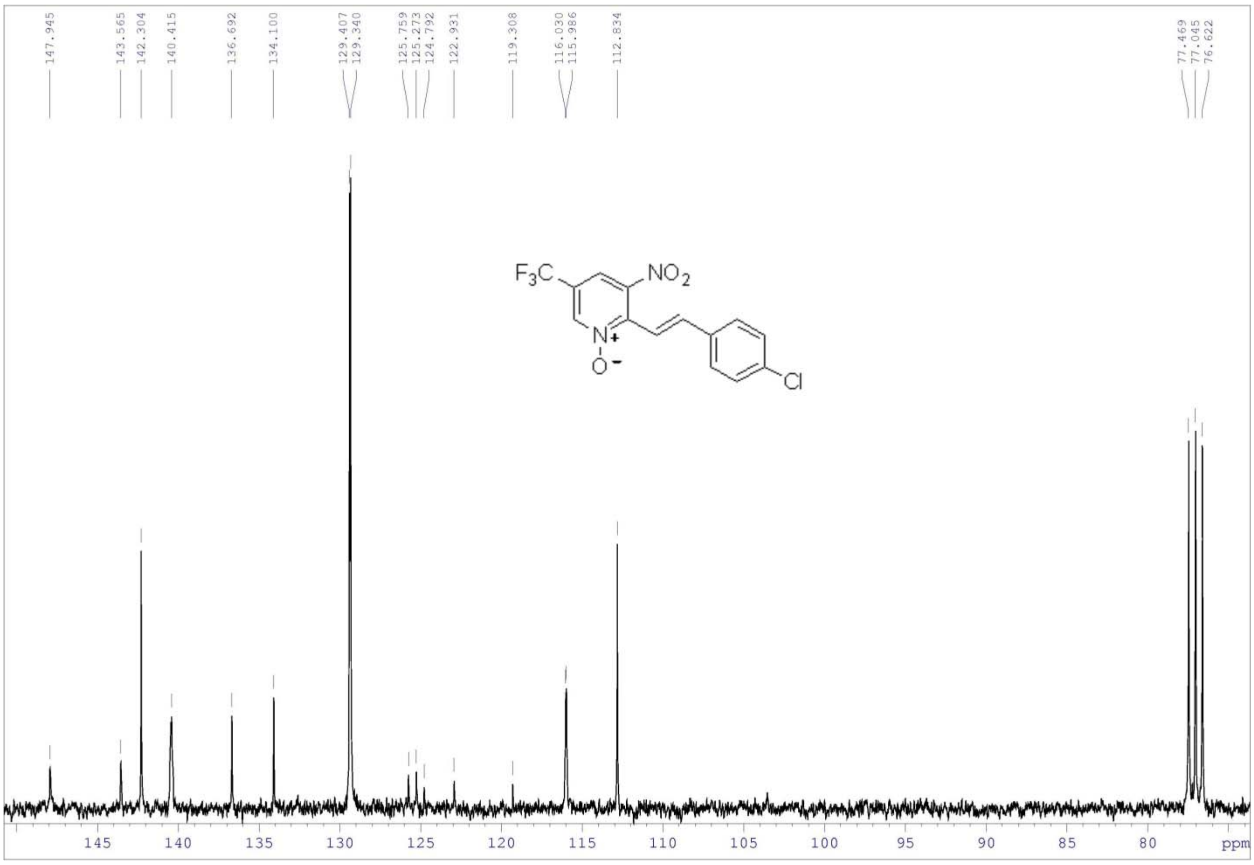

^{1}H and ^{13}C NMR spectra of compound **4f** in $CDCl_3$

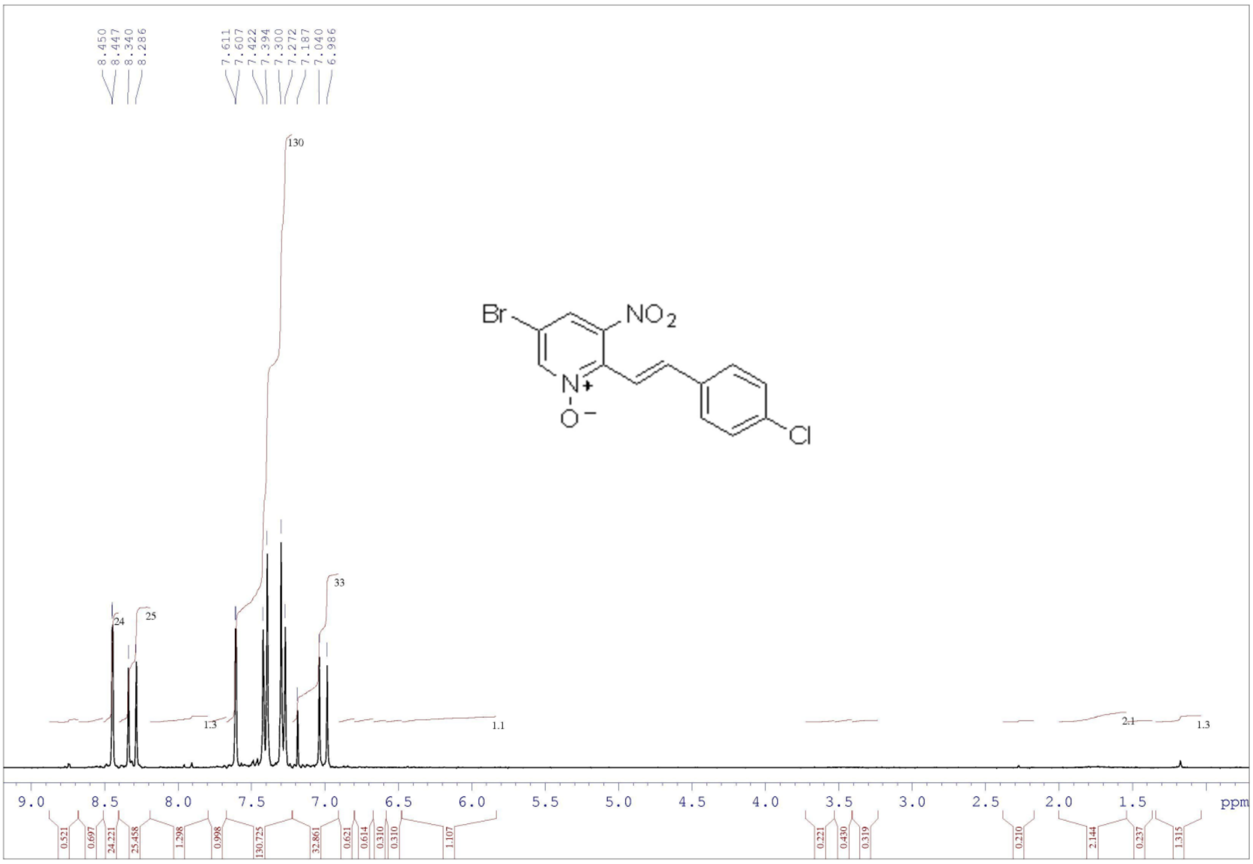

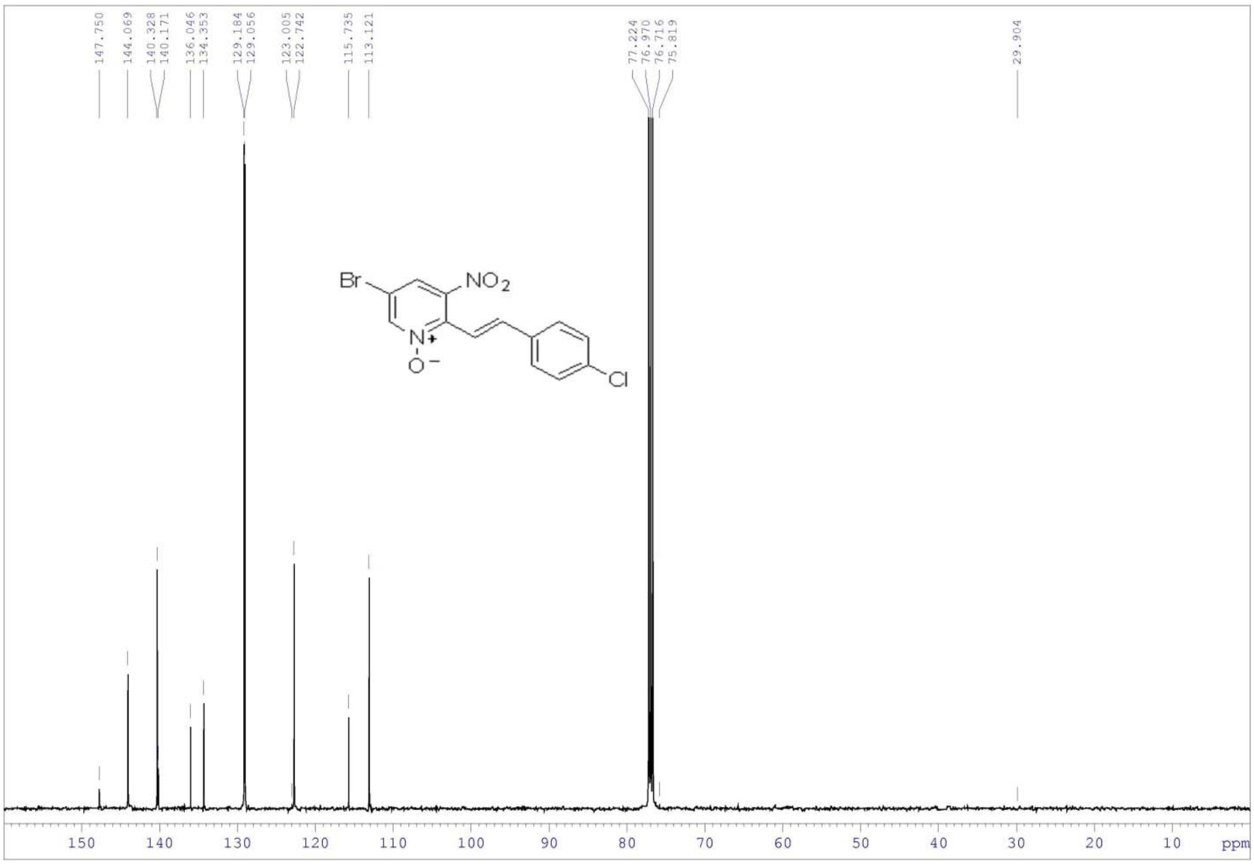

^{1}H and ^{13}C NMR spectra of compound **4g** in $CDCl_3$

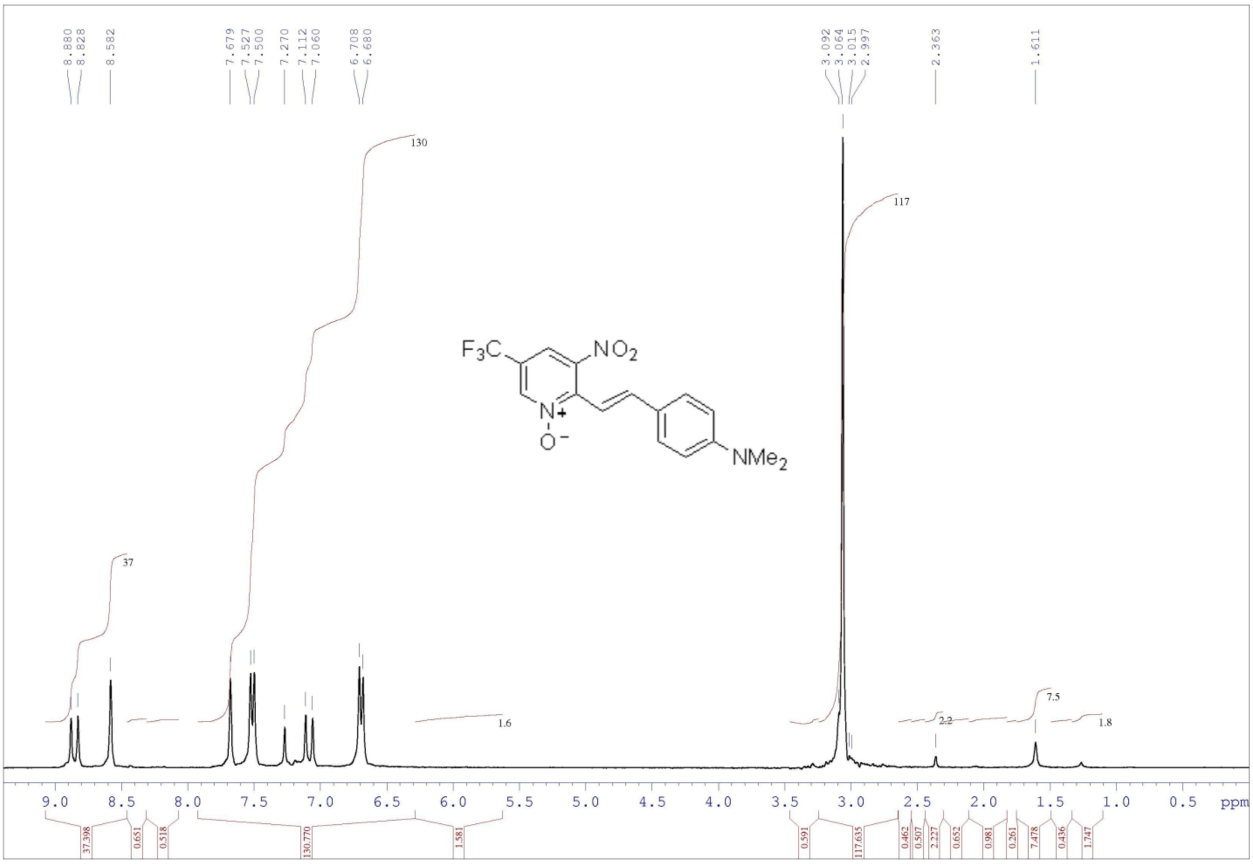

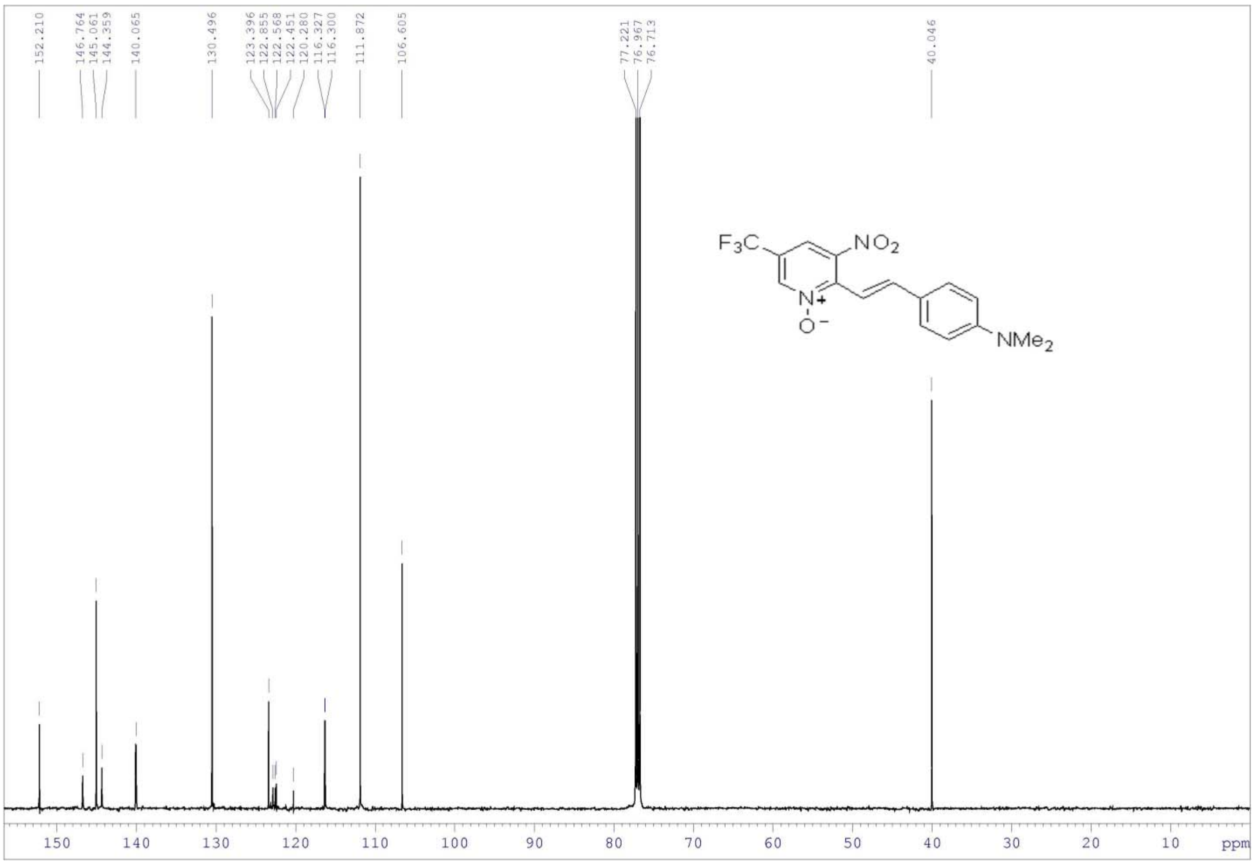

^{1}H and ^{13}C NMR spectra of compound **4h** in $CDCl_3$

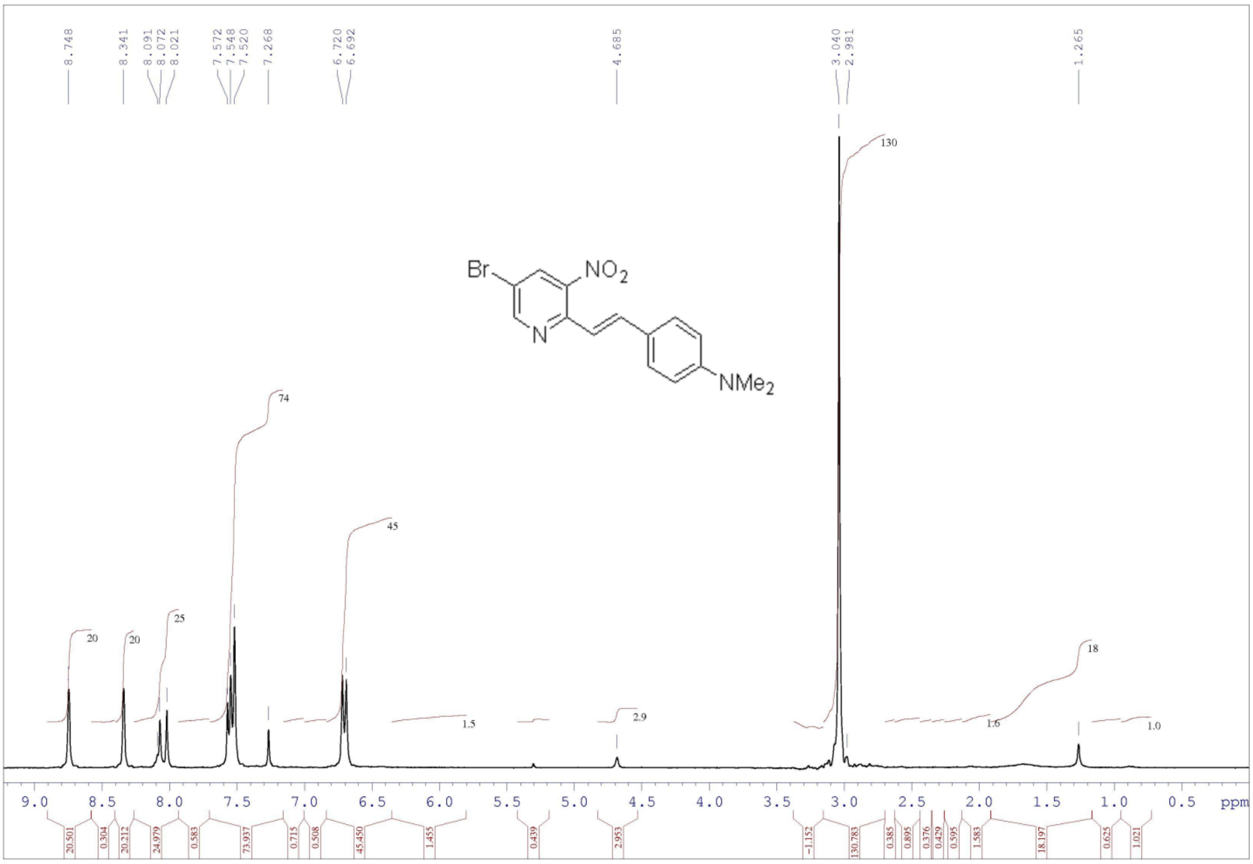

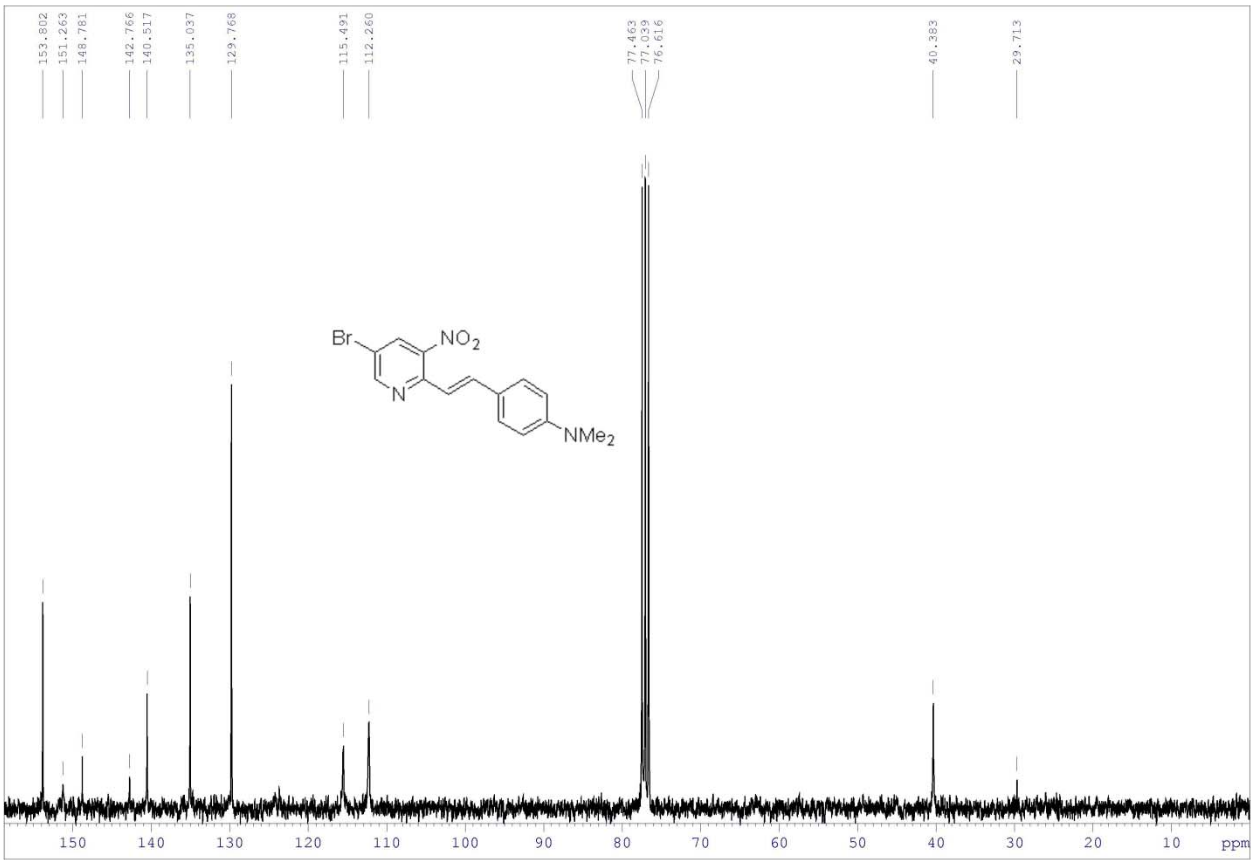

^{1}H and ^{13}C NMR spectra of compound **4i** in $CDCl_3$

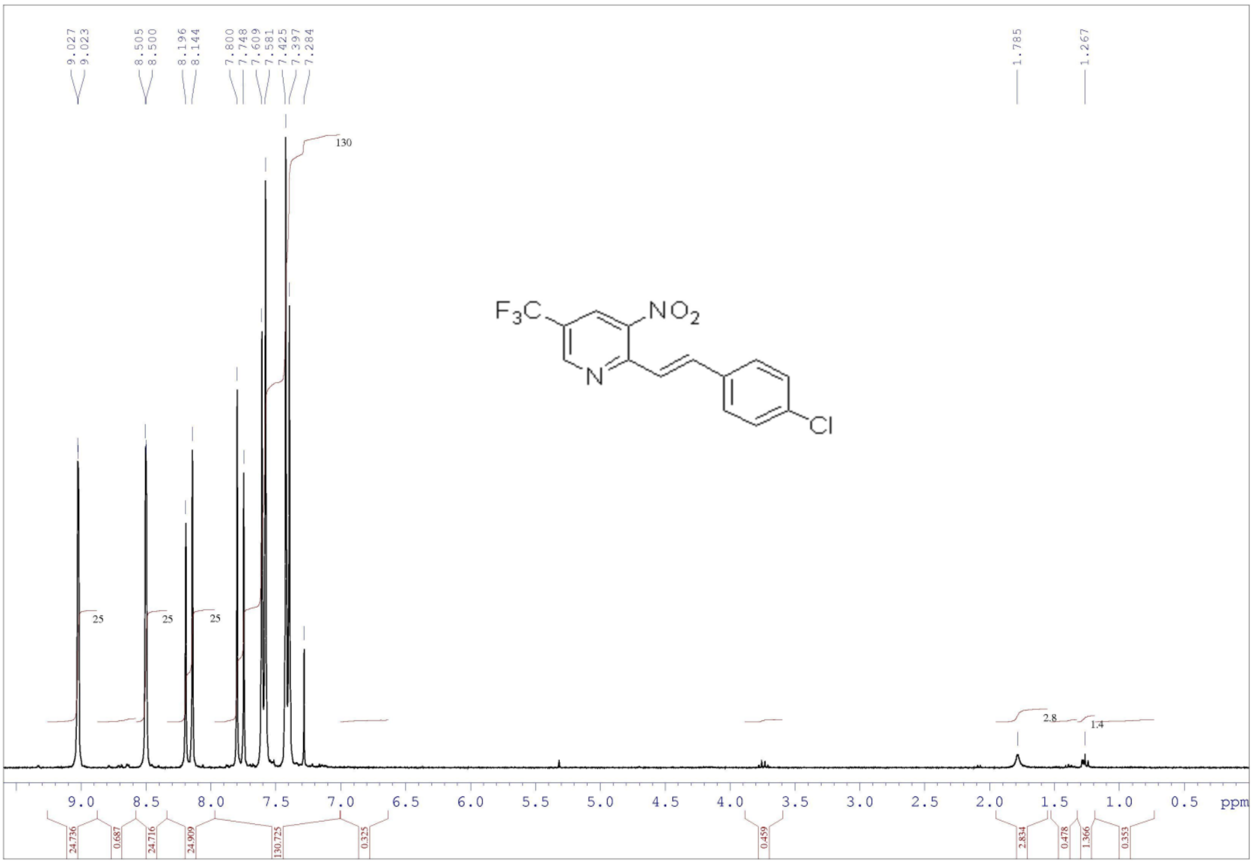

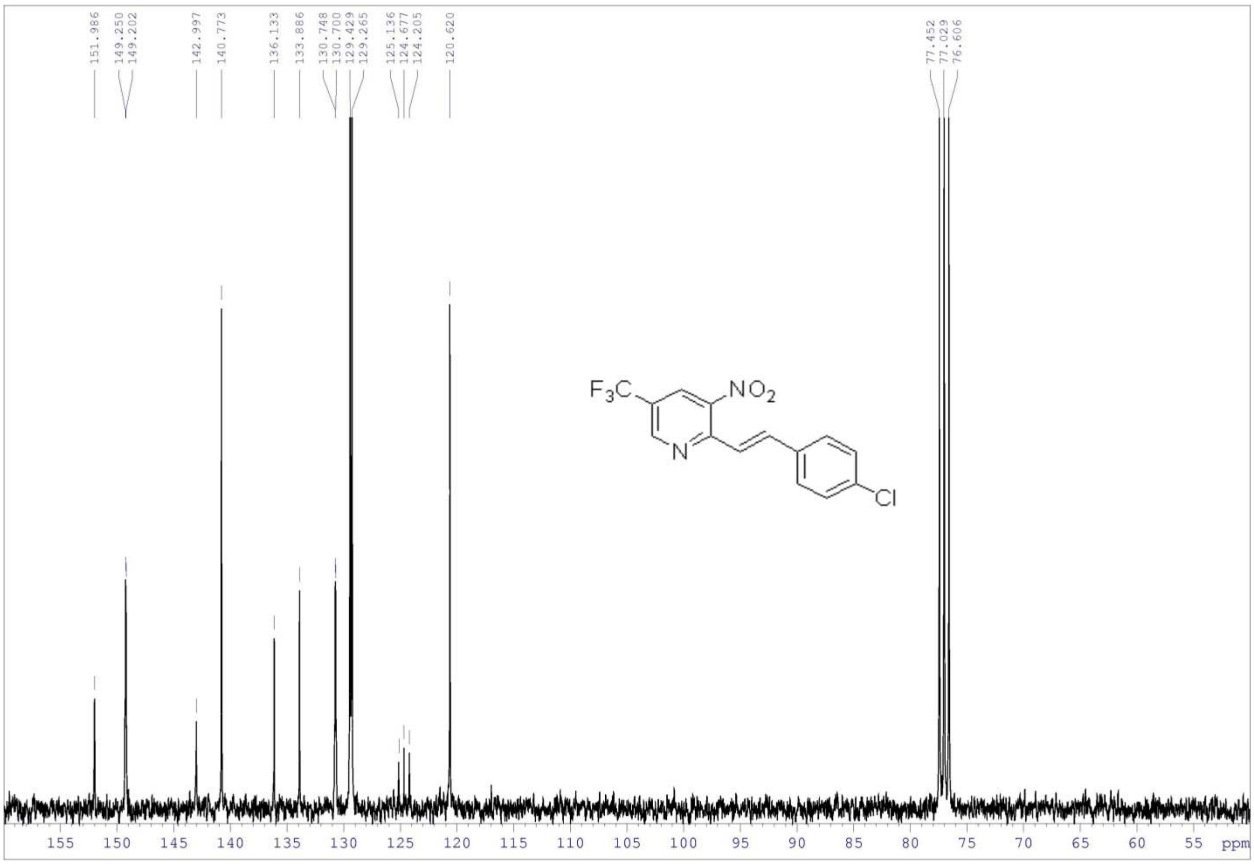

^{1}H and ^{13}C NMR spectra of compound **4j** in $CDCl_3$

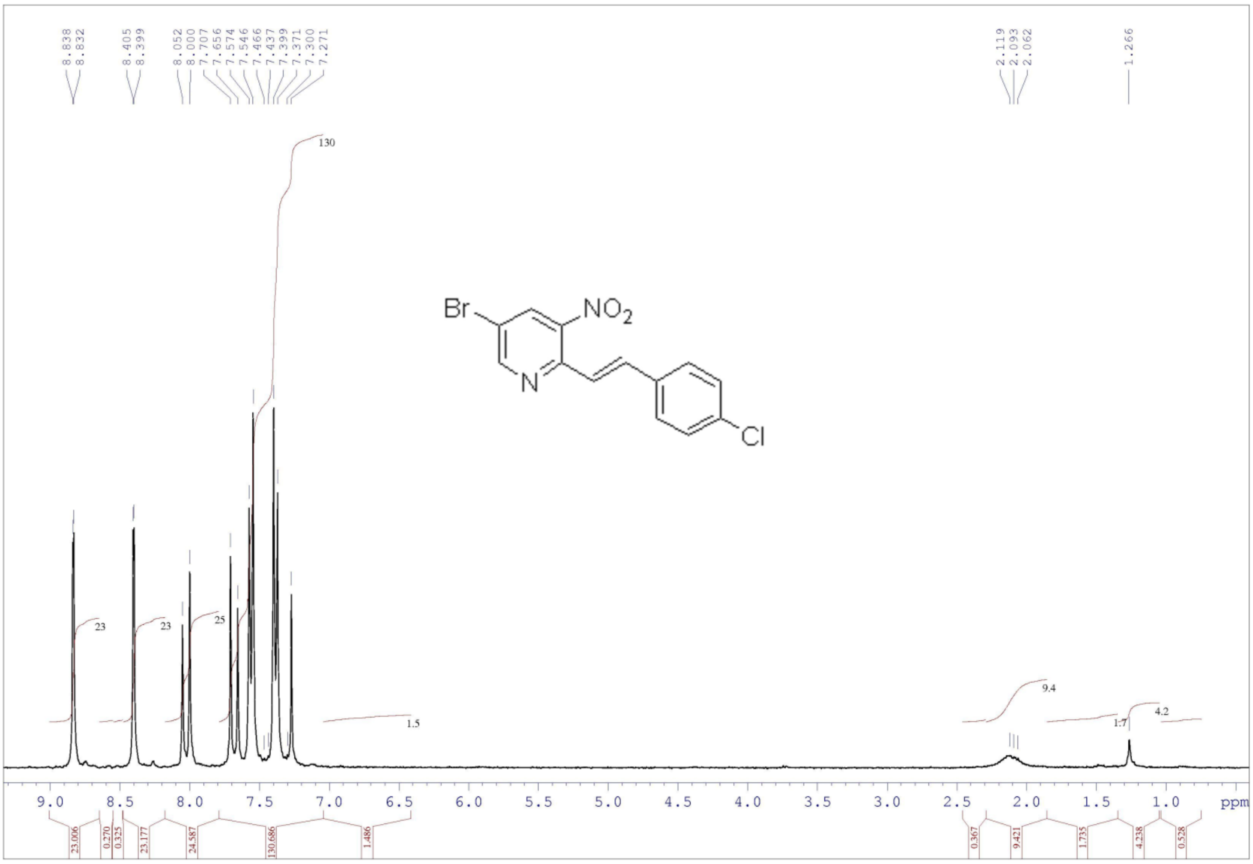

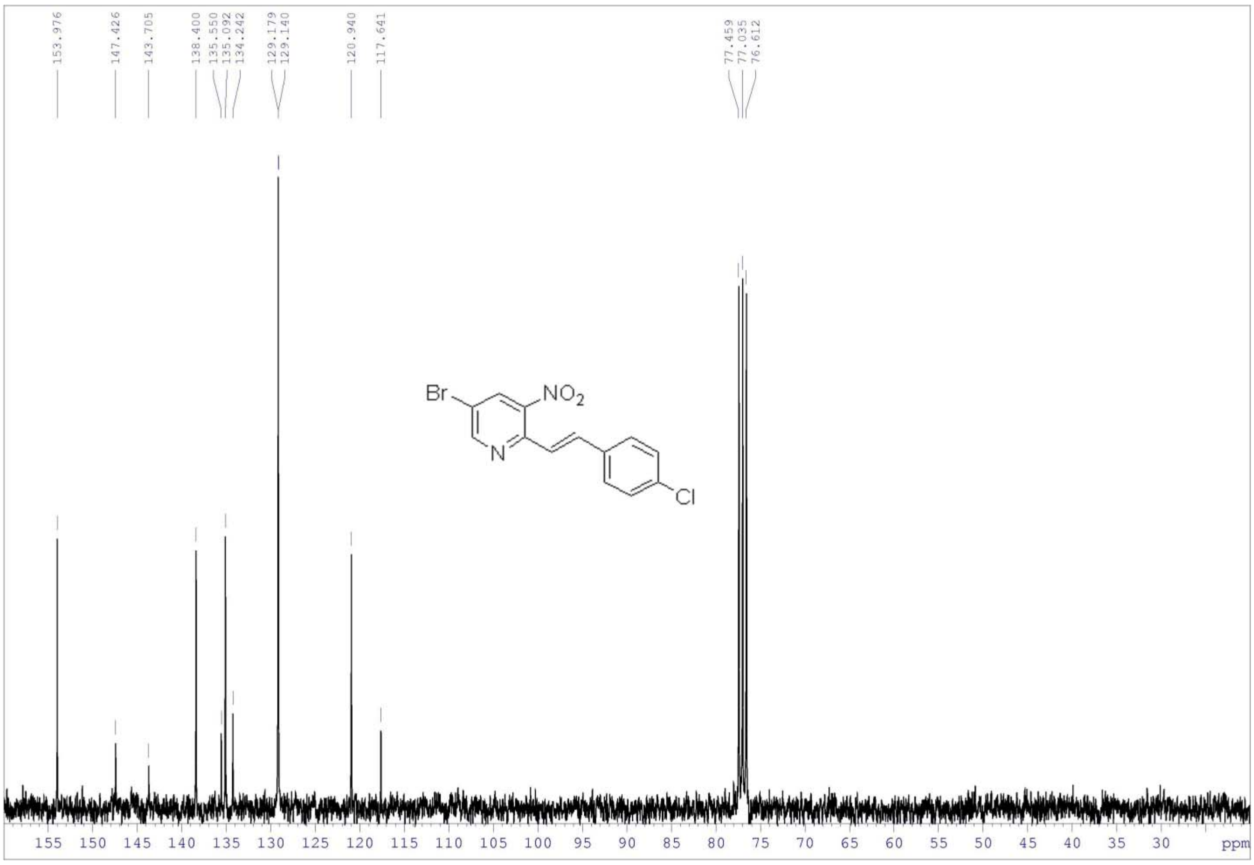

^{1}H and ^{13}C NMR spectra of compound **4k** in $CDCl_3$

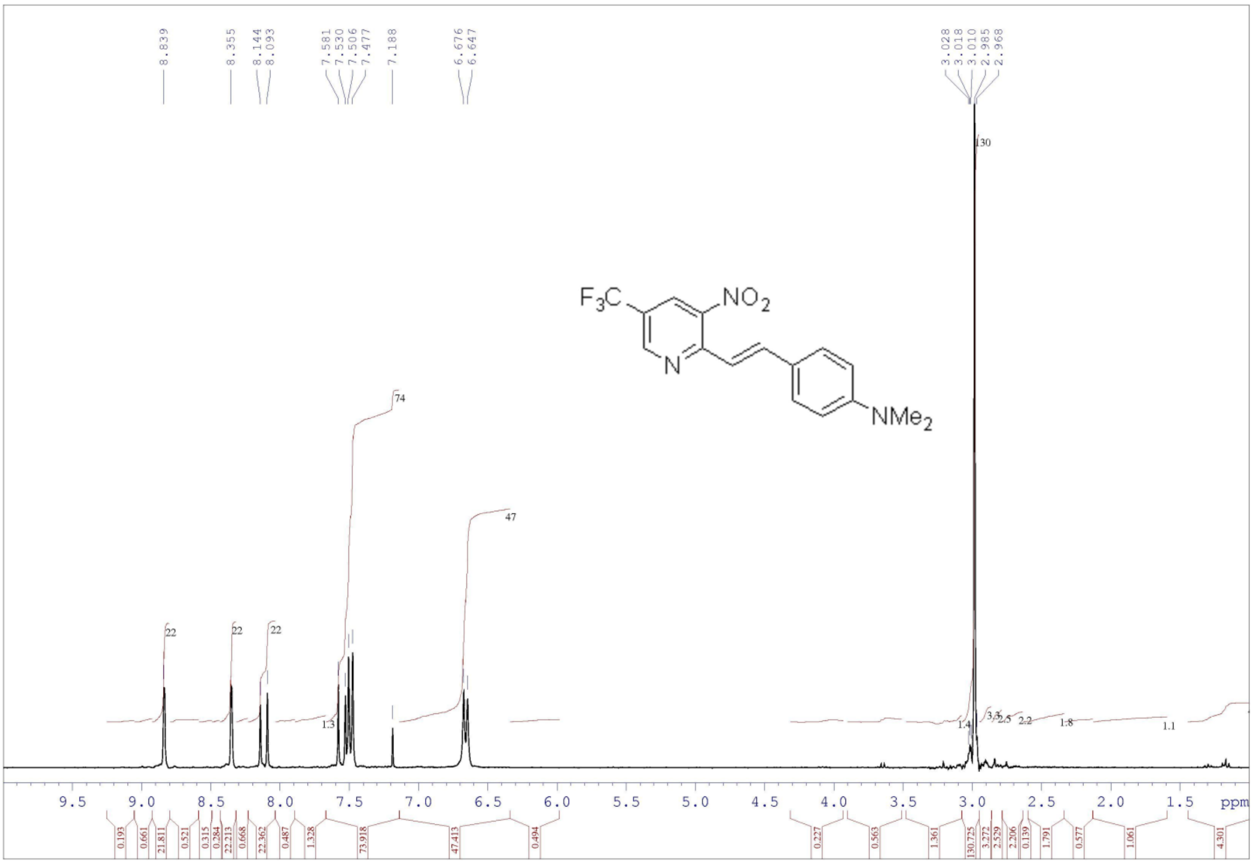

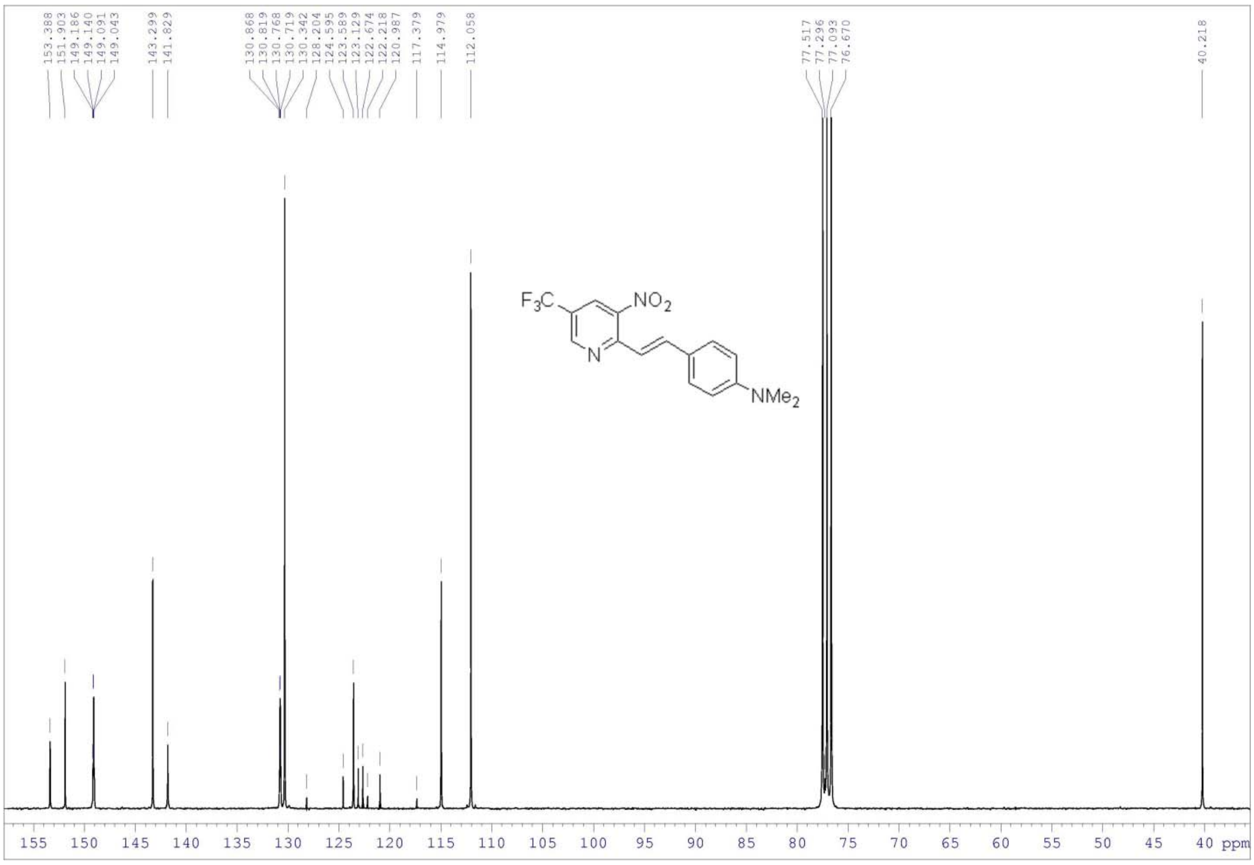

^{1}H and ^{13}C NMR spectra of compound **5a** in $CDCl_3$

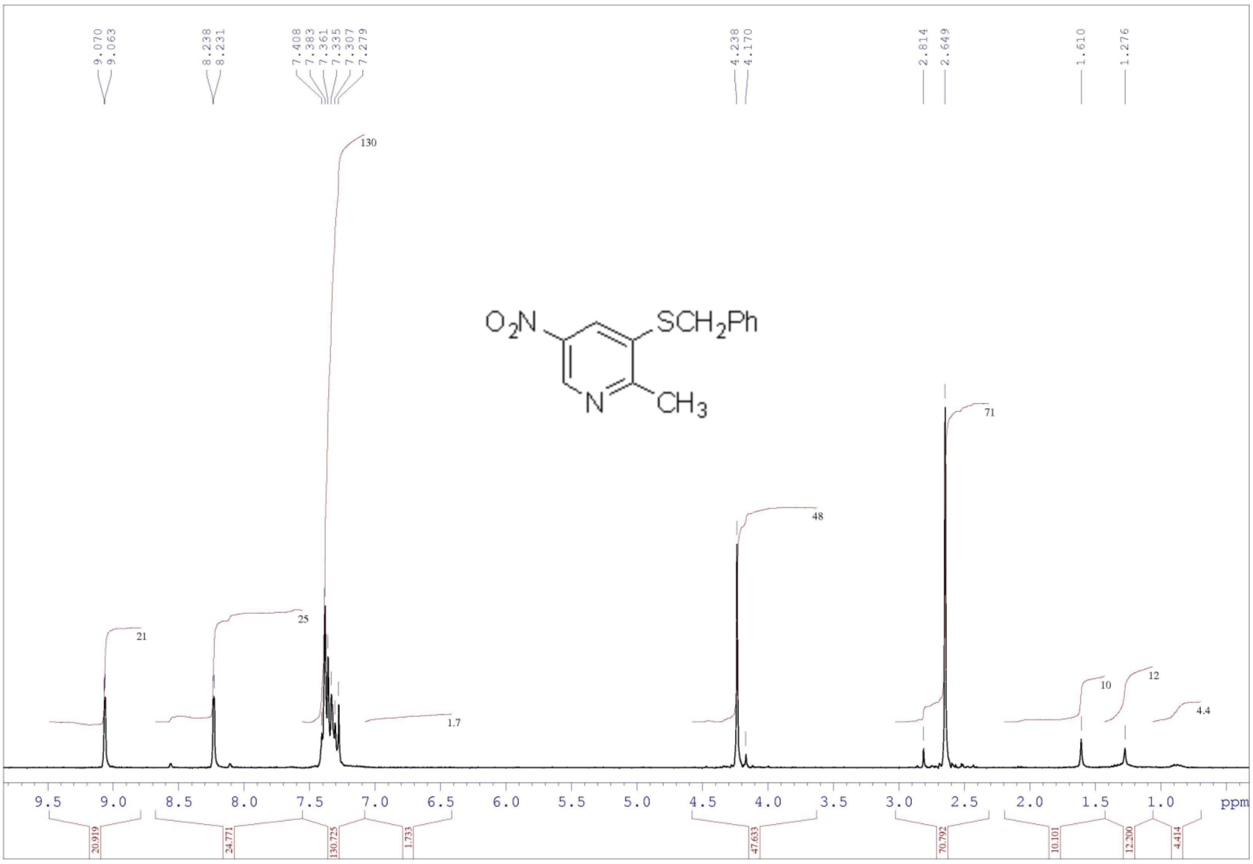

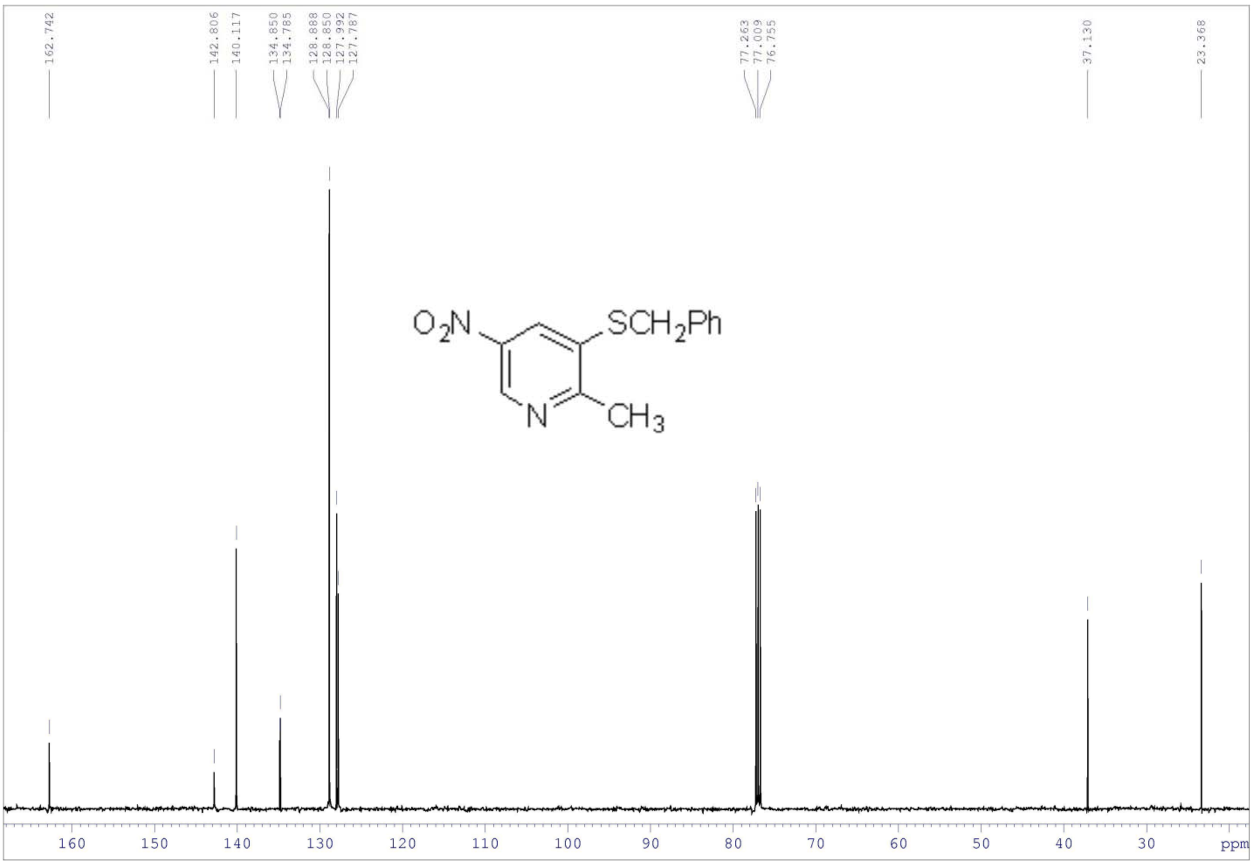

^{1}H and ^{13}C NMR spectra of compound **5b** in $CDCl_3$

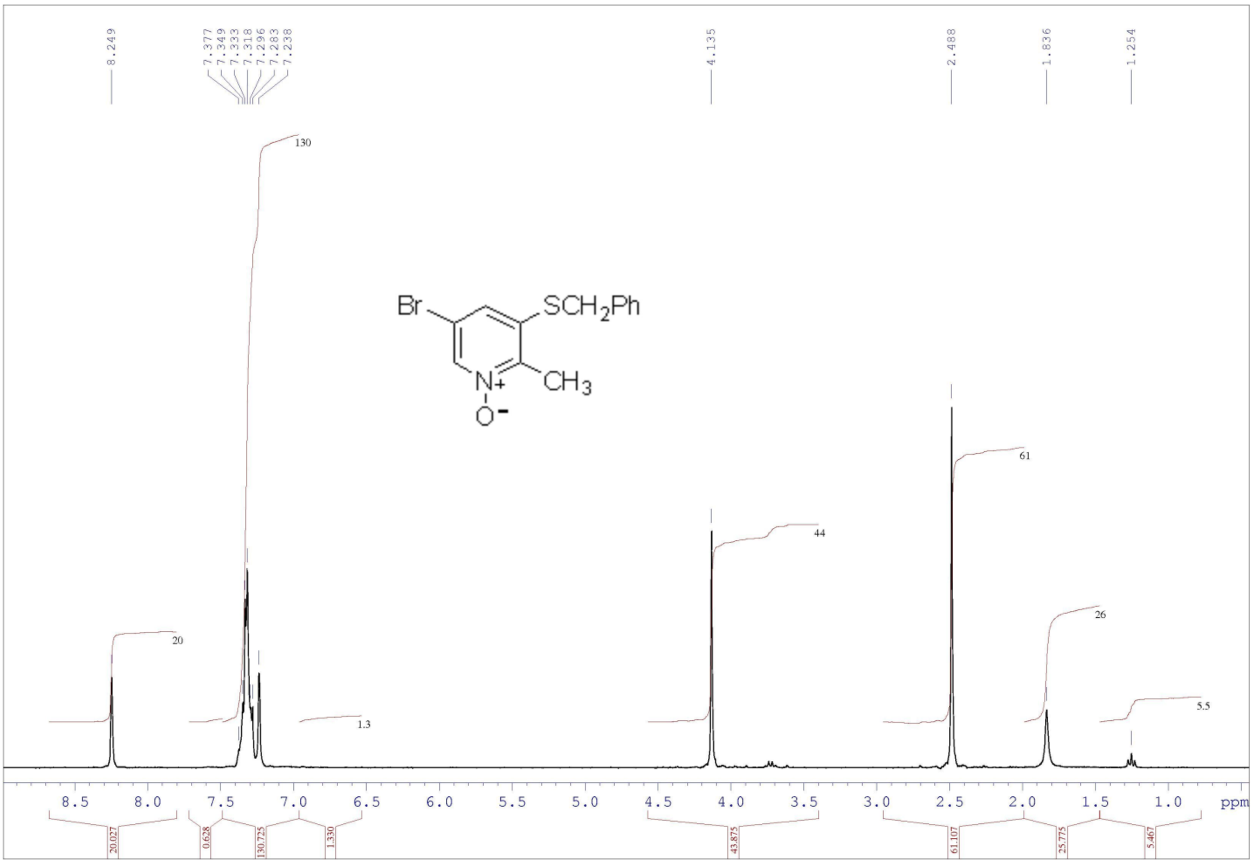

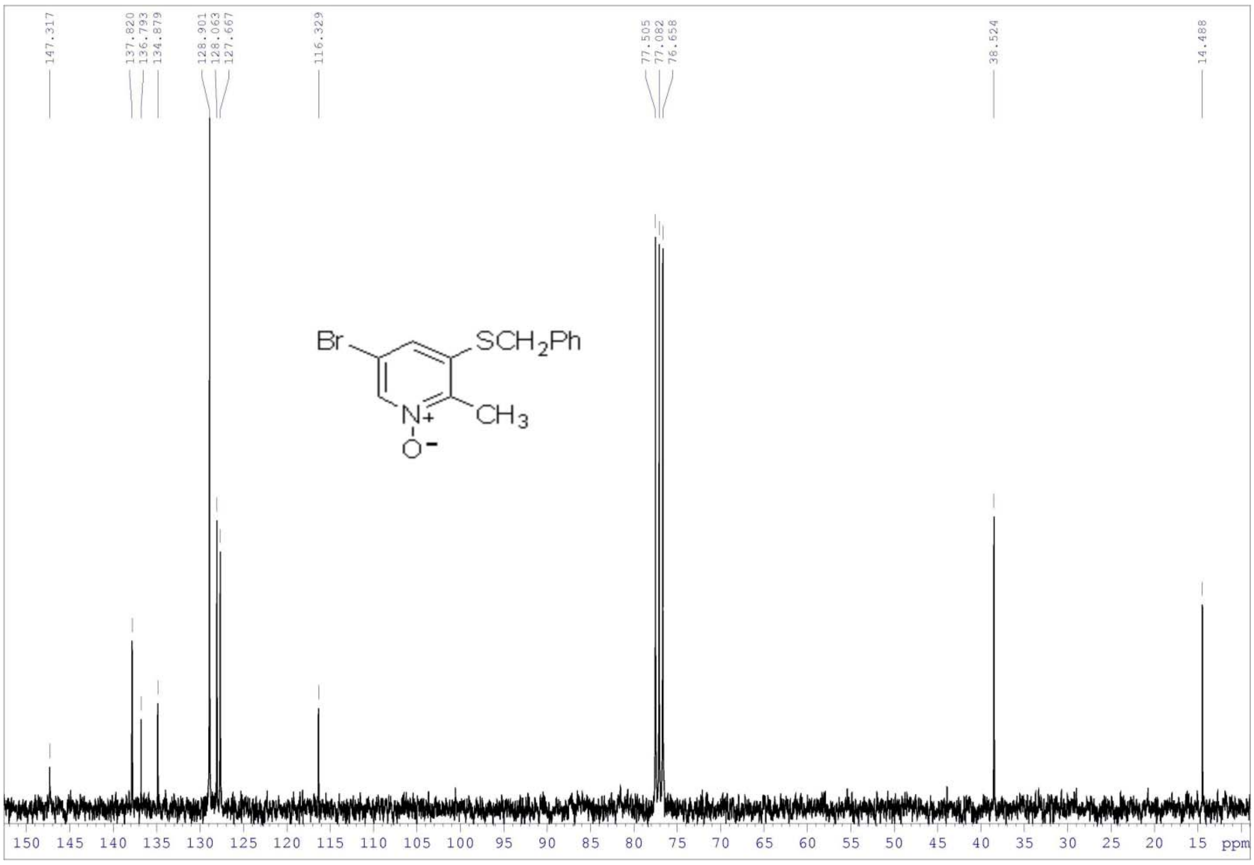

^{1}H and ^{13}C NMR spectra of compound **5c** in $CDCl_3$

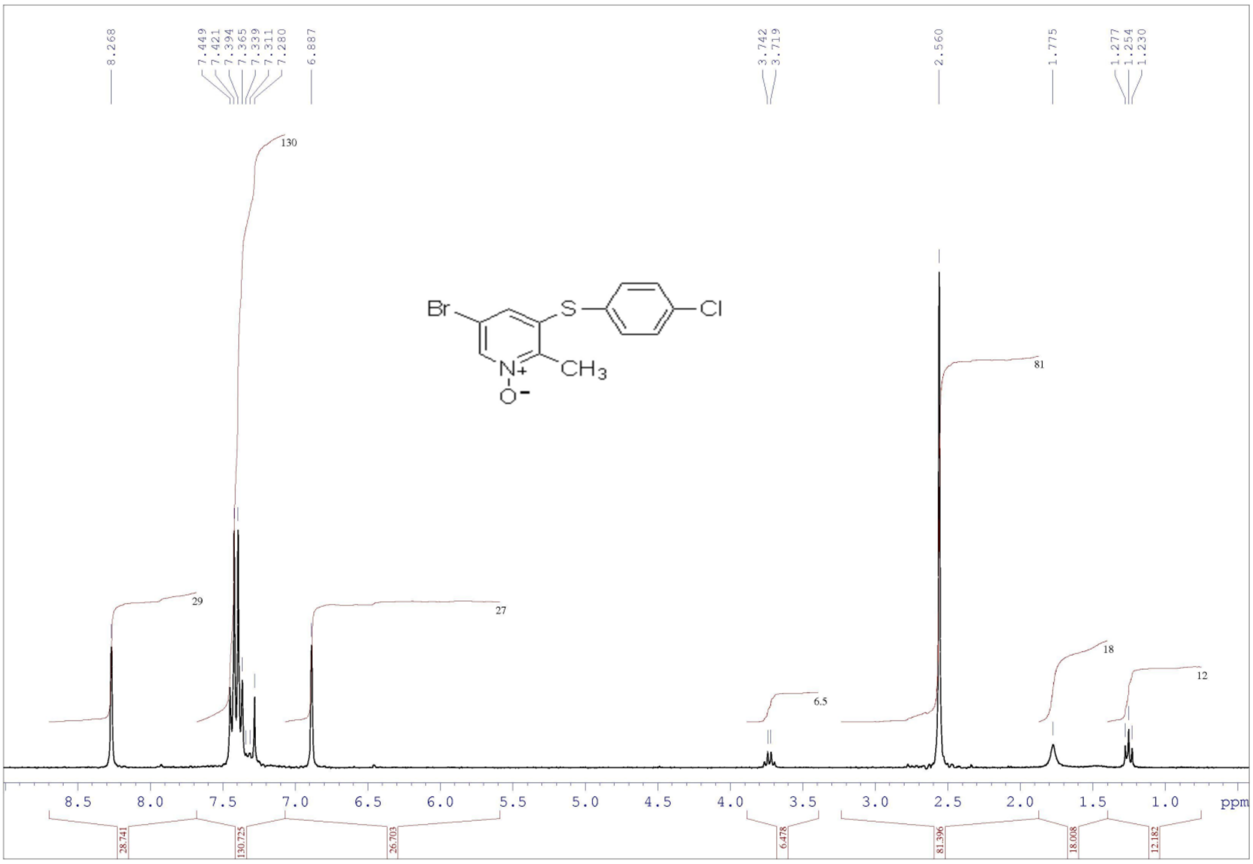

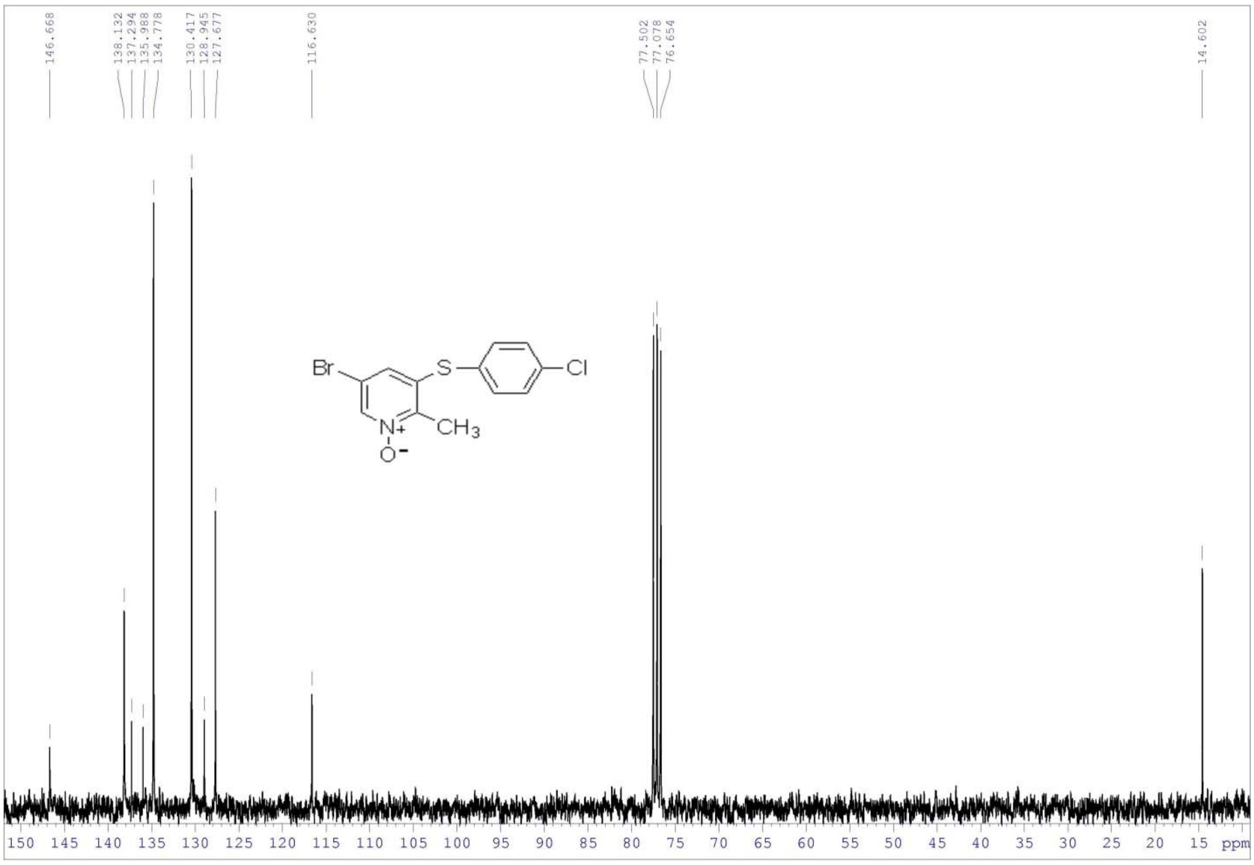

^{1}H and ^{13}C NMR spectra of compound **5d** in $CDCl_3$

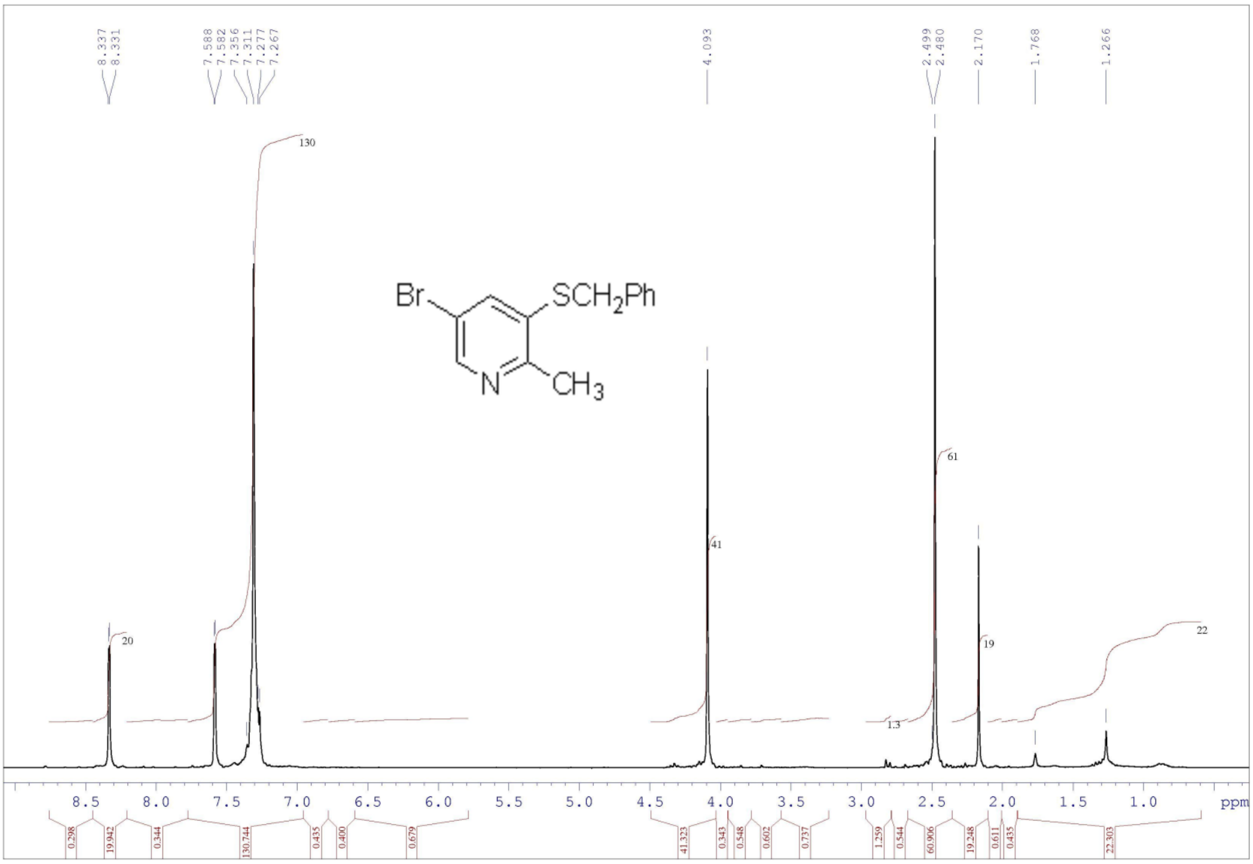

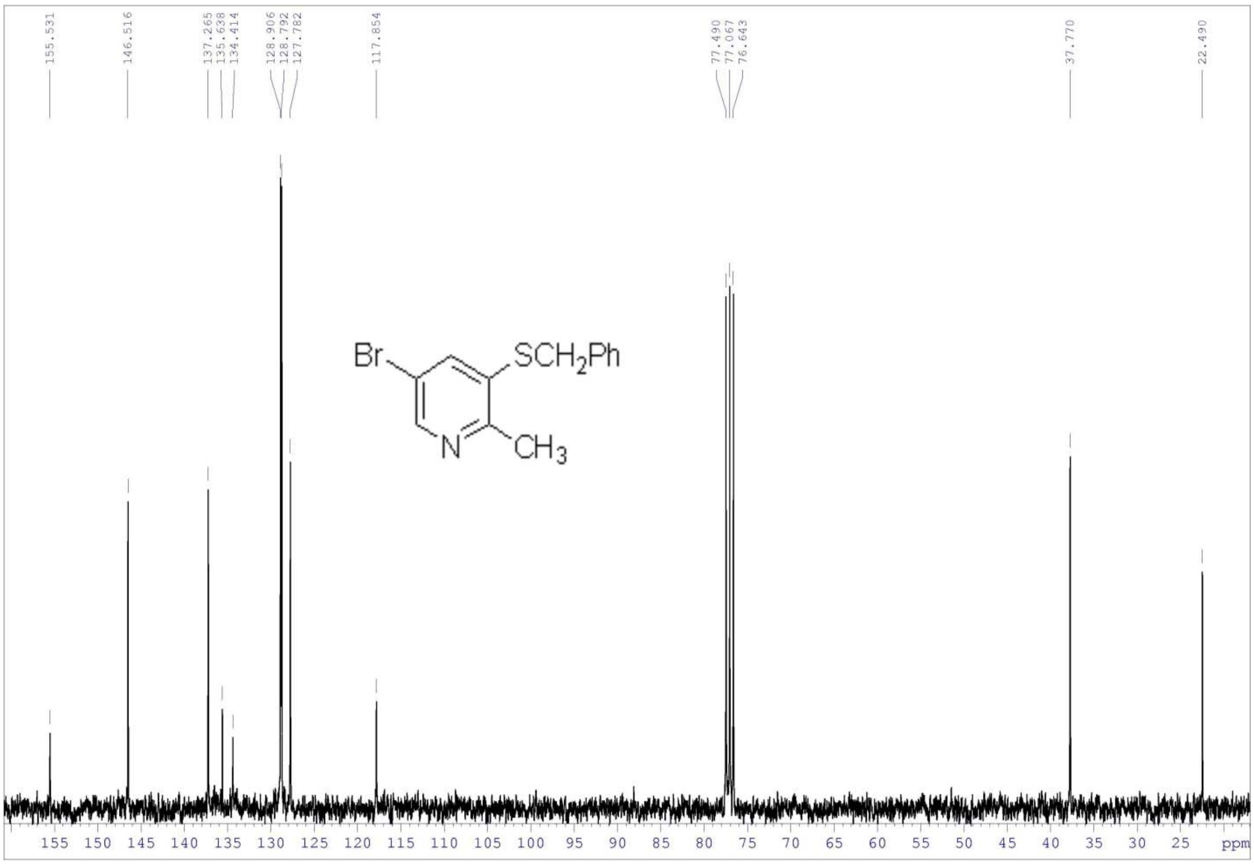

^{1}H and ^{13}C NMR spectra of compound **5e** in $CDCl_3$

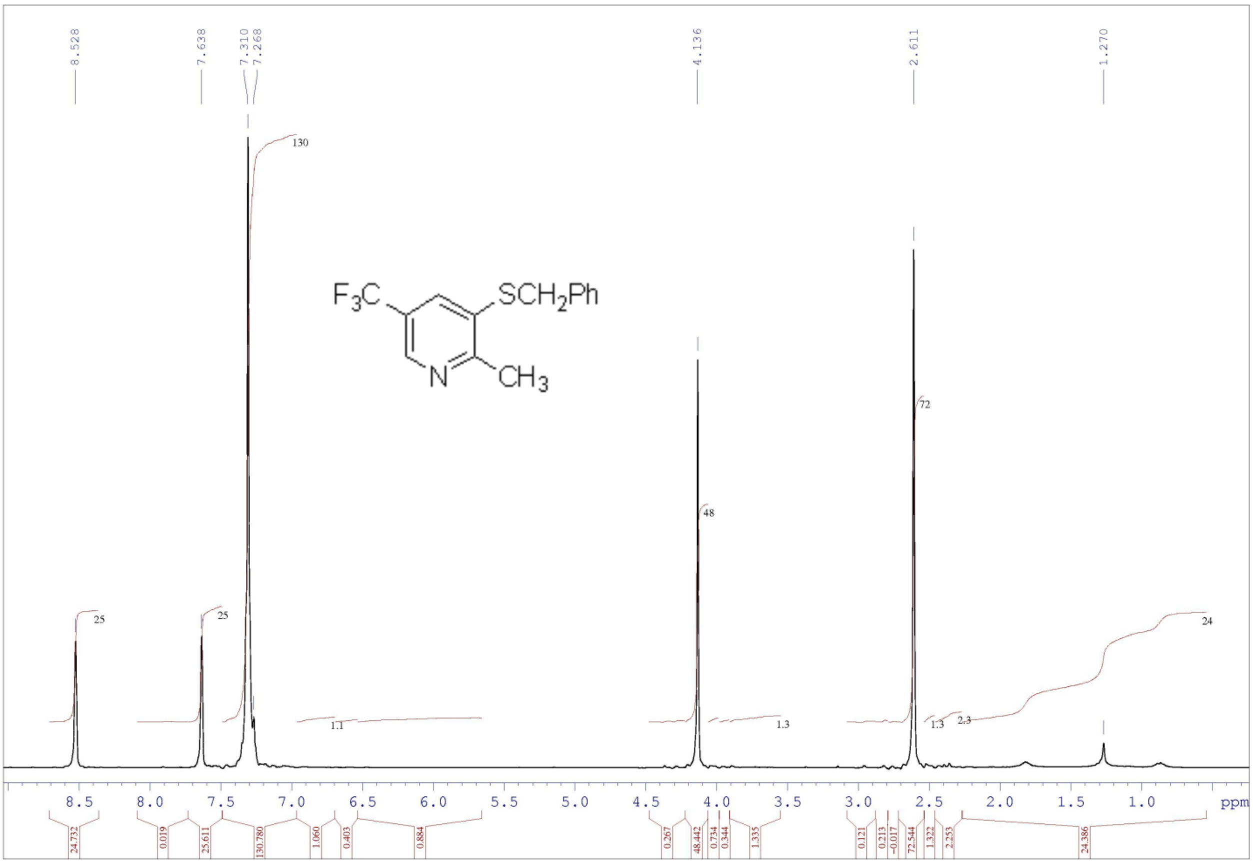

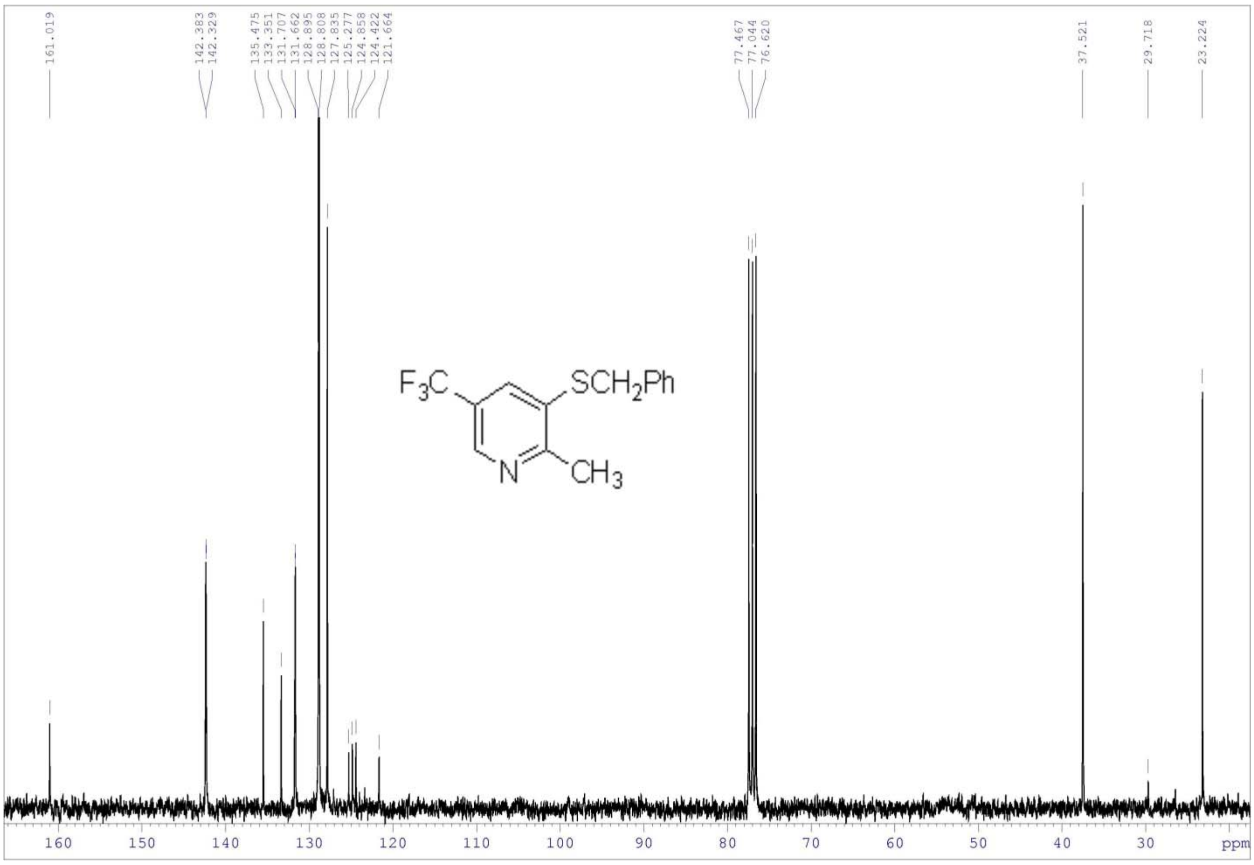

^{1}H and ^{13}C NMR spectra of compound **5f** in $CDCl_3$

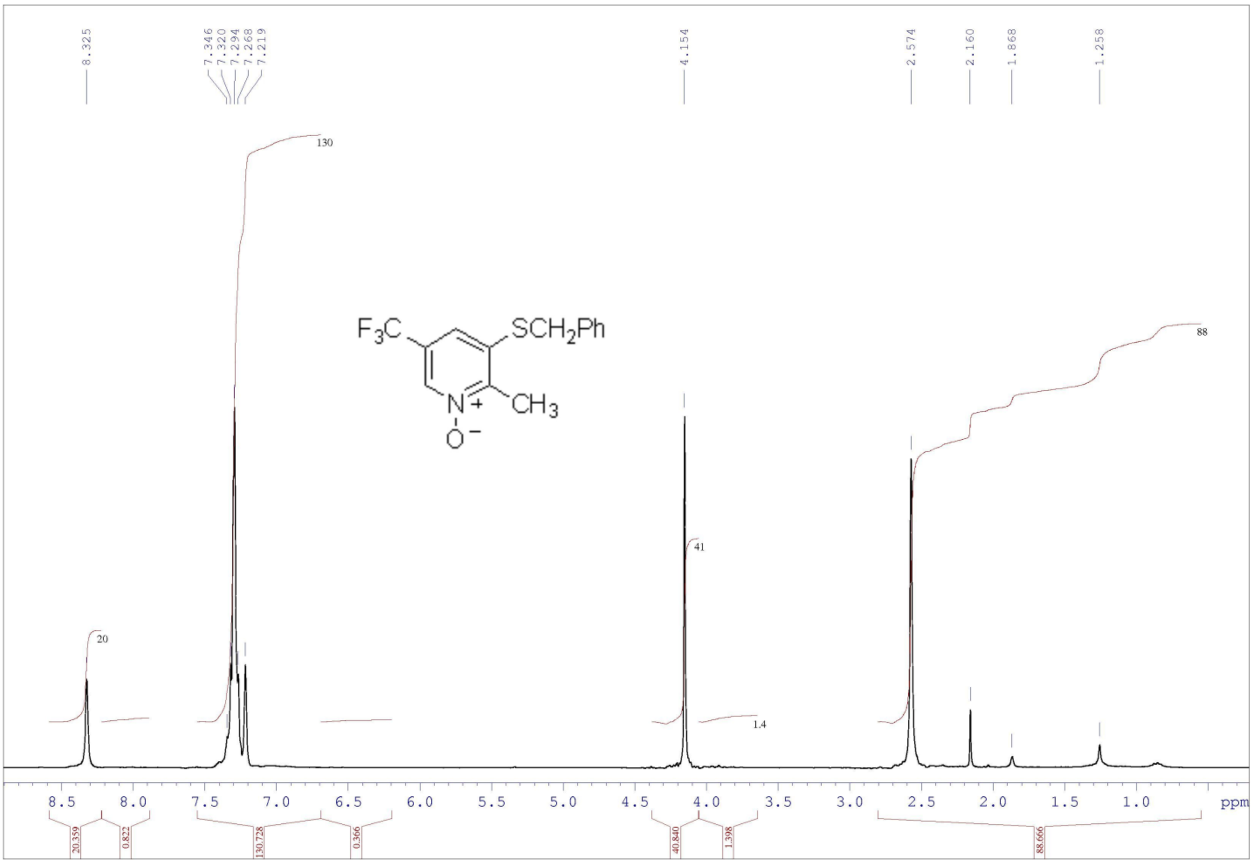

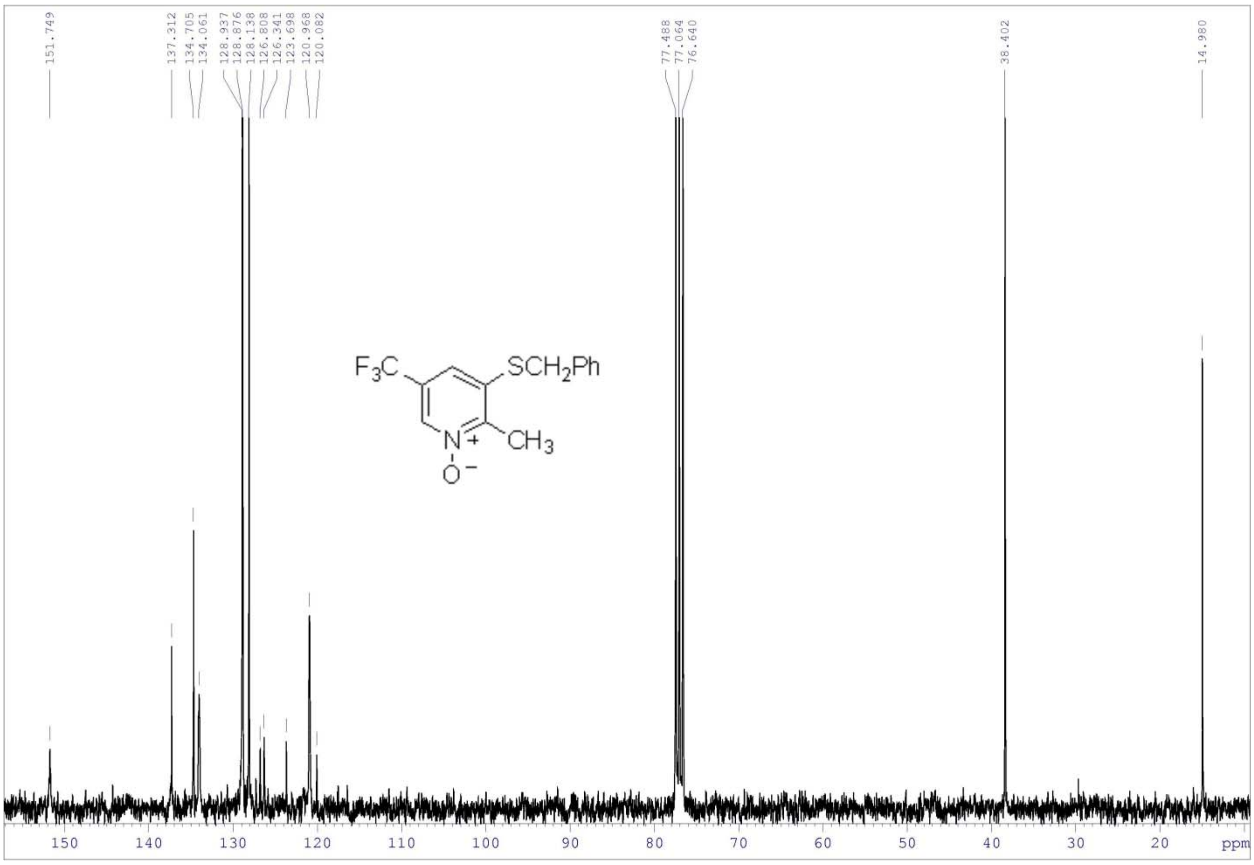

^{1}H and ^{13}C NMR spectra of compound **5g** in $CDCl_3$

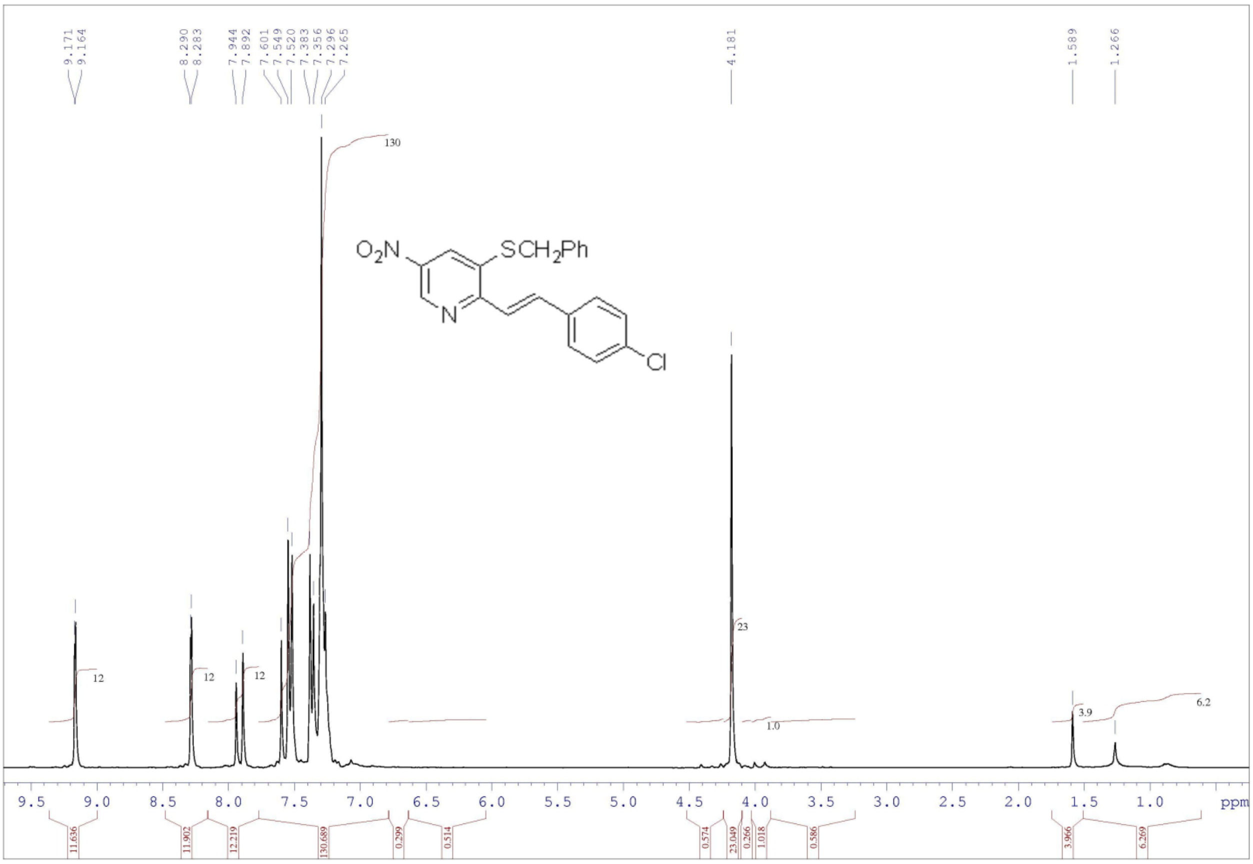

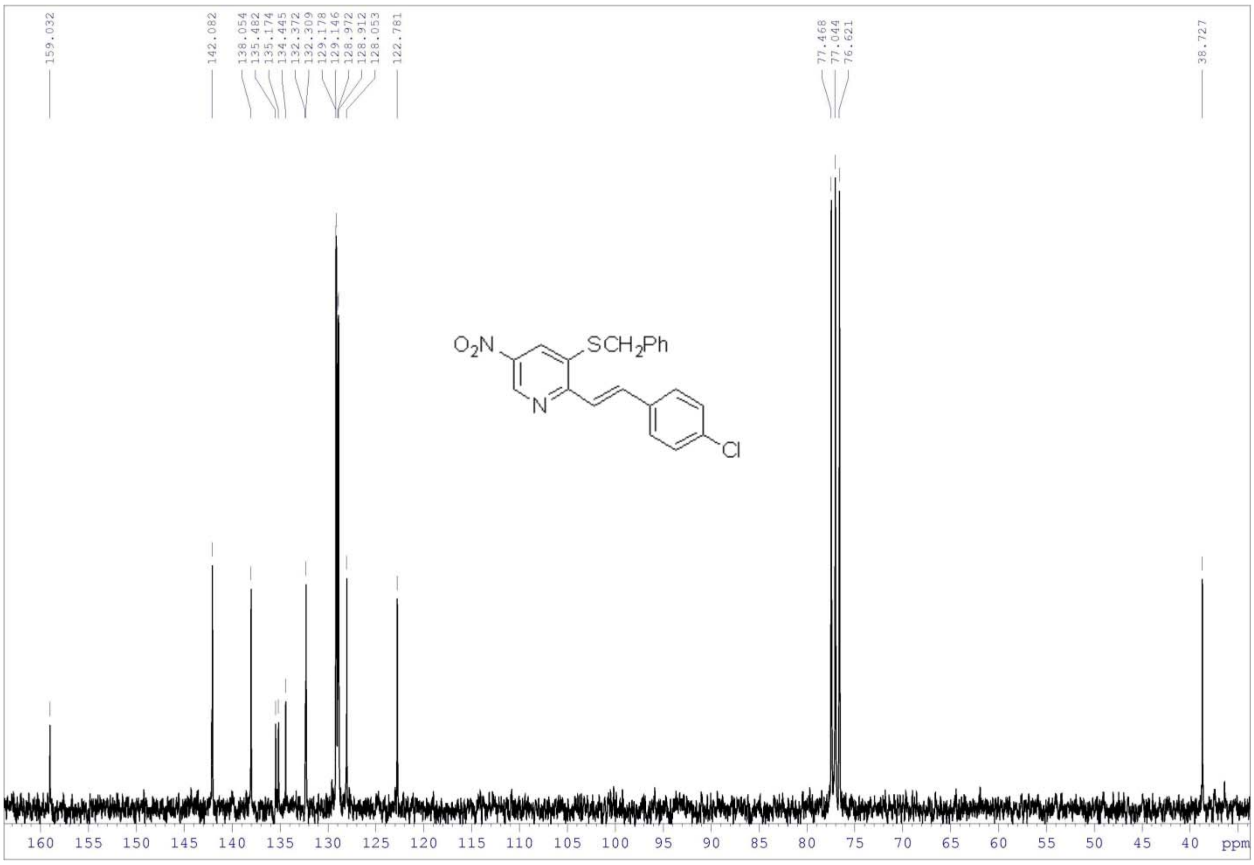

^{1}H and ^{13}C NMR spectra of compound **5h** in $CDCl_3$

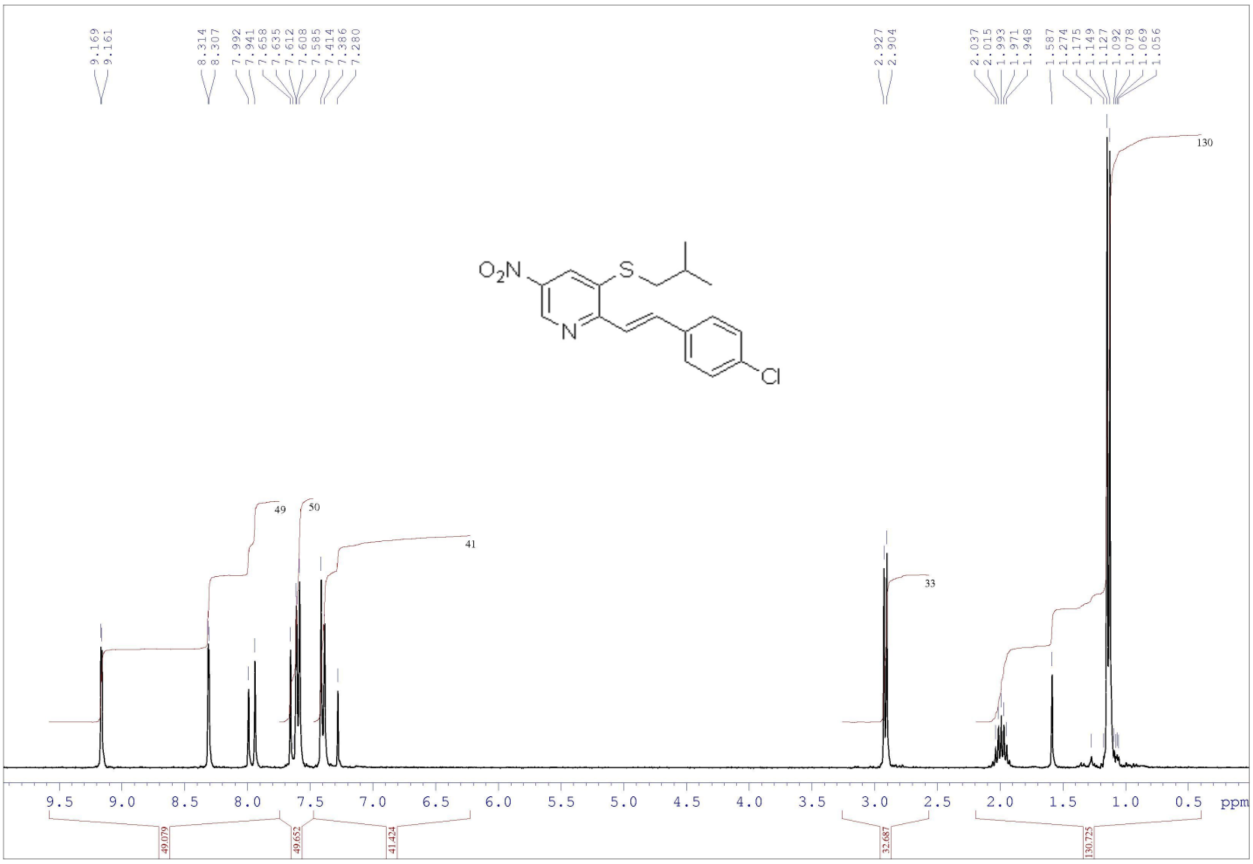

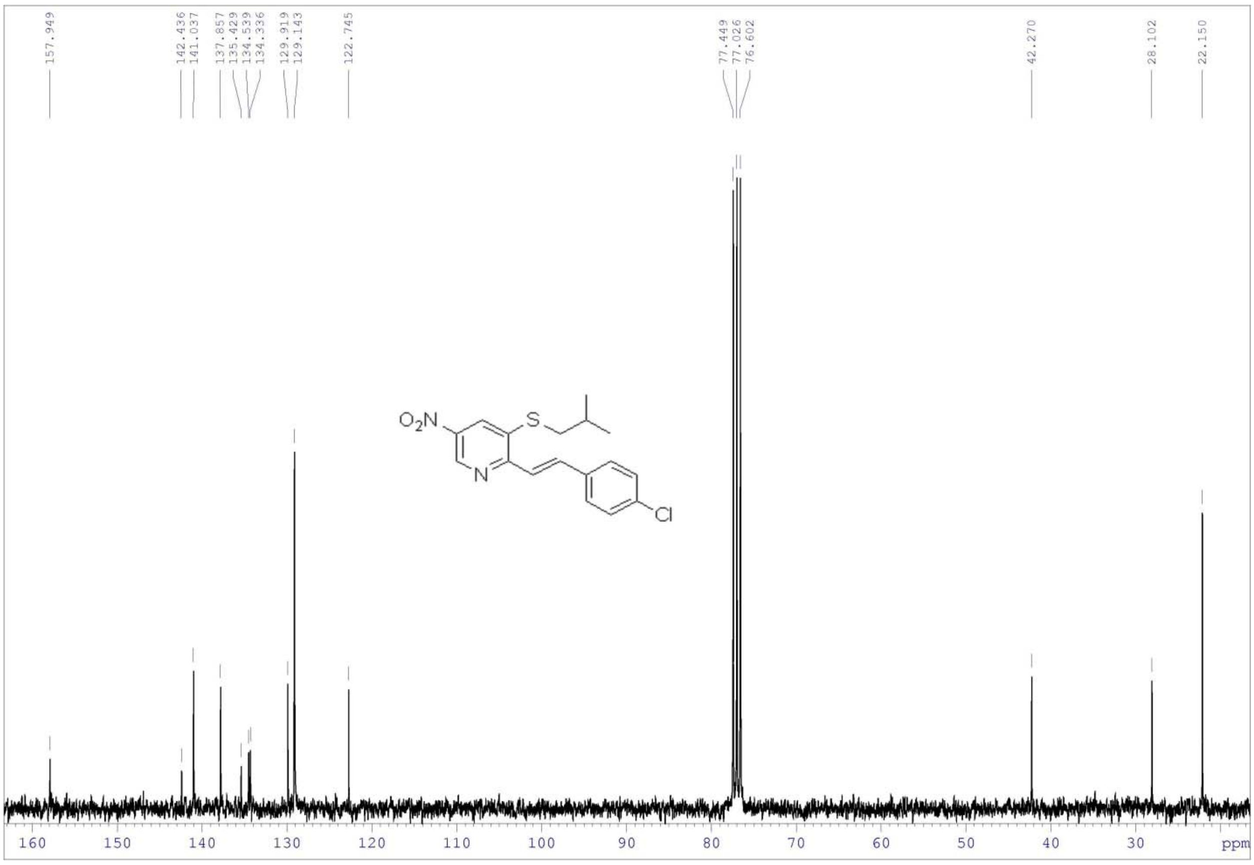

^{1}H NMR spectrum of compound **5i** in $CDCl_3$

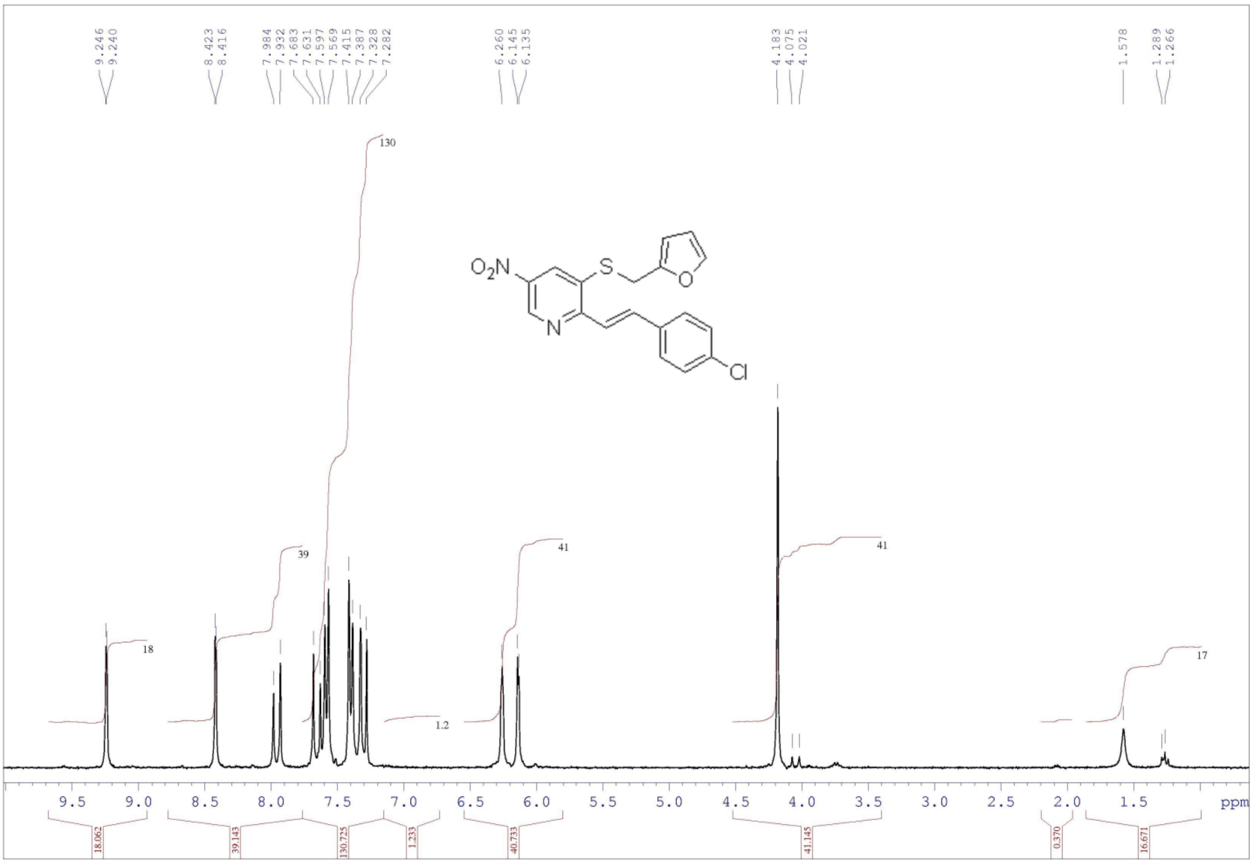

^{1}H NMR spectrum of compound **5j** in $CDCl_3$

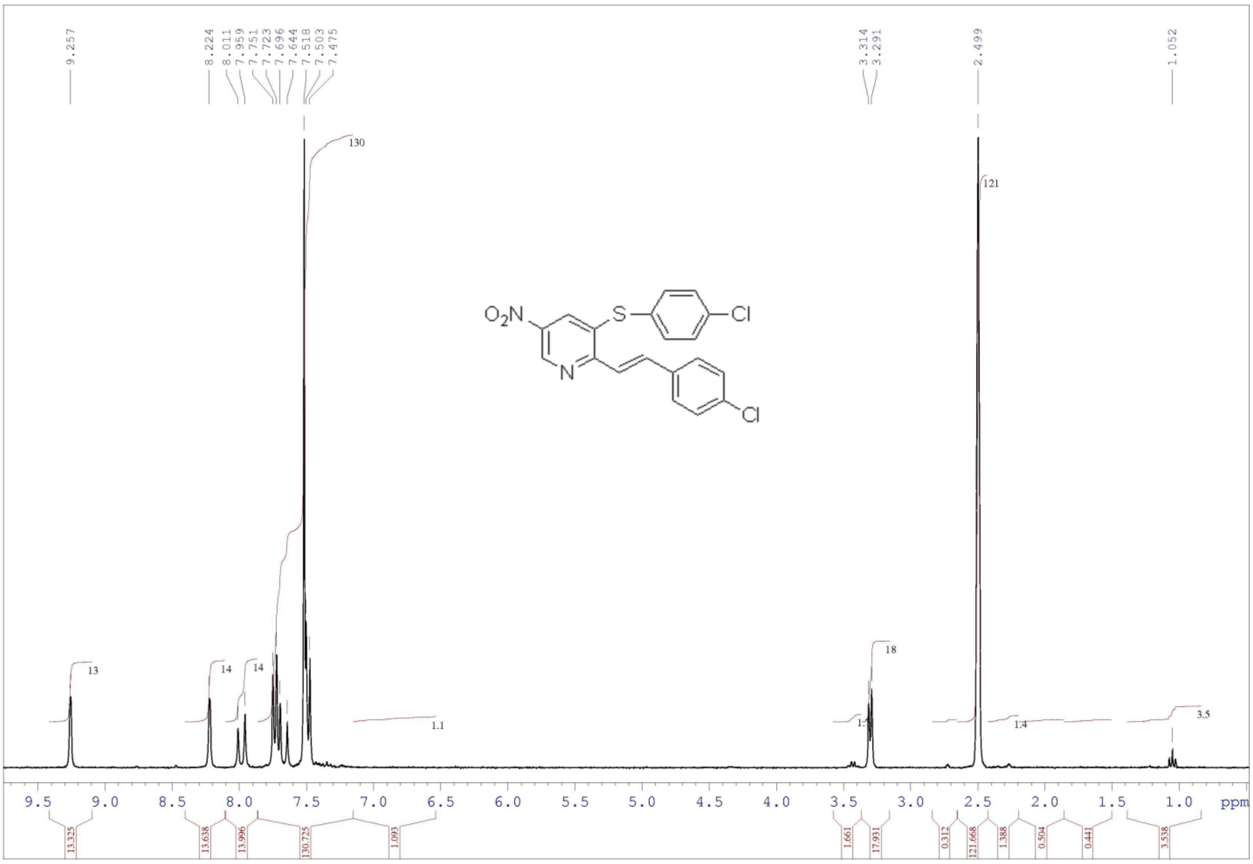

^{1}H NMR spectrum of compound **5k** in DMSO-d_6

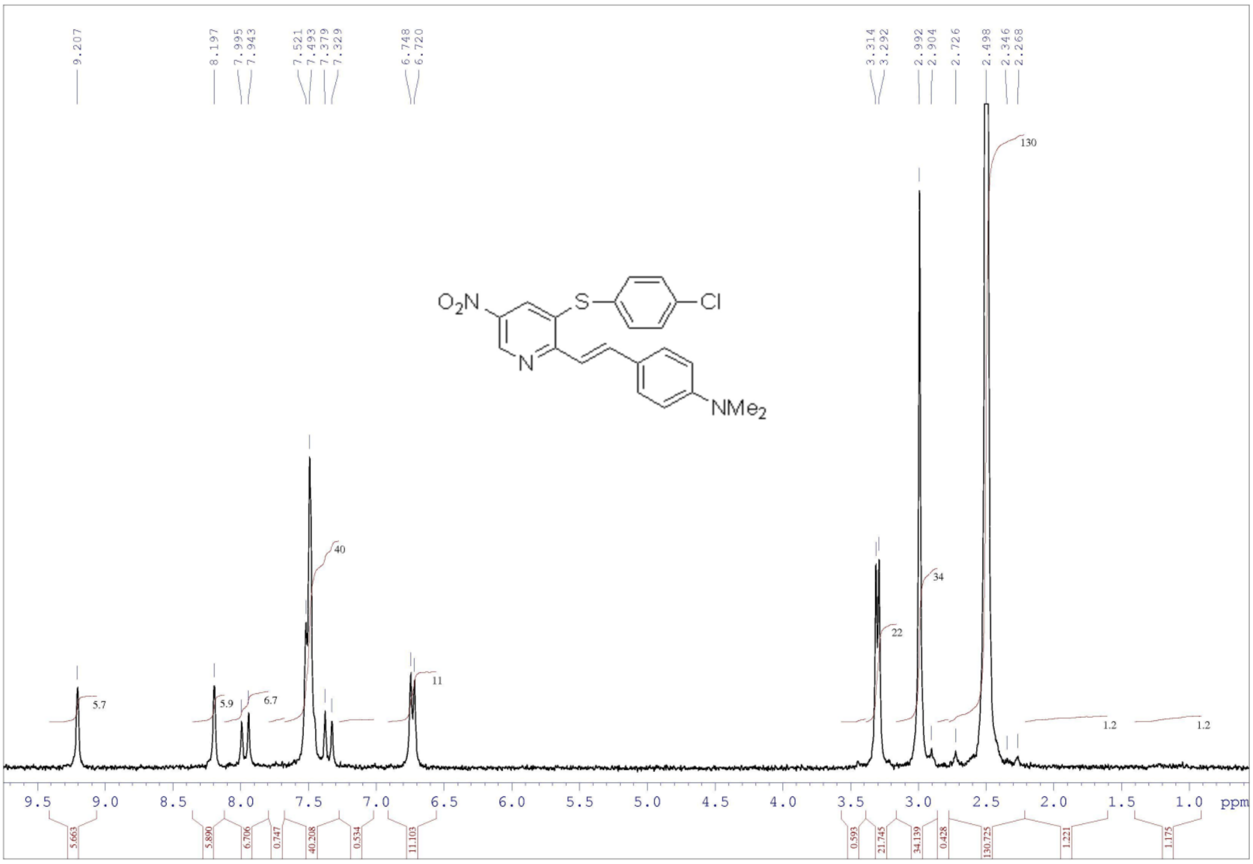

^{13}C NMR spectrum of compound **5k** in $CDCl_3$

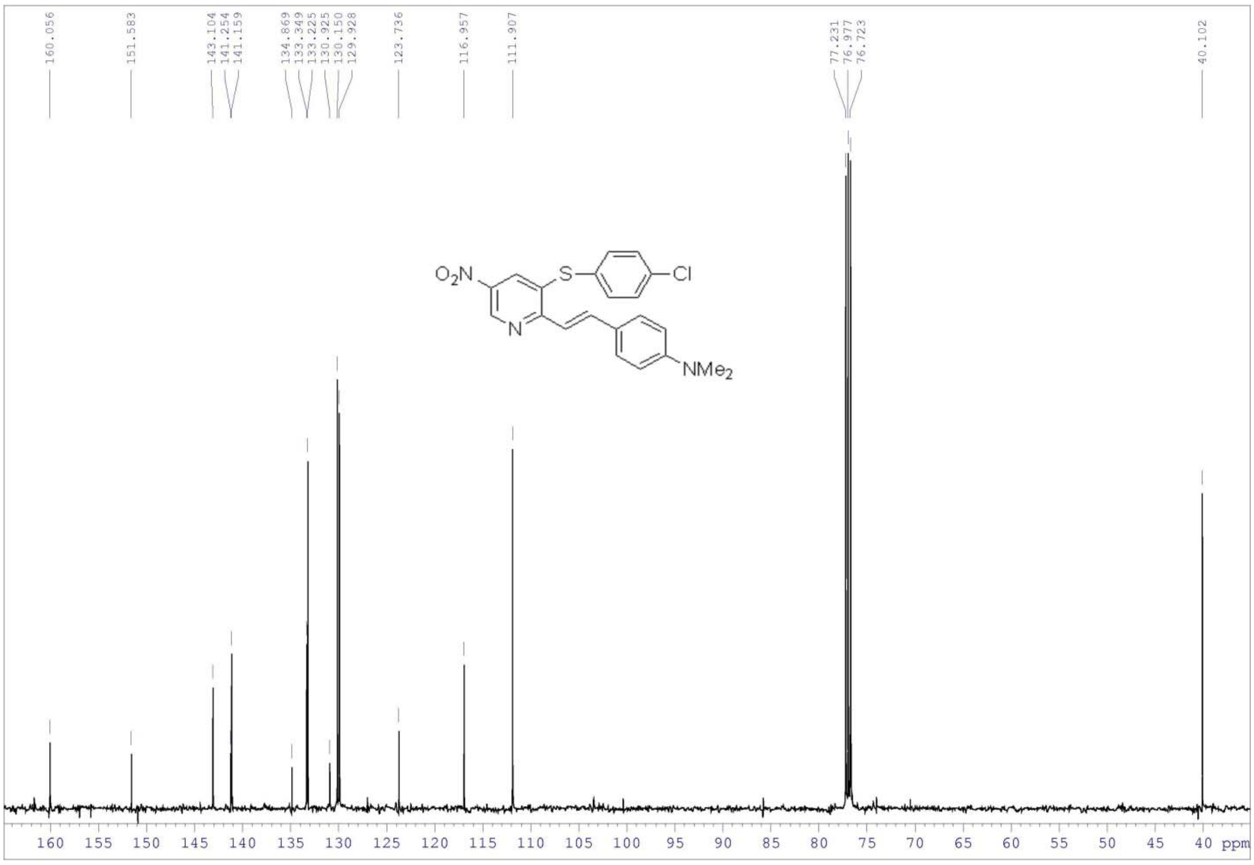

^{1}H and ^{13}C NMR spectra of compound **5l** in $CDCl_3$

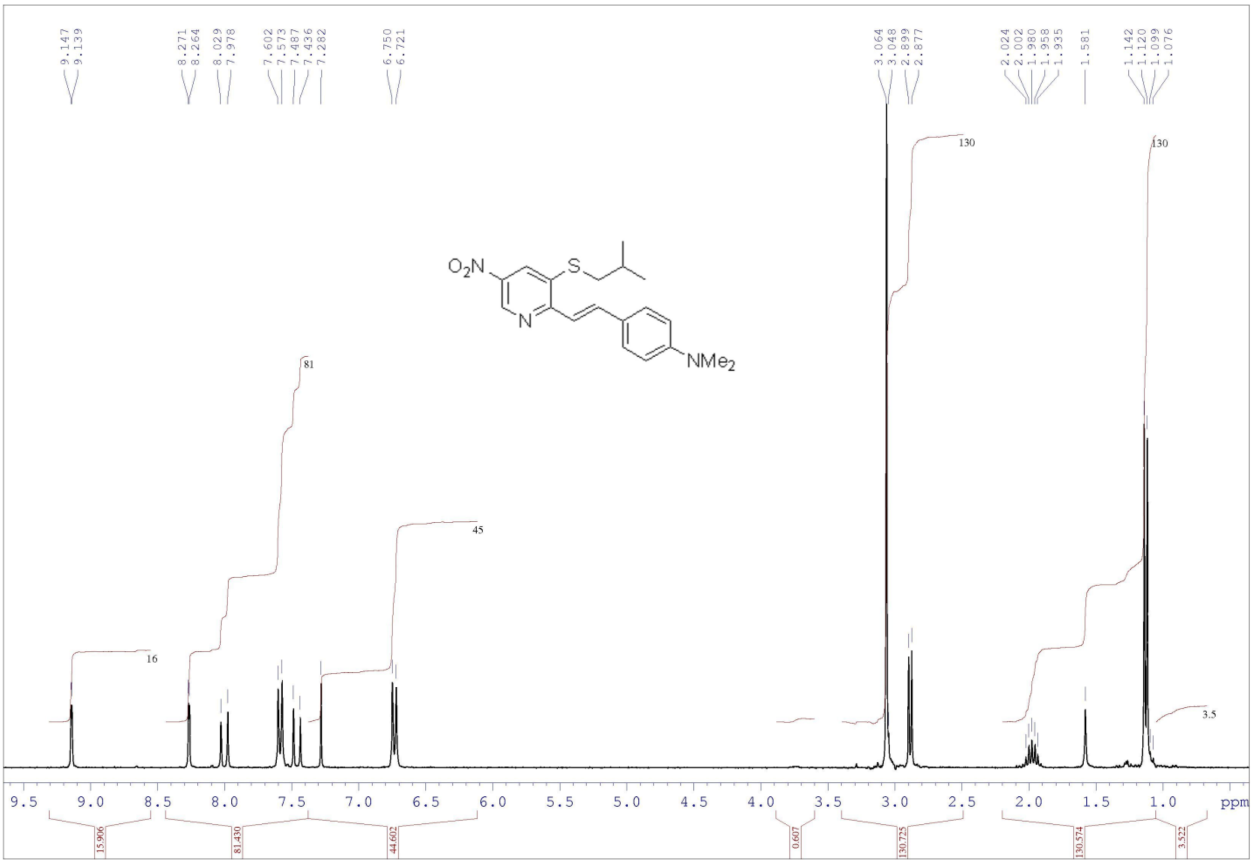

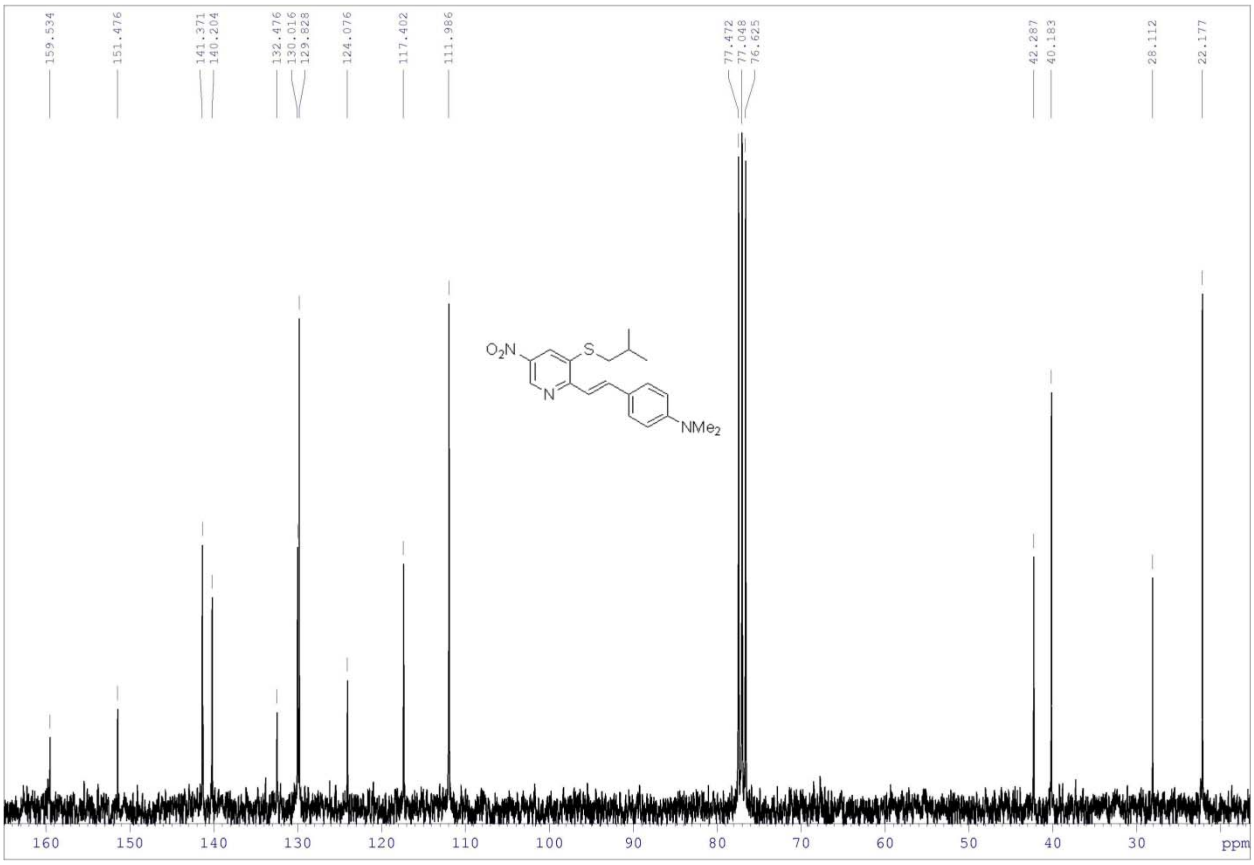

^{1}H and ^{13}C NMR spectra of compound **5m** in $CDCl_3$

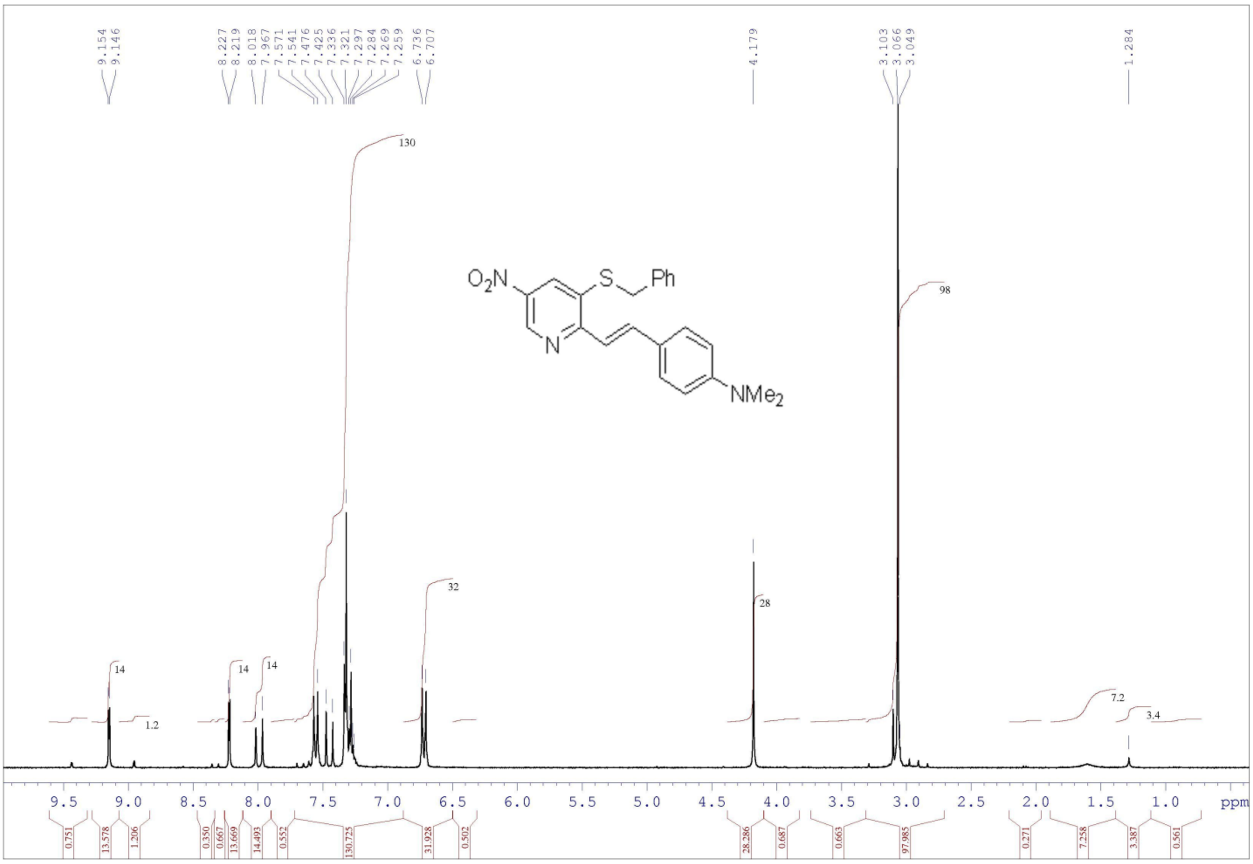

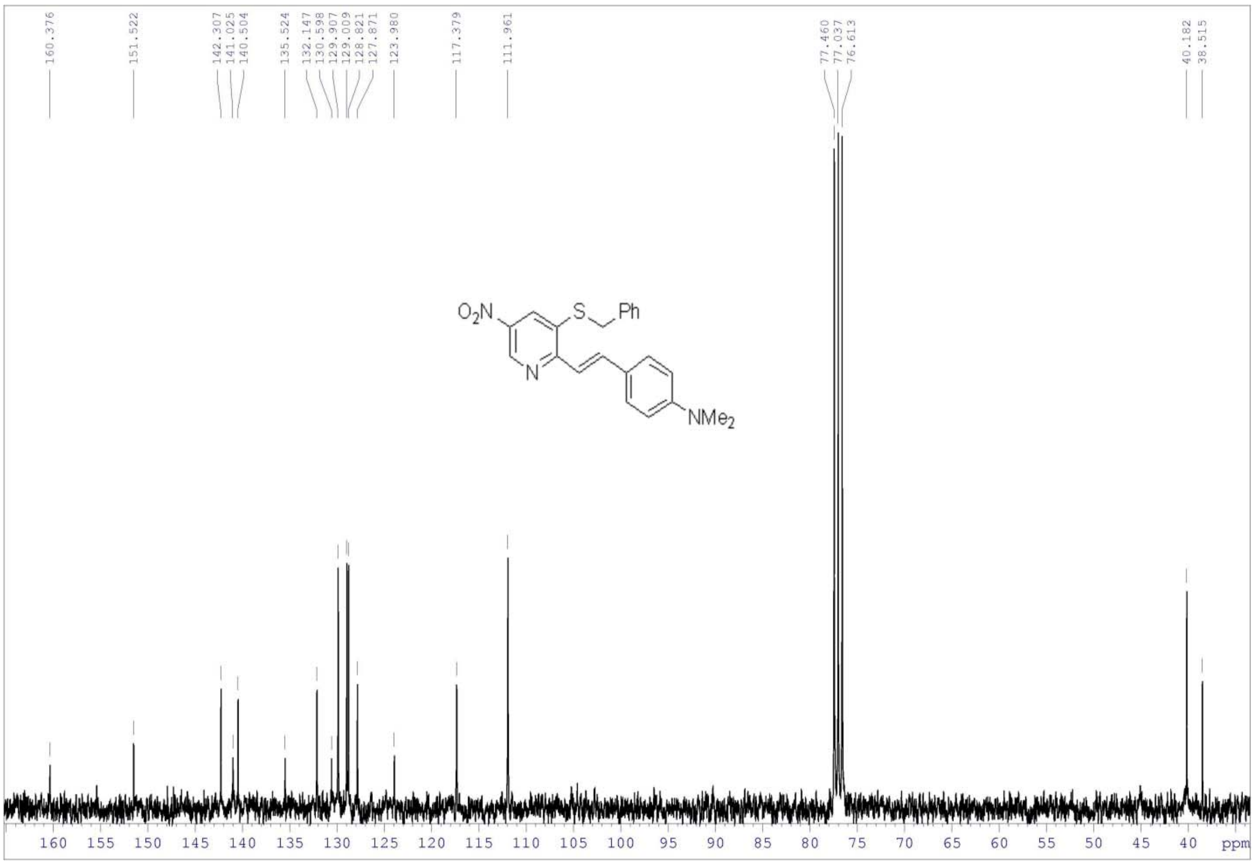

^{1}H and ^{13}C NMR spectra of compound **5n** in $CDCl_3$

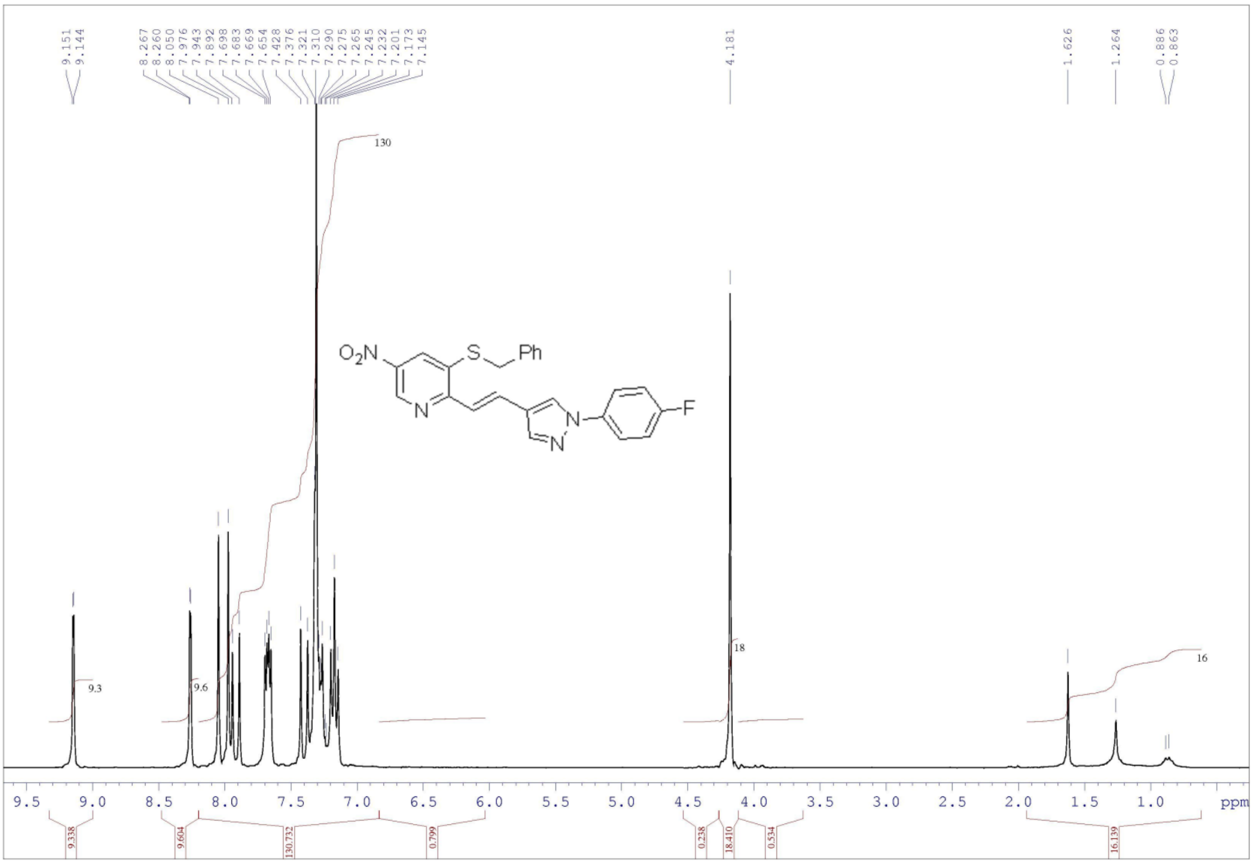

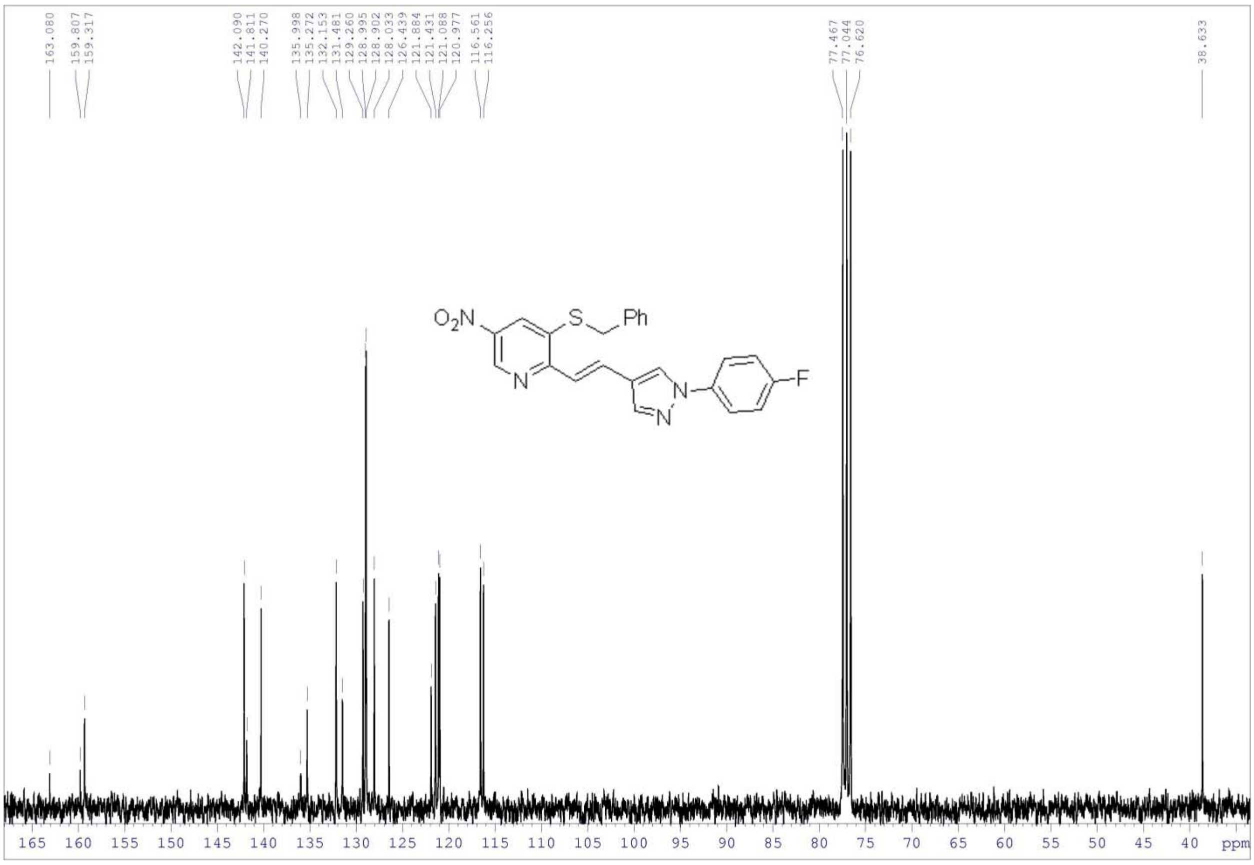

^{1}H NMR spectrum of compound **5o** in DMSO-d_6

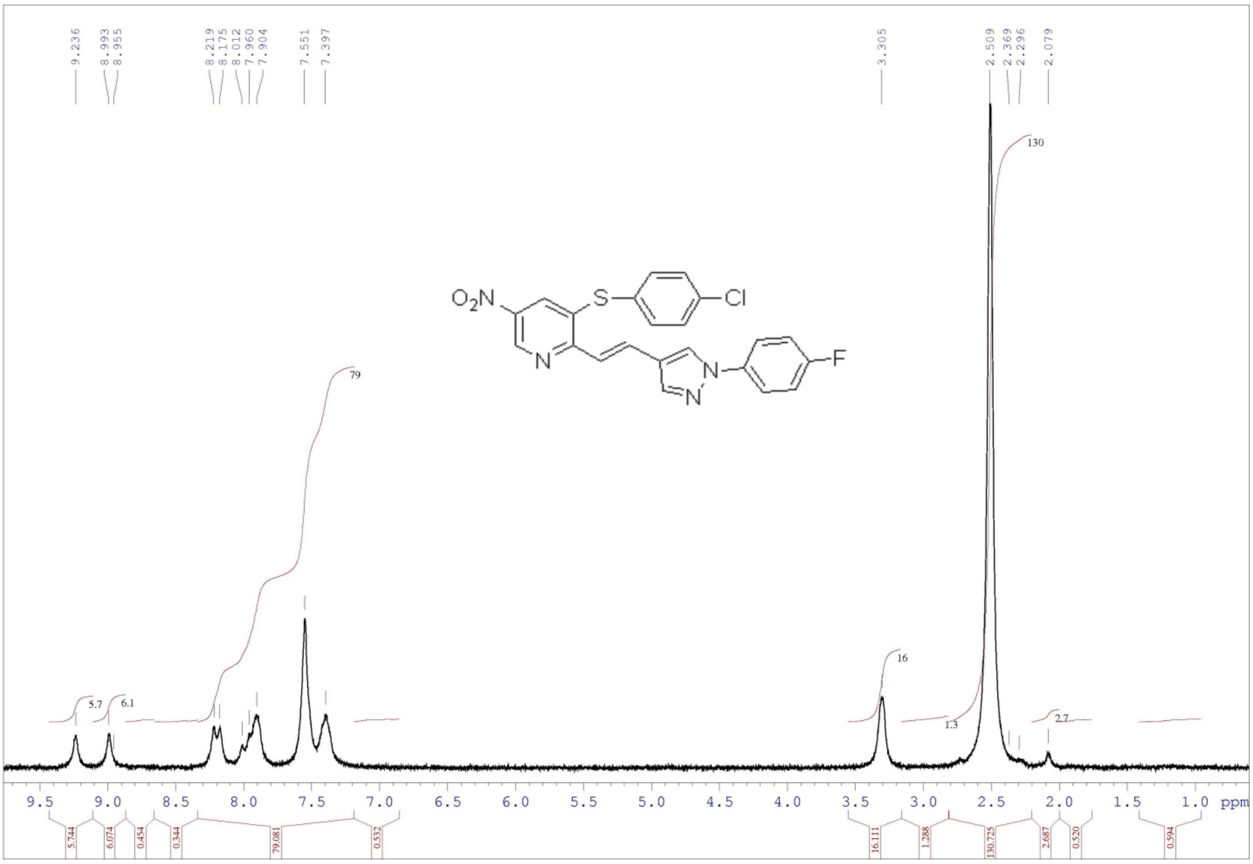

^{1}H and ^{13}C NMR spectra of compound **5p** in $CDCl_3$

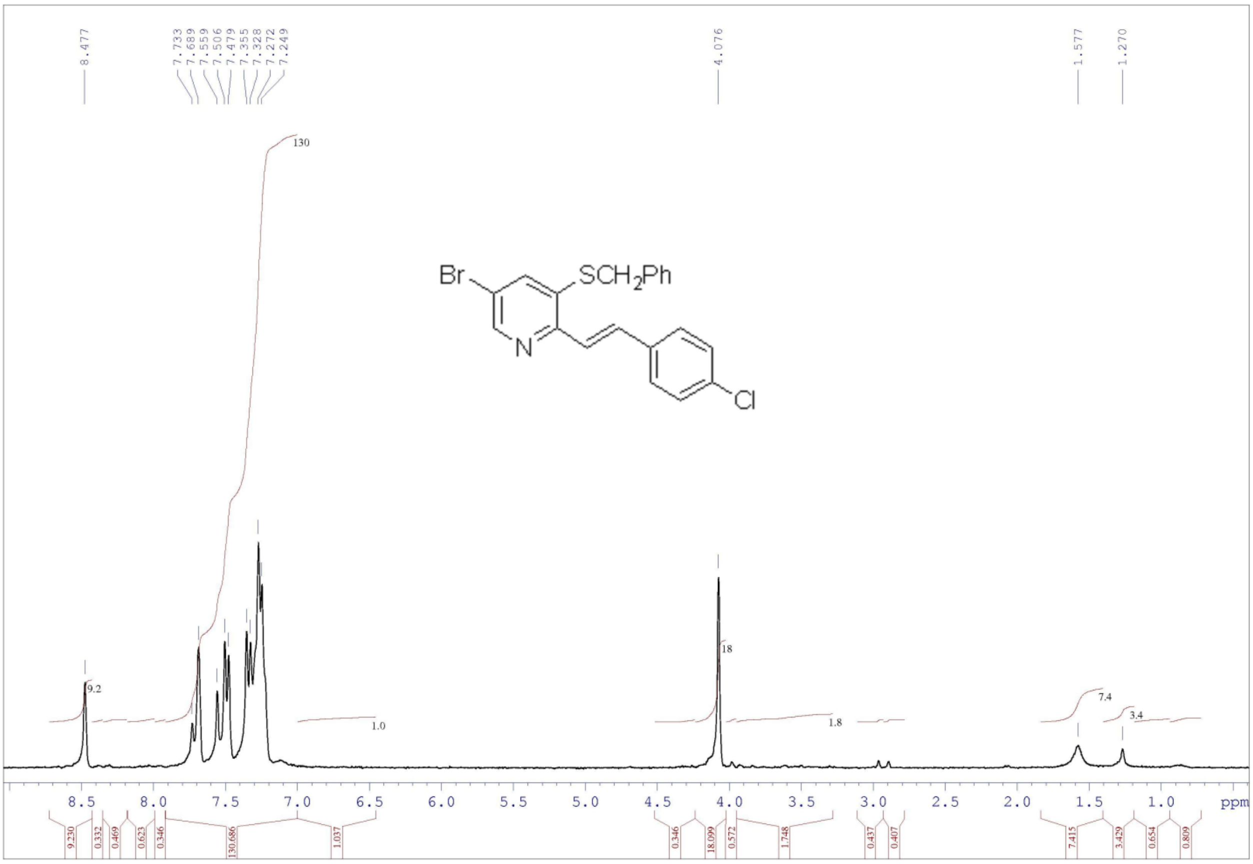

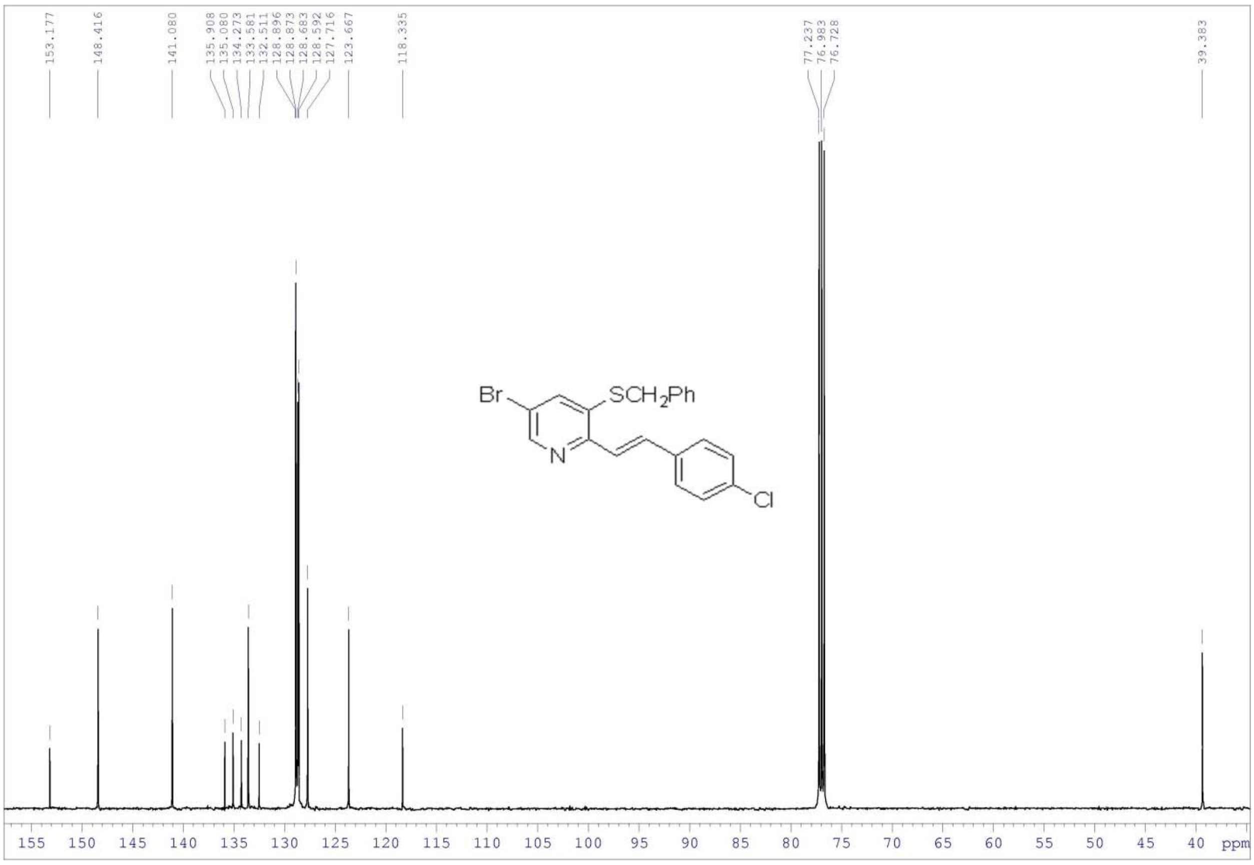

^{1}H and ^{13}C NMR spectra of compound **5q** in $CDCl_3$

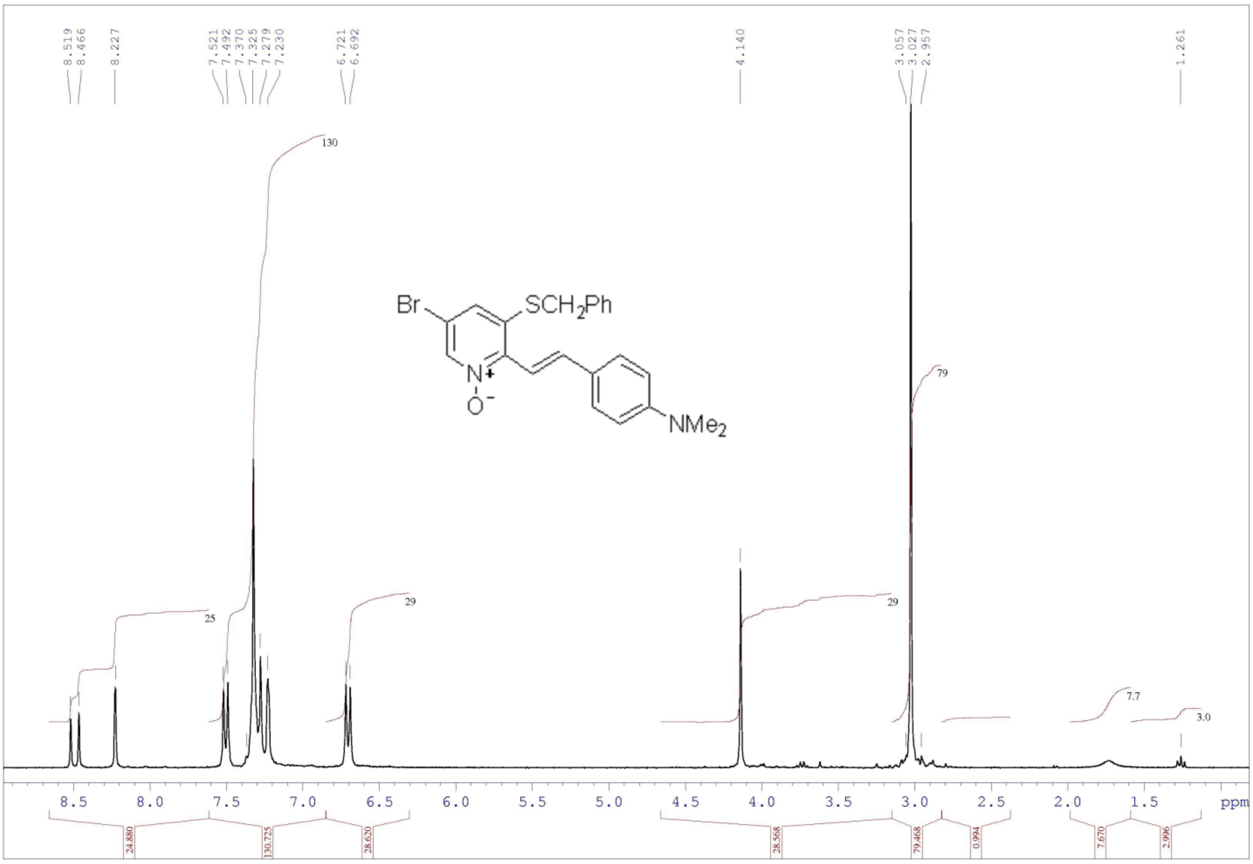

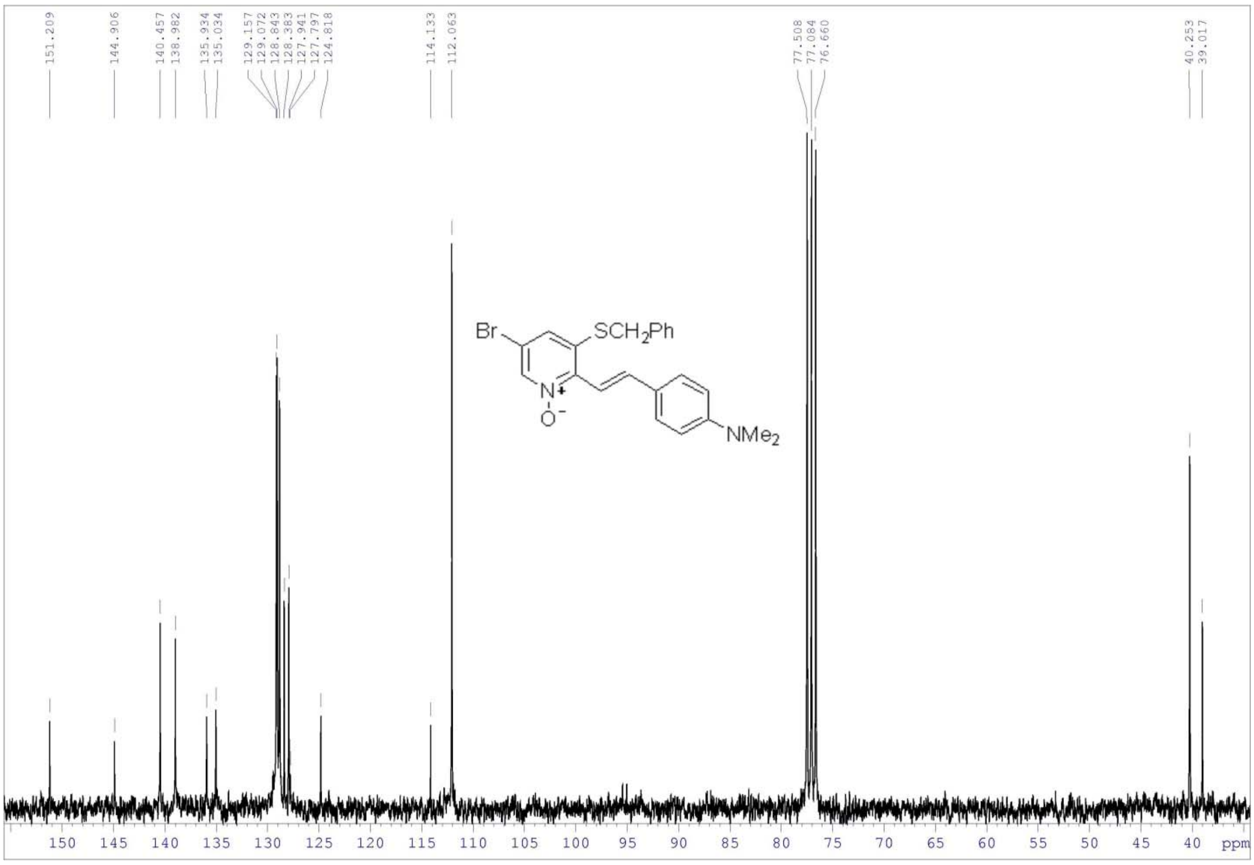

^{1}H NMR spectrum of compound **6g** in $CDCl_3$

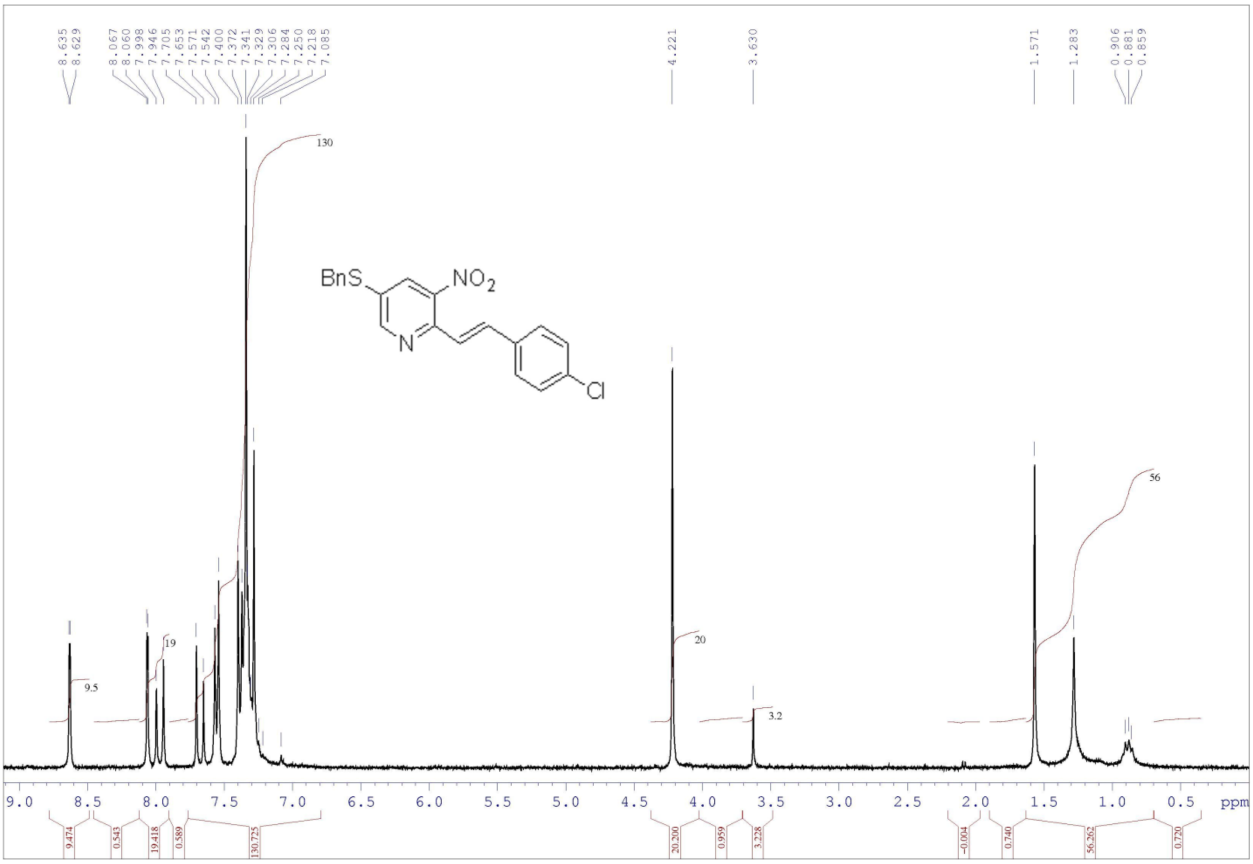

^{1}H and ^{13}C NMR spectra of compound **6h** in $CDCl_3$

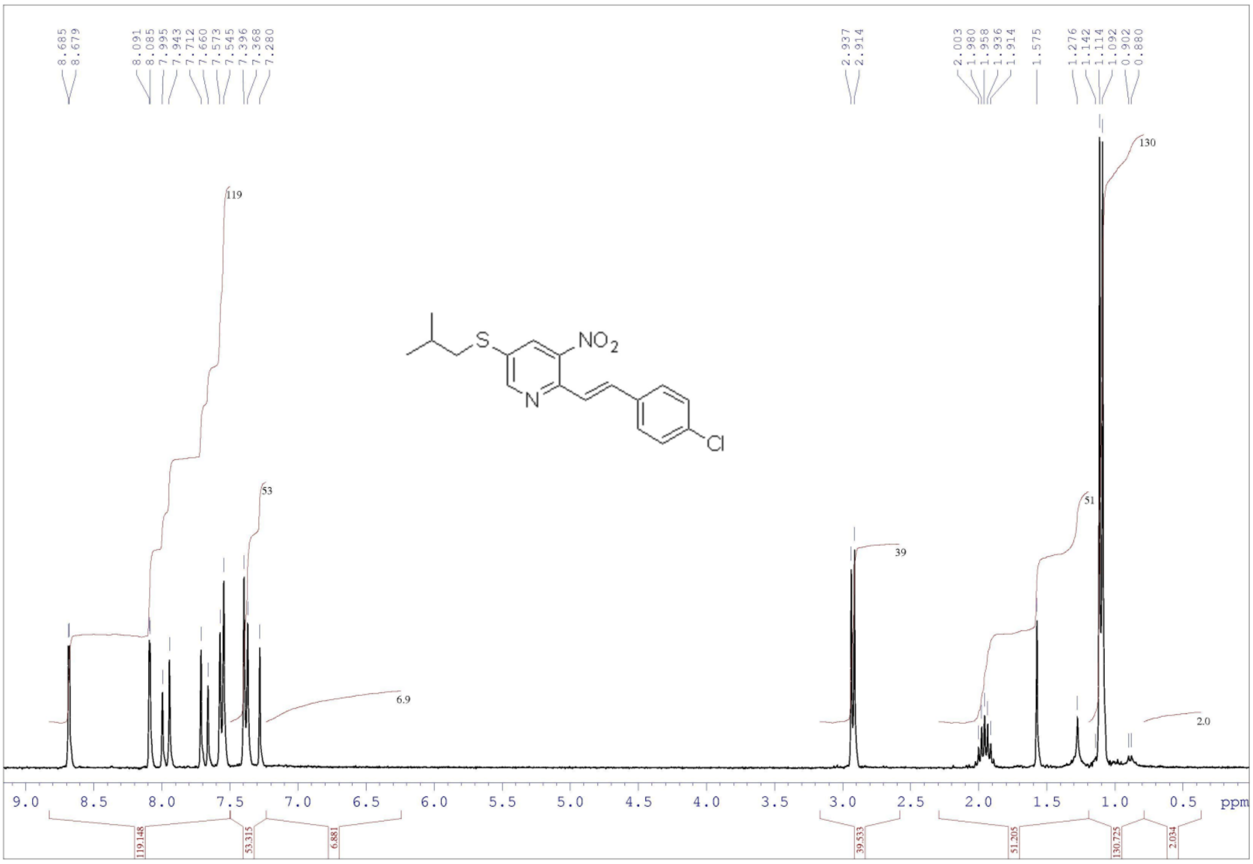

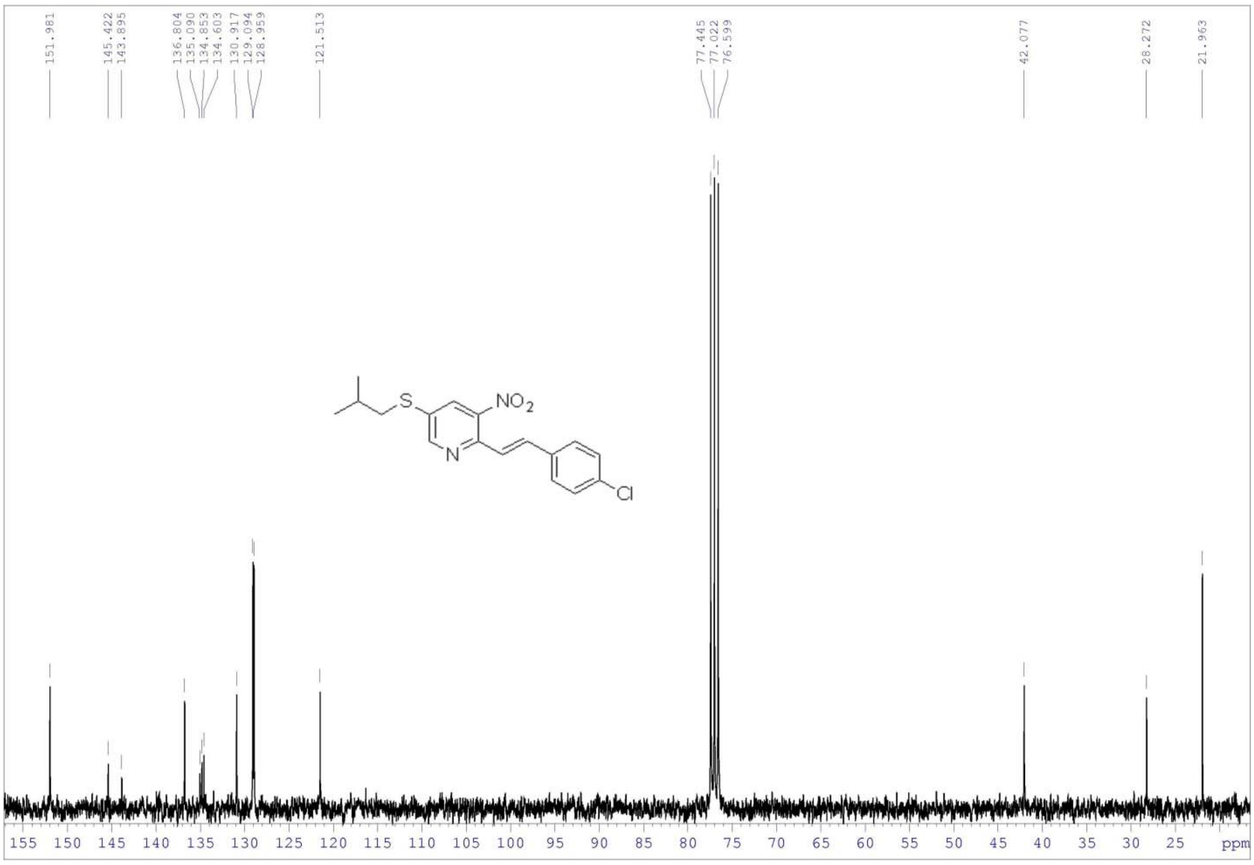

Supplement: Supplementary file 1 [file molecules-27-05692-s001.zip › molecules-1876630-supplementary.pdf]
